# A framework for evaluating epidemic forecasts

**DOI:** 10.1186/s12879-017-2365-1

**Published:** 2017-05-15

**Authors:** Farzaneh Sadat Tabataba, Prithwish Chakraborty, Naren Ramakrishnan, Srinivasan Venkatramanan, Jiangzhuo Chen, Bryan Lewis, Madhav Marathe

**Affiliations:** 1 Computer Science Department, Virginia Tech, 2202 Kraft Drive, Blacksburg/Virginia, 24060 USA; 2 Network Dynamics and Simulation Science Laboratory (NDSSL), Biocomplexity Institute, Virginia Tech, 1015 Life Science Cir, Blacksburg/Virginia, 24061 USA

**Keywords:** Epidemic forecasting, Error Measure, Performance evaluation, Epidemic-Features, Ranking

## Abstract

**Background:**

Over the past few decades, numerous forecasting methods have been proposed in the field of epidemic forecasting. Such methods can be classified into different categories such as deterministic vs. probabilistic, comparative methods vs. generative methods, and so on. In some of the more popular comparative methods, researchers compare observed epidemiological data from the early stages of an outbreak with the output of proposed models to forecast the future trend and prevalence of the pandemic. A significant problem in this area is the lack of standard well-defined evaluation measures to select the best algorithm among different ones, as well as for selecting the best possible configuration for a particular algorithm.

**Results:**

In this paper we present an evaluation framework which allows for combining different features, error measures, and ranking schema to evaluate forecasts. We describe the various epidemic features (Epi-features) included to characterize the output of forecasting methods and provide suitable error measures that could be used to evaluate the accuracy of the methods with respect to these Epi-features. We focus on long-term predictions rather than short-term forecasting and demonstrate the utility of the framework by evaluating six forecasting methods for predicting influenza in the United States. Our results demonstrate that different error measures lead to different rankings even for a single Epi-feature. Further, our experimental analyses show that no single method dominates the rest in predicting all Epi-features when evaluated across error measures. As an alternative, we provide various Consensus Ranking schema that summarize individual rankings, thus accounting for different error measures. Since each Epi-feature presents a different aspect of the epidemic, multiple methods need to be combined to provide a comprehensive forecast. Thus we call for a more nuanced approach while evaluating epidemic forecasts and we believe that a comprehensive evaluation framework, as presented in this paper, will add value to the computational epidemiology community.

**Electronic supplementary material:**

The online version of this article (doi:10.1186/s12879-017-2365-1) contains supplementary material, which is available to authorized users.

## Background

There is considerable interest in forecasting future trends in diverse fields such as weather, economics and epidemiology [1–6]. Epidemic forecasting, specifically, is of prime importance to epidemiologists and health-care providers, and many forecasting methods have been proposed in this area [7]. Typically, predictive models receive input in the form of a time-series of the epidemiological data from the early stages of an outbreak and are used to predict a few data points in the future and/or the remainder of the season. However, assessing the performance of a forecasting algorithm is a big challenge. Recently, several epidemic forecasting challenges have been organized by the Centers for Disease Control and Prevention (CDC), National Institutes of Health (NIH), Department of Health and Human Services (HHS), National Oceanic and Atmospheric Administration (NOAA), and Defense Advanced Research Projects Agency (DARPA) to encourage different research groups to provide forecasting methods for disease outbreaks such as Flu [8], Ebola [9], Dengue [10, 11] and Chikungunya [12]. Fair evaluation and comparison of the output of different forecasting methods has remained an open question. Three competitions, named Makridakis Competitions (M-Competitions), were held in 1982, 1993, and 2000 to evaluate and compare the performance and accuracy of different time-series forecasting methods [13, 14]. In their analysis, the accuracy of different methods is evaluated by calculating different error measures on business and economic time-series which may be applicable to other disciplines. The target for prediction was economic time-series which have characteristically different behavior compared to those arising in epidemiology. Though their analysis is generic enough, it does not consider properties of the time-series that are epidemiologically relevant. BlackArmstrong [15] provides a thorough summary of the key principles that must be considered while evaluating such forecast methods. Our work expands upon his philosophy of objective evaluation, with specific focus on the domain of epidemiology. To the best of our knowledge, at the time of writing this paper, there have been no formal studies on comparing the standard epidemiologically relevant features across appropriate error measures for evaluating and comparing epidemic forecasting algorithms.

Nsoesie et al. [16] reviewed different studies in the field of forecasting influenza outbreaks and presented the features used to evaluate the performance of proposed methods. Eleven of the sixteen forecasting methods studied by the authors predicted daily/weekly case counts [16]. Some of the studies used various distance functions or errors as a measure of closeness between the predicted and observed time-series. For example, Viboud et al. [17], Aguirre and Gonzalez [18], and Jiang et al. [19] used correlation coefficients to calculate the accuracy of daily or weekly forecasts of influenza case counts. Other studies evaluated the precision and “closeness” of predicted activities to observed values using different statistical measures of error such as root-mean-square-error (RMSE), percentage error [19, 20], etc. However, defining a good distance function which demonstrates closeness between the surveillance and predicted epidemic curves is still a challenge. Moreover, the distance function provides a general comparison between the two time-series and ignores the epidemiological relevance between them, which are more significant and meaningful from the epidemiologist perspective; these features could be better criteria to compare epidemic curves together rather than simple distance error. Cha [21] provided a survey on different distance/similarity functions for calculating the closeness between two time-series or discrete probability density functions. Some other studies have analyzed the overlap or difference between the predicted and observed weekly activities by graphical inspection [22]. Epidemic peak is one of the most important quantities of interest in an outbreak, and its magnitude and timing are important from the perspective of health service providers. Consequently, accurately predicting the peak has been the goal of some forecasting studies [18, 22–30]. Hall et al. [24], Aguirre and Gonzalez [18] and Hyder et al. [30] predicted the pandemic duration and computed the error between the predicted and real value. A few studies also consider the attack rate for the epidemic season as the feature of interest for their method [20, 26].

### Study objective & summary of results

In this paper, an epidemic forecast generated by a model/data-driven approach is quantified based on epidemiologically relevant features which we refer to as *Epi-features*. Further, the accuracy of a model’s estimate of a particular Epi-feature is quantified by evaluating its error with respect to the Epi-features extracted from the ground truth. This is enabled by using functions that capture their dissimilarity, which we refer to as *error measures*.

**Fig. 1:**
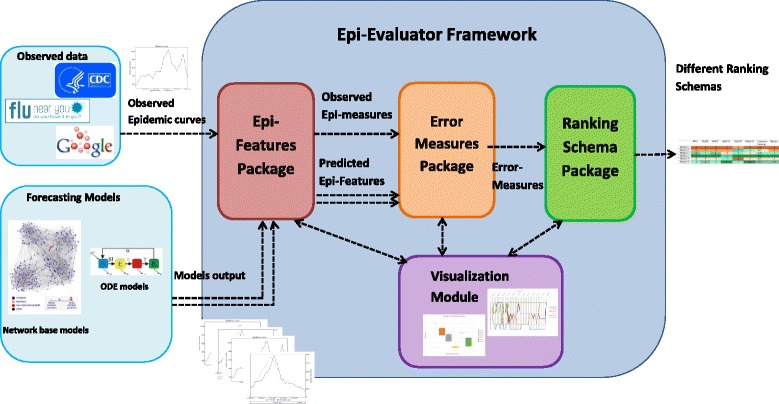
Software Framework: Software Framework contains four packages: Epi-features package, Error Measure package, Ranking schema and Visualization module. The packages are independent and are only connected through the exchanged data

We present a simple end to end framework for evaluating epidemic forecasts, keeping in mind the variety of epidemic features and error measures that can be used to quantify their performance. The software framework, Epi-Evaluator (shown in Fig. 1), is built by taking into account several possible use cases and expected to be a growing lightweight library of loosely coupled scripts. To demonstrate its potential and flexibility, we use the framework on a collection of six different methods used to predict influenza in the United States. In addition to quantifying the performance of each method, we also show how the framework allows for comparison among the methods by ranking them.

We used influenza surveillance data, as reported by the United States Centers for Disease Control and Prevention (CDC) [31], as the gold standard epidemiological data. Output of six forecasting methods was used as the predicted data. We calculated 8 Epi-features on the 2013-2014 season data against 10 HHS regions of the United States (provided by the U.S. Department of Health & Human Services) [32] and 6 error measures to assess the Epi-features. We applied the proposed Epi-features and error measures on both real and predicted data to compare them to each other.

As expected, the performance of a particular method depends on the Epi-features and error measures of choice. Our experimental results demonstrate that some algorithms perform well with regard to one Epi-feature, but do not perform well with respect to other ones. It is possible that none of the forecasting algorithms dominate all the other algorithms in every Epi-feature and error measure.

As a single Epi-feature cannot describe all attributes of a forecasting algorithm’s output, all of them should be considered in the ranking process to obtain a comprehensive comparison. We suggest aggregation of different error measures in the ranking procedure. To this effect, we show how Consensus Ranking could be used to provide comprehensive evaluation. In addition, depending on the purpose of the forecasting algorithm, some Epi-features could be considered more significant than others, and weighted more accordingly while evaluating forecasts. We recommend a second level of Consensus Ranking to accumulate the analysis for various features and provide a total summary of forecasting methods’ capabilities.

We also propose another ranking method, named Horizon Ranking, to provide a comparative evaluation of the methods performance across time. If the Horizon Ranking fluctuates a lot over the time steps, that gives lower credit to the average Consensus Ranking as selection criteria for the best method. Based on experimental results of Horizon Ranking, it is noticed that for a single Epi-feature, one method may show the best performance in early stages of the prediction, whereas another algorithm is the dominator in other time intervals. Finding patterns in Horizon Ranking plots helps in selecting the most appropriate method for different forecasting periods.

Note that many of the proposed Epi-features or error measures have been studied earlier in the literature. The aim of our study is to perform an objective comparison across Epi-features and error measures and ascertain their impact on evaluating and ranking competing models. Further, the focus is not on the performance of methods being compared, but on the features provided by the software framework for evaluating them. The software package is scheduled to be released in an open source environment. We envision it as a growing ecosystem, where end-users, domain experts and statisticians alike, can contribute Epi-features and error measures for performance analysis of forecasting methods.

## Methods

The goal of this paper is to demonstrate how to apply the Epi-features and error measures on the output of a forecasting algorithm to evaluate its performance and compare it with other methods. We implemented a stochastic compartment SEIR algorithm [33] with six different configurations to forecast influenza outbreak (described in the Additional files 1 and 2). These six configurations result in different forecasts which are then used for evaluation. In the following sections, we expand upon the different possibilities we consider for each module (Epi-features, error measures and ranking schema) and demonstrate their effect on evaluating and ranking the forecasting methods.

## Forecasting process

Epidemic data are in the form of a time-series such as *y*(1),…,*y*(*t*),..,*y*(*B* *l* *a* *c* *k* *T*), where *y*(*t*) denotes the number of new infected cases observed in time *t*, and *T* is the duration of the epidemic season. Weekly time-steps are usually preferred to average out the noise in daily case counts.

**Fig. 2:**
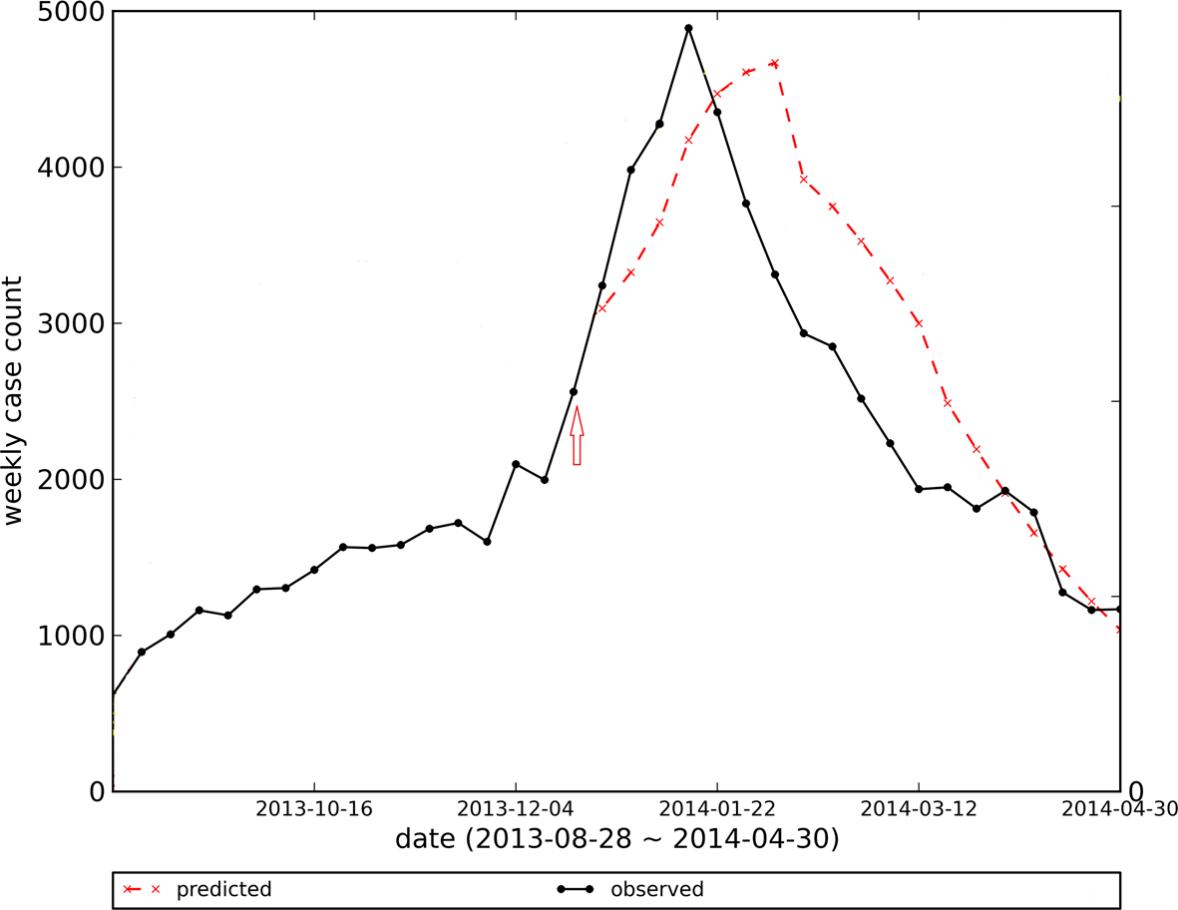
Predicting Epidemic Curve. The red arrow points to the prediction time *k* in which prediction occurs based on *k* initial data points of time-series. The red dashed line is predicted epidemic curve and the black line is observed one

Let us denote the *prediction time* by *k* and the prediction horizon by *w*. Given the early time-series up to time *k* (*y*(1),…,*y*(*k*)) as observed data, the forecasting algorithm predicts the time-series up to the prediction horizon as *x*(*k*+1),…,*x*(*k*+*w*). The forecasts could be short-term (small *w*), or long-term (*w*=*T*−*k*). As most of the proposed Epi-features are only defined based on the complete epidemic curve rather than a few predicted data points, we generate long-term forecasts for each prediction time. The remainder of the observed time-series (*y*(*k*+1),…,*y*(*T*)) is used as a test set for comparing with the predicted time-series (Fig. 2). We increment the prediction time *k*, and update the predictions as we observe newer data points. For each prediction time *k*, we generate an epidemic curve for the remainder of the season.

## Epidemiologically relevant features

In this section, we list the Epi-features we will use to characterize the features of an epidemic time-series. While some of these quantities are generic and applicable to any time-series, the others are specific to epidemiology. Table 1 summarizes the notations needed to define these Epi-features and Table 2 lists the brief definition of them.

**Table 1:** Notation and Symbols

Symbol	Definition
y(t)	number of new cases of disease in the *t* ^*th*^ week observed in surveillance data
x(t)	number of new cases of disease in the *t* ^*th*^ week predicted by forecasting methods
*x*_*start*_	number of new cases of disease predicted at the start of epidemic season
*x*_*peak*_	predicted value of the maximum number of new cases of the disease
*e*_*t*_	*e*_*t*_=*y*_*t*_−*x*_*t*_ : the prediction error
*T*	duration of the epidemic season
$ \bar {y} $	$\bar {y}=\frac {1}{T} \sum _{t=1}^{T} (y_{t}) $ : the mean for *y* values over *T* weeks
*σ* ^2^	$ \sigma ^{2}=\frac {1}{T-1} \sum _{t=1}^{T} (y_{t}-\bar {y})^{2} $ : The variance of *y* values over *T* weeks
*n*_*tot*_	Total number of infected persons during specified period
*n*_*ps*_	The population size at the start of specified period
*n*_*tot*_(*a* *g* *e*)	Total number of infected persons with specific age during the specified period
*n*_*ps*_(*a* *g* *e*)	The population size with specific age at the start of specified period
*n*_*c*_	or *n*_*contacts*_ is the number of contacts of primary infected persons
*n*_*sg*_	or *n*_*second*−*generation*_ is the new number of infected persons among the contacts of primary infected individuals during a specified period
*G* *M*{*E* *r* *r* *o* *r*}	$ GM(e)= \left (\prod _{i=1}^{n}(e_{i})\right)^{(1/n)} $ : Geometric Mean of a set of Errors
*M*{*E* *r* *r* *o* *r*}	Arithmetic Mean of a set of Errors
*M* *d*{*E* *r* *r* *o* *r*}	Median value of a set of Errors
*R* *M* *S*{*E* *r* *r* *o* *r*}	Root Mean Square of a set of Errors

**Table 2:** Definitions of different Epidemiologically Relevant features (Epi-features)

Epi-feature name	Definition
Peak value	Maximum number of new infected cases in a given week in the epidemic time-series
Peak time	The week when peak value is attained
Total attack rate	Fraction of individuals ever infected in the whole population
Age-specific attack rate	Fraction of individuals ever infected belonging to a specific age window
First-take-off-(value):	Sharp increase in the number of new infected case counts over a few consecutive weeks
First-take-off-(time):	The start time of sudden increase in the number of new infected case counts
Intensity duration	The number of weeks (usually consecutive) where the number of new infected case counts is more than a specific threshold
Speed of epidemic	Rate at which the case counts approach the peak value
Start-time of disease season	Time at which the fraction of infected individuals exceeds a specific threshold

### Peak value & time

Peak value is the highest value in a time-series. In the epidemic context, it refers to the highest number of newly infected individuals at any given week during an epidemic season. Closely associated with peak value is peak time, which is the week in which the peak value is attained. Predicting these values accurately helps the healthcare providers with resource planning.

### First-take-off (value & time):

Seasonal outbreaks, like the flu, usually remain dormant and exhibit a sharp rise in the number of cases just as the season commences. A similar phenomenon of sharp increase is exhibited by emerging infectious diseases. The early detection of “first-take-off” time, will help the authorities alert the public and raise awareness. Mathematically, it is the time at which the first derivative of the epidemic curve exceeds a specific threshold. Since the epidemic curve is discretized in weekly increments, the approximate slope of the curve over *Δ* *t* time steps is defined as follows:

1 $$\begin{array}{@{}rcl@{}} s(x,\Delta t) = \frac{x(t+\Delta t)-x(t)}{\Delta t} \end{array} $$

where *x* is the number of new infected case-counts and *t* indicates the week number. In our experiment, we set *Δ* *t*=2. The value of *s*(*x*,*Δ* *t*) is the slope of the curve and shows the take-off-value while the start time of the take-off indicates the take-off-time. The threshold used in calculating the first-take-off depends on the type of the disease and how aggressive and dangerous the outbreak could be. The epidemiologists determine the threshold value and is also based on the geographic area. In this case, we set the threshold to 150.

### Intensity duration

**Fig. 3:**
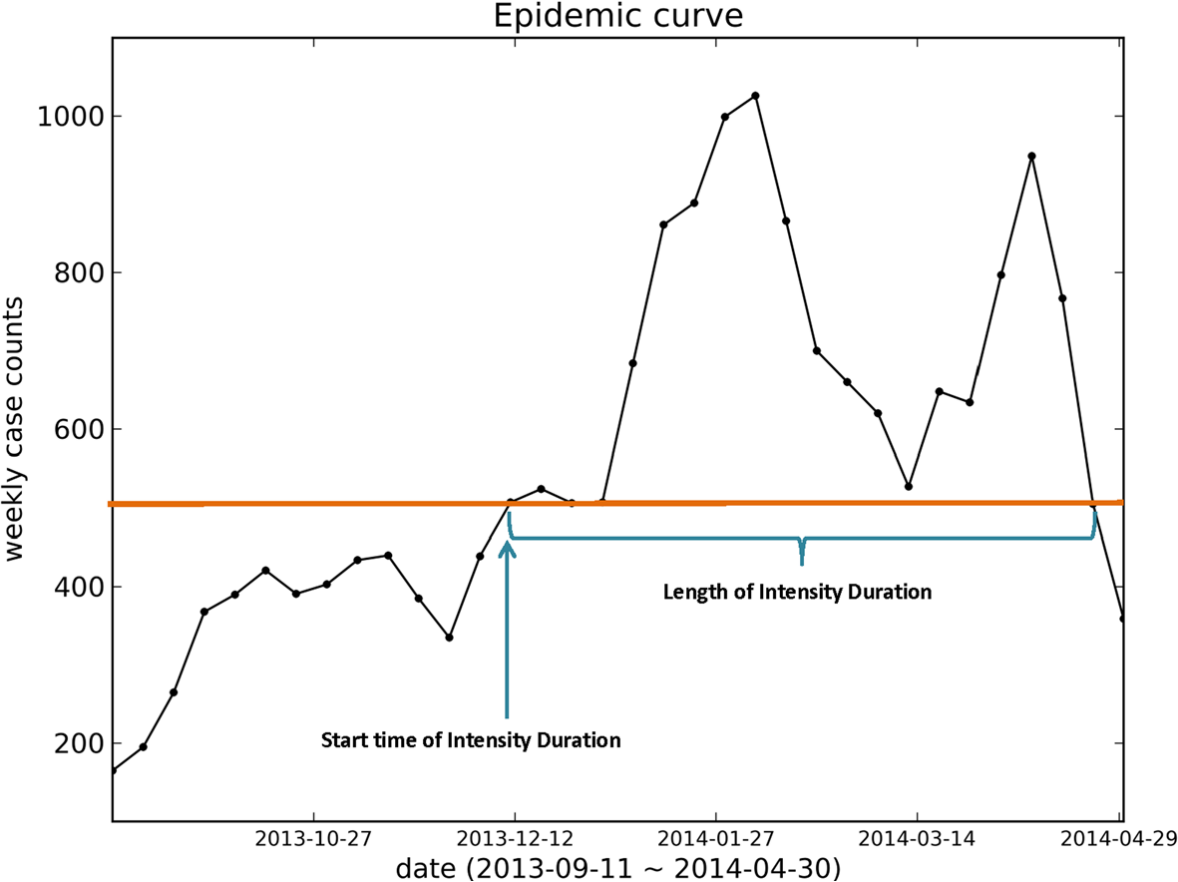
Figure explaining Intensity Duration. Intensity Duration’s length (ID) indicates the number of weeks where the number of new infected case counts are more than a specific threshold

Intensity Duration (ID) indicates the number of weeks, usually consecutive, where the number of new infected case counts is greater than a specific threshold. This feature can be used by hospitals to estimate the number of weeks for which the epidemic will stress their resources (Fig. 3).

### Speed of Epidemic

**Fig. 4:**
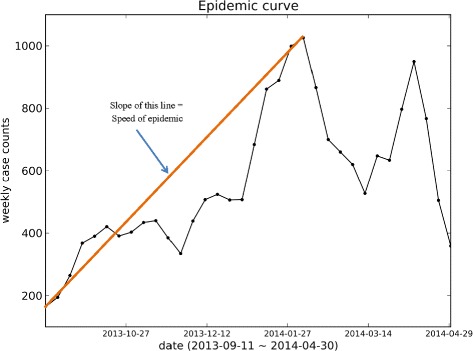
Figure explaining Speed of Epidemic. Speed of Epidemic (SpE) is the steepness of the line that connects the start data-point of time-series sequence to the peak data-point. SpE indicates how fast the infected case counts reach the peak value

The Speed of Epidemic (SpE) indicates how fast the infected case counts reach the peak value. This feature includes peak value and peak time simultaneously. The following equation shows the definition of speed of epidemic:

2 $$ SpE = \frac{x_{peak} - x_{start}}{t_{peak} - t_{start}}  $$

where *x*_*peak*_ and *x*_*start*_ are the number of new case count diseases at peak time and the start time of the season, respectively. In other words, speed of epidemic is the steepness of the line that connects the start data-point of time-series sequence to the peak data-point(Fig. 4).

### Total Attack Rate (TAR):

Attack rate (TAR) is the ratio of the total number of infected cases during a specified period, usually one season, to the size of the whole population at the start of the period.

3 $$\begin{array}{@{}rcl@{}}  \centering TAR= \frac{n_{tot}}{n_{ps}} \end{array} $$

where *n*_*t*_ is the total number of infected people during specified period.

### Age-specific Attack Rate (Age-AR)

This is similar to the total attack rate but focuses on a specific sub-population. Specific attack rate is not only limited to age-specific attack rate, but the sub-population could be restricted by any feature like age, gender, or any special group.

4 $$\begin{array}{@{}rcl@{}} AgeAR\left(age\right) = \frac{n_{tot}(age)}{n_{ps}\left(age\right)} \end{array} $$

### Secondary Attack Rate (SAR):

Secondary attack rate (SAR) means the ratio of new infected cases of a disease, during a particular period, among the contacts of primary cases who are infected first; in other words, it is a measure of the spreading of disease in the contact network.

5 $$\begin{array}{@{}rcl@{}} SAR = \frac{n_{sg} }{n_{c}} \end{array} $$

where *n*_*c*_ is the number of contacts of primary infected persons and *n*_*sg*_ is the number of infected persons among those contacts during a specified period [34]. In order to calculate the secondary attack rate, individual information about households and their contact networks are needed. Epidemiologists estimated the secondary attack rate in household contacts of several states in the U.S. to be 18*%* to 19*%* for acute-respiratory-illness (ARI) and 8*%* to 12*%* for influenza-like-illness (ILI) [35].

### Start-time of a disease Season

We define the “Start-time of a flu season” as the week when the flu-percentage exceeds a specified threshold. The flu-percentage is defined as follows:

6 $$\begin{array}{@{}rcl@{}} Per\left(Flu\right) = \frac{n_{i}\left(Flu\right) }{n_{i}\left(All\right)} \end{array} $$

where *n*_*i*_(*F* *l* *u*) is weekly influenza related illnesses in *i* ^*th*^ week and *n*_*i*_(*A* *l* *l*) is the weekly number of all patients including non-ILI ones seen by health providers for any reason and/or all specimens tested by clinical laboratories. The value of threshold that is used as the criteria is determined by the epidemiologist and could be calculated in different ways. We define the threshold by analyzing the past flu seasons based on the flu baseline definition given by the CDC [36]. The CDC defines the baseline as the mean percentage of visits for influenza during non-influenza weeks for the previous three seasons plus two standard deviations [36]. The non-influenza weeks are defined as two or more consecutive weeks in which the number of counted ILI diagnoses for each week is less than 2% of total seasonal ILI case counts. The definition of start-of-season could be generalized for any disease such as Ebola, Zika, etc.

## Error measures

The second step of evaluating epidemic forecasting algorithms is to measure the error for each predicted Epi-feature. There are a variety of measures that can be used to assess the error between the predicted time-series and the observed one. The error measures that we consider in this study are listed in Table 3 along with their features. The notations used in the error measure equations are described in Table 1. Note that all the error measures considered only handle the absolute value of the error. They do not distinguish between under and over-estimation of the time-series. The signed versions of some of these absolute error measures are listed in the supporting information. These signed measures include the direction of error (i.e. the positive sign demonstrates the underestimation while the negative one indicates overestimation). Moreover, all the measures referred to in Table 3 use Arithmetic Mean to get an average value of the error. Variants that use geometric mean, median, etc. are listed in the Additional file 2: Table S11.

**Table 3:** List of main Error Measures. Arithmetic mean and absolute errors are used to calculate these measures in which positive and negative deviations do not cancel each other out and measures do not provide any information about the direction of errors

Measure name	Formula	Description	Scaled	Outlier Protection	Other forms	Penalize extreme deviation	Other Specification
Mean Absolute Error (MAE)	$ MAE=\frac {1}{T} \sum _{t=1}^{T} |e_{t}| $	Demonstrates the magnitude of overall error	No	Not Good	GMAE	No	-
Root Mean Squared Error (RMSE)	$ RMSE= \sqrt {\frac {\sum _{t=1}^{T} e_{t}^{2}}{T} } $	Root square of average squared error	No	Not Good	MSE	Yes	-
Mean Absolute Percentage Error (MAPE)	$ MAPE=\frac {1}{T} \sum _{t=1}^{T} |\frac {e_{t}}{y_{t}}| $	Measures the average of absolute percentage error	Yes	Not Good	MdAPE ^*a*^, RMSPE ^*b*^	No	-
symmetric Mean Absolute Percentage Error (sMAPE)	$ sMAPE=\frac {2}{T} \sum _{t=1}^{T} |\frac {e_{t}}{y_{t}+x_{t}}| $	Scale the error by dividing it by the average of *y*_*t*_ and *x*_*t*_	Yes	Good	MdsAPE	No	Less possibility of division by zero rather than MAPE.
Mean Absolute Relative Error (MARE)	$ MARE=\frac {1}{T} \sum _{t=1}^{T} |\frac {e_{t}}{e_{RWt}}| $	Measures the average ratio of absolute error to Random walk error	Yes	Fair	MdRAE, GMRAE	No	-
Relative Measures: e.g. RelMAE (RMAE)	$RMAE=\frac {MAE}{MAE_{RW}}= \frac {\sum _{t=1}^{T} |e_{t}|}{\sum _{t=1}^{T} |e_{RWt|} }$	Ratio of accumulation of errors to cumulative error of Random Walk method	Yes	Not Good	RelRMSE, LMR [43], RGRMSE [44]	No	-
Mean Absolute Scaled Error (MASE)	$ MASE=\frac {1}{T} \sum _{t=1}^{T} |\frac {e_{t}}{\frac {1}{T-1}\times \sum _{i=2}^{T}|y_{i}-y_{i-1}|}| $	Measures the average ratio of error to average error of one-step Random Walk method	Yes	Fair	RMSSE	No	-
Percent Better (PB)	$ PB=\frac {1}{T} \sum _{t=1}^{T} [I\{e_{t},e_{RW_{t}}\}]$	Demonstrates average number of times that method overcomes the Random Walk method	Yes	Good	-	No	Not good for calibration and close competitive methods.
	$ |e_{s,t}|\leq |e_{RW_{t}}| \leftrightarrow I\{e_{t},e_{RW_{t}}\}=1 $						
Mean Arctangent Absolute Percentage Error (MAAPE)	$ MAAPE=\frac {1}{T} \sum _{t=1}^{T} arctan|\frac {e_{t}}{y_{t}}| $	Calculates the average arctangent of absolute percentage error	Yes	Good	MdAAPE	No	Smooths large errors. Solve division by zero problem.
Normalized Mean Squared Error (NMSE)	$ NMSE=\frac {MSE}{\sigma ^{2}} = \frac {1}{\sigma ^{2} T} \sum _{t=1}^{T} e_{t}^{2} $	Normalized version of MSE: value of error is balanced	No	Not Good	NA	No	Balanced error by dividing by variance of real data.

^*a*^Md represent Median ^*b*^RMS represent Root Mean Square

After careful consideration, we selected MAE, RMSE, MAPE, sMAPE, MdAPE and MdsAPE as the error measures for evaluating the Epi-features. We list our reasons and observations on the eliminated error measures in part B of Additional file 1. Also, instead of using MAPE, we suggest corrected MAPE (cMAPE) to solve the problem of division by zero:

7 $$ cMAPE=\left\{ \begin{array}{ll} \frac {1}{T} \sum_{t=1}^{T} \left|\frac{e_{t}}{y_{t}}\right|,& \text{if~} y_{t}\neq0\\ \frac {1}{T} \sum_{t=1}^{T} \left|\frac{e_{t}}{y_{t}+\epsilon}\right|, & \text{otherwise} \end{array}\right.  $$

where *ε* is a small value. It could be equal to the lowest non-zero value of observed data. We have also added two error measures based on the median: Median Absolute Percentage Error (MdAPE) and Median symmetric Absolute Percentage Error (MdsAPE). However, as median errors have low sensitivity to change in methods, we do not recommend them for isolated use as the selection or calibration criteria.

## Ranking methods

The third step of the evaluation process is ranking different methods based on different Epi-features and the result of different error measures. For this purpose, we have used two kinds of ranking methods: Consensus Ranking and Horizon Ranking.

- **Consensus Ranking**: Consensus Ranking (CR) for each method is defined as the average ranking of the method among others. This kind of Consensus Ranking could be defined in different scenarios. For example, the average ranking that is used in Table 5 in the Result section is Consensus Ranking of a method based on one specific Epi-feature integrated across different error measures.
  8 $$\begin{array}{@{}rcl@{}} CR_{EM}^{m}= \sum_{i=1}^{n_{EM}} \left|\frac{R_{i,m}}{n_{EM}}\right| \end{array} $$
  where *R*_*i*,*m*_ is the individual ranking assigned to method *m* among other methods for predicting one Epi-feature based on error measure *i*, *n*_*EM*_ is the number of error measures, and Consensus Ranking $CR_{EM}^{m}$ is the overall ranking of method m based on different error measures.
  Consensus Ranking could also be defined across different Epi-features. In this case, CR over error measures could be considered as the individual ranking of a method, and the average is calculated over different Epi-features. It is important to consider the variance of ranking and the intensity of quartiles besides the mean value of CR. In the Results section we demonstrate how to process and analyze these rankings in a meaningful way.
- **Horizon Ranking**: While Consensus Ranking considers the average performance of methods over prediction times, Horizon Ranking demonstrates the performance trend of various forecasting methods in predicting a single Epi-feature across different prediction times. First, for each Epi-feature, we compute an error measure like Absolute Percentage Error (APE) or its symmetric variant (sAPE) per prediction time. For each prediction time, APE values of different forecasting methods are sorted from smallest to largest to determine the ranking of the methods. The average value of this ranking over different error measures determines the overall Horizon Ranking of the methods in each time-step.

## Data

The ILI surveillance data used in this paper was obtained from the website of the United States Centers for Disease Control and Prevention (CDC). The information of patient visits to health care providers and hospitals for ILI was collected through the US Outpatient Influenza-like Illness Surveillance Network since 1997 and lagged by two weeks(ILINet) [31, 37]; this Network covers all 50 states, Puerto Rico, the District of Columbia and the U.S. Virgin Islands.

**Fig. 5:**
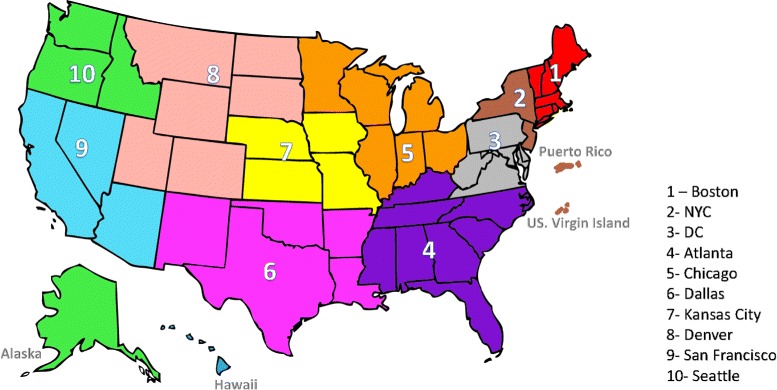
HHS region map, based on “U.S. Department of Health & Human Services” division [32]

The weekly data are separately provided for 10 regions of HHS regions [32] that cover all of the US. The forecasting algorithms have been applied to CDC data for each HSS region. We applied our forecasting algorithm on the 2013-2014 flu season data where every season is less than or equal to one year and contains one major epidemic. Figure 5 shows the HHS Region Map that assigned US states to the regions.

## Results and analysis

Past literature in the area of forecasting provides an overall evaluation for assessing the performance of the predictive algorithm by defining a statistical distance/similarity function to measure the closeness of the predicted epidemic curve to the observed epidemic curve. However, they rarely evaluate the robustness of a method’s performance across epidemic features of interest and error measures. Although the focus of the paper is not on a specific method to be chosen, it is instructive to observe the funtionality of the software framework in action applied on the sample methods.

### Rankings based on error measures applied to peak value

**Fig. 6:**
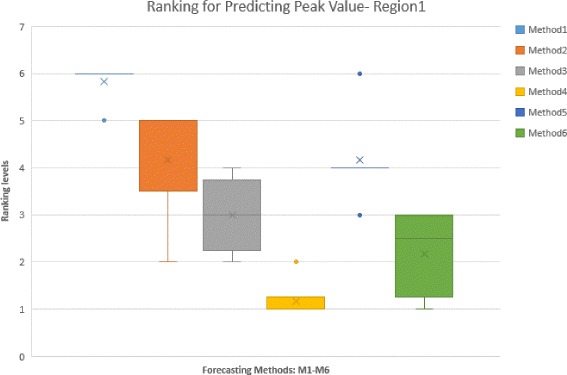
Box-Whisker Plot shows the Consensus Ranking of forecasting methods in predicting Peak value for Region 1, aggregated on different error measures

In Table 4, we calculated six error measures, MAE, RMSE, MAPE, sMAPE, MdAPE, and MdsAPE for the peak value predicted by six different forecasting methods. The corresponding ranks are provided in the Ranking Table (Table 5). The most successful method is assigned rank 1 (R1); As can be seen, even similar measures like MAPE and sMAPE do not behave the same for the ranking process. The fourth algorithm wins six first places among other methods for seven error measures and shows almost the best performance. However, it is hard to come to a similar conclusion for other methods. The last column in the table is Consensus Ranking, which shows the average ranking of the method over different error measures. Figure 6 shows the Box-Whisker diagram of method rankings. Note that, Methods 2 and 5 despite having identical Consensus Ranking, have different interquartile ranges, which represents Method 5 as a more reliable approach. Based on such analysis, the fourth method (M4) is the superior for predicting the peak value. After that, the order of performance for other methods will be: Method 6 (M6), Method 3, Method 5, Method 2 and Method 1. Note however, this analysis is specific to using peak value as the Epi-feature of interest.

**Table 4:** Different errors for predicting peak value for Region 1 over whole season (2013-2014)

	MAE	RMSE	MAPE	sMAPE	MdAPE	MdsAPE
Method 1	4992.0	9838.6	4.9	1.04	1.7	1.03
Method 2	4825.2	9770.4	4.7	0.99	1.4	0.95
Method 3	3263.0	5146.5	3.2	0.96	1.5	1.01
Method 4	2990.7	4651.3	2.9	0.899	1.1	0.85
Method 5	3523.2	5334.8	3.4	0.95	2.1	1.01
Method 6	3310.9	4948.5	3.2	0.896	1.5	0.85

**Table 5:** Ranking of methods for predicting peak value based on different error measures for Region 1 over whole season (2013-2014). The color spectrum demonstrates different ranking levels. Dark green represents the best rank, whereas dark orange represents the worst one

	MAE	RMSE	MAPE	sMAPE	MdAPE	MdsAPE	Consensus	Median
							Ranking	
Method 1	6	6	6	6	5	6	5.83	6
Method 2	5	5	5	5	2	3	4.17	5
Method 3	2	3	2	4	3	4	3.00	3
Method 4	1	1	1	2	1	1	1.17	1
Method 5	4	4	4	3	6	4	4.17	4
Method 6	3	2	3	1	3	1	2.17	2.5

### Consensus Ranking across all Epi-features

In order to make a comprehensive comparison, we have calculated the error measures on the following Epi-features: Peak value and time, Take-off-value and Take-off-time, Intensity Duration’s length and start time, Speed of epidemic, and start of flu season. We do not include demographic-specific Epi-features, such as age-specific attack rate or secondary attack rate, since such information is not available for our methods.

**Fig. 7:**
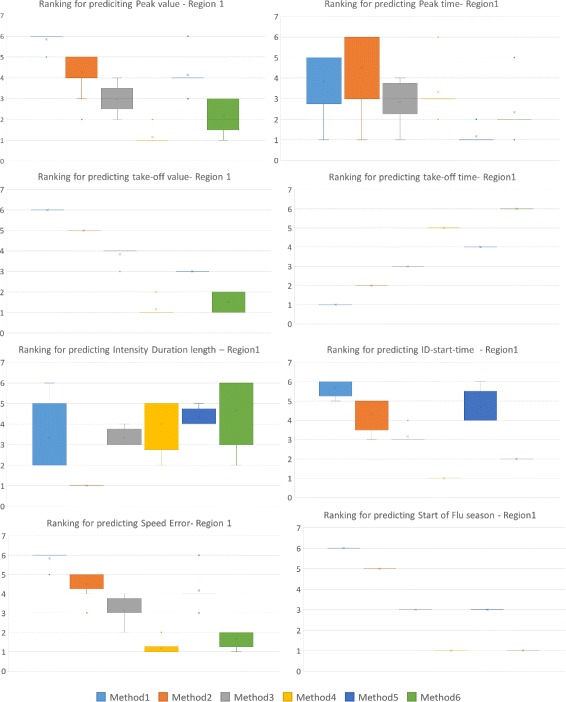
Consensus Ranking of forecasting methods over all error measures for predicting different Epi-features for Region 1. Method 4 is superior in predicting five Epi-features out of eight, but is far behind other methods in predicting three other Epi-features

Figure 7 shows the Consensus Ranking of the methods in predicting different Epi-features for Region 1. Note that Method 4, which is superior in predicting some Epi-features such as Peak value and start of Flu season, is worse than other methods in predicting other Epi-features such as Take-off time and Intensity Duration. The tables corresponding to the box-plots are included in Additional file 2.

**Fig. 8:**
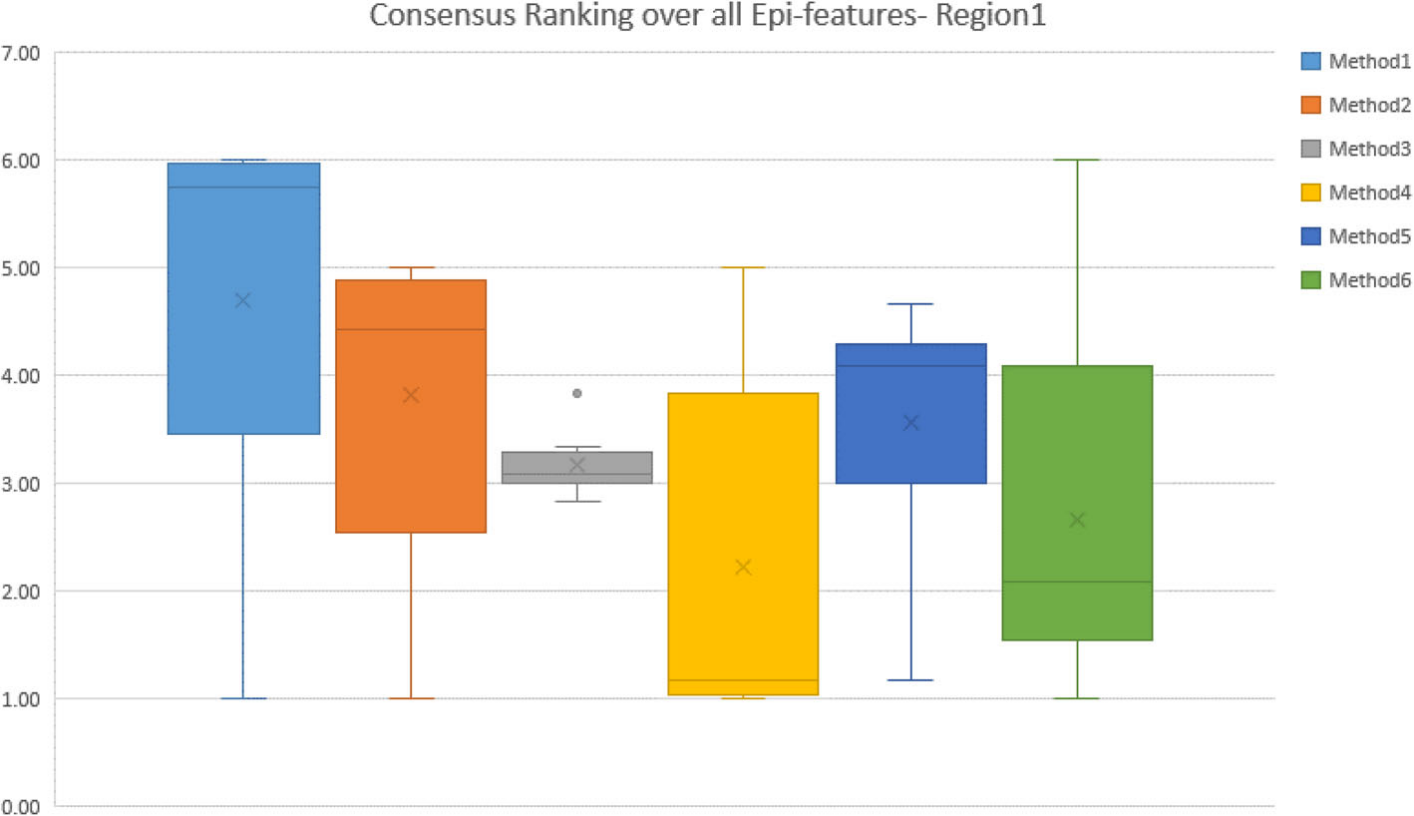
The box-whisker diagrams shows the median, mean and the variance of Consensus Ranking of methods over all Epi-features for Region 1

Figure 8 shows the second level of Consensus Ranking over various Epi-features for Region 1. This figure summarizes the performance of different methods based on the average Consensus Rankings that are listed in Table 6. It is evident that Method 1, Method 2, and Method 5 have similar performance, while the third method performs moderately well across Epi-features. Method 4, which performs best for five out of eight Epi-features, is not among the top three methods for predicting Take-off time and Intensity Duration. Method 6 comes in as the second best method when considering the Consensus Ranking.

**Table 6:** Average Consensus Ranking over different error measures for all Epi-features- Region 1

	Peak value	Peak time	Take-off-value	Take-off-time	ID length	ID start time	Start of flu season	Speed of epidemic	Average	Median
M1	5.83	3.83	6	1	3.33	5.67	6	5.83	4.69	5.67
M2	4.17	4.5	5	2	1	4.33	5.0	4.5	3.81	4.33
M3	3	2.83	3.83	3	3.33	3.17	3	3.17	3.17	3.17
M4	1.17	3.33	1.17	5	4.00	1.0	1	1.17	2.23	1.17
M5	4.17	1.17	3	4	4.33	4.67	3	4.17	3.56	4
M6	2.17	2.33	1.50	6	4.67	2.00	1.00	1.67	2.67	2.17

**Fig. 9:**
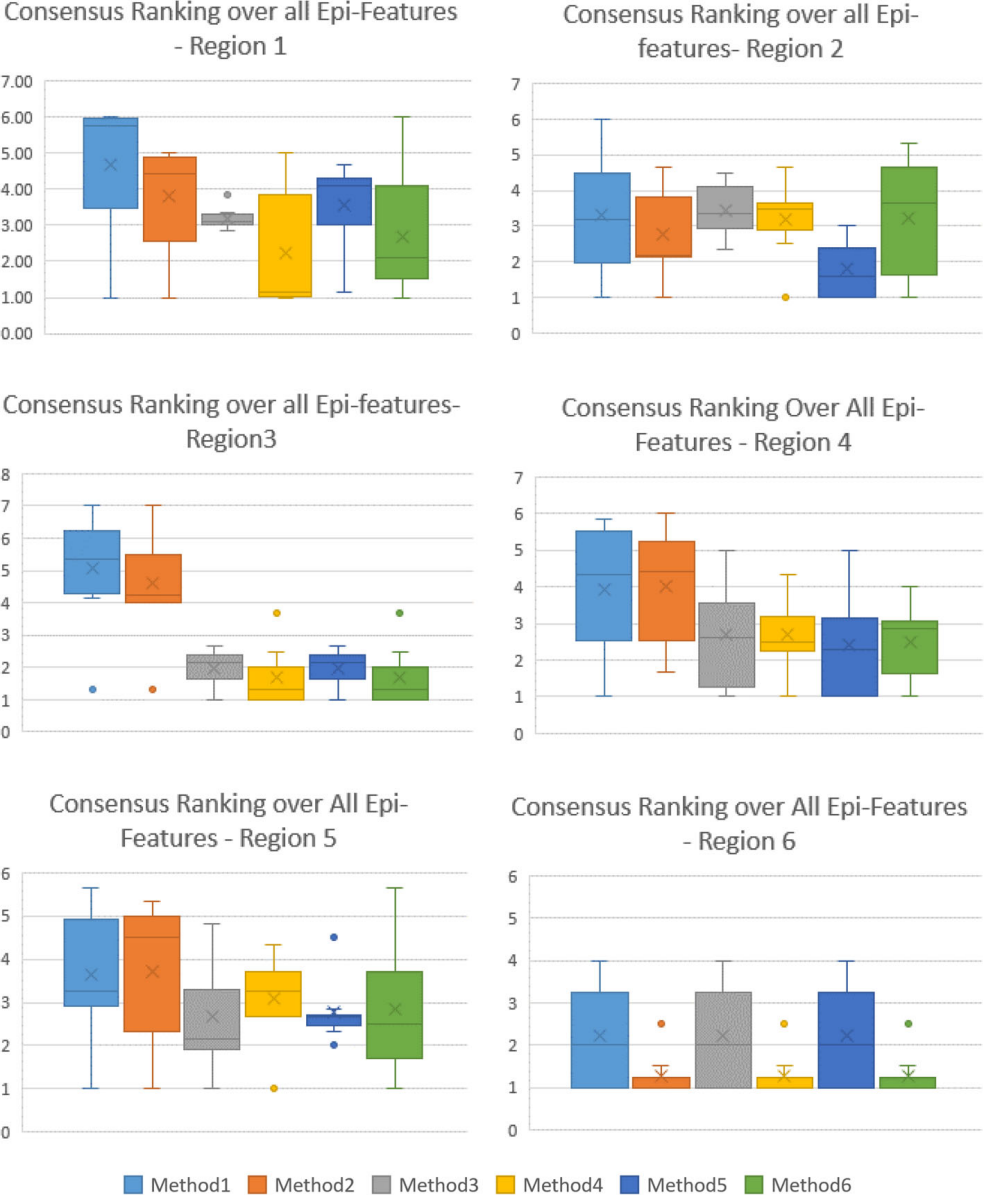
Consensus Ranking over all Epi-Features - Regions 1-6. The box-whisker diagrams show the median, mean and the variance of Consensus Ranking of methods in predicting different Epi-features

**Fig. 10:**
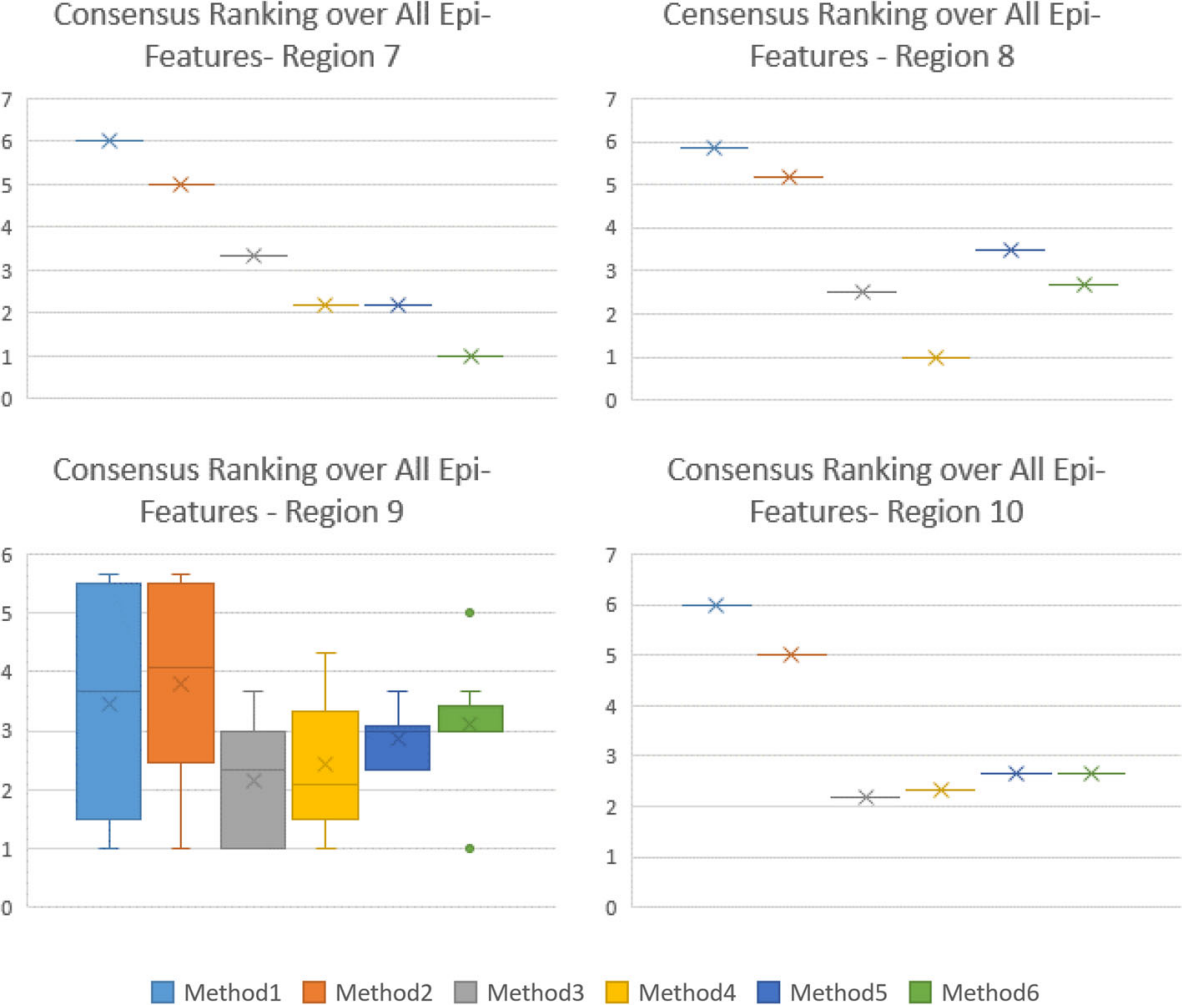
Consensus Ranking over all Epi-Features- Regions 7-10. The box-whisker diagrams show the median, mean and the variance of Consensus Ranking of methods in predicting different Epi-features

**Fig. 11:**
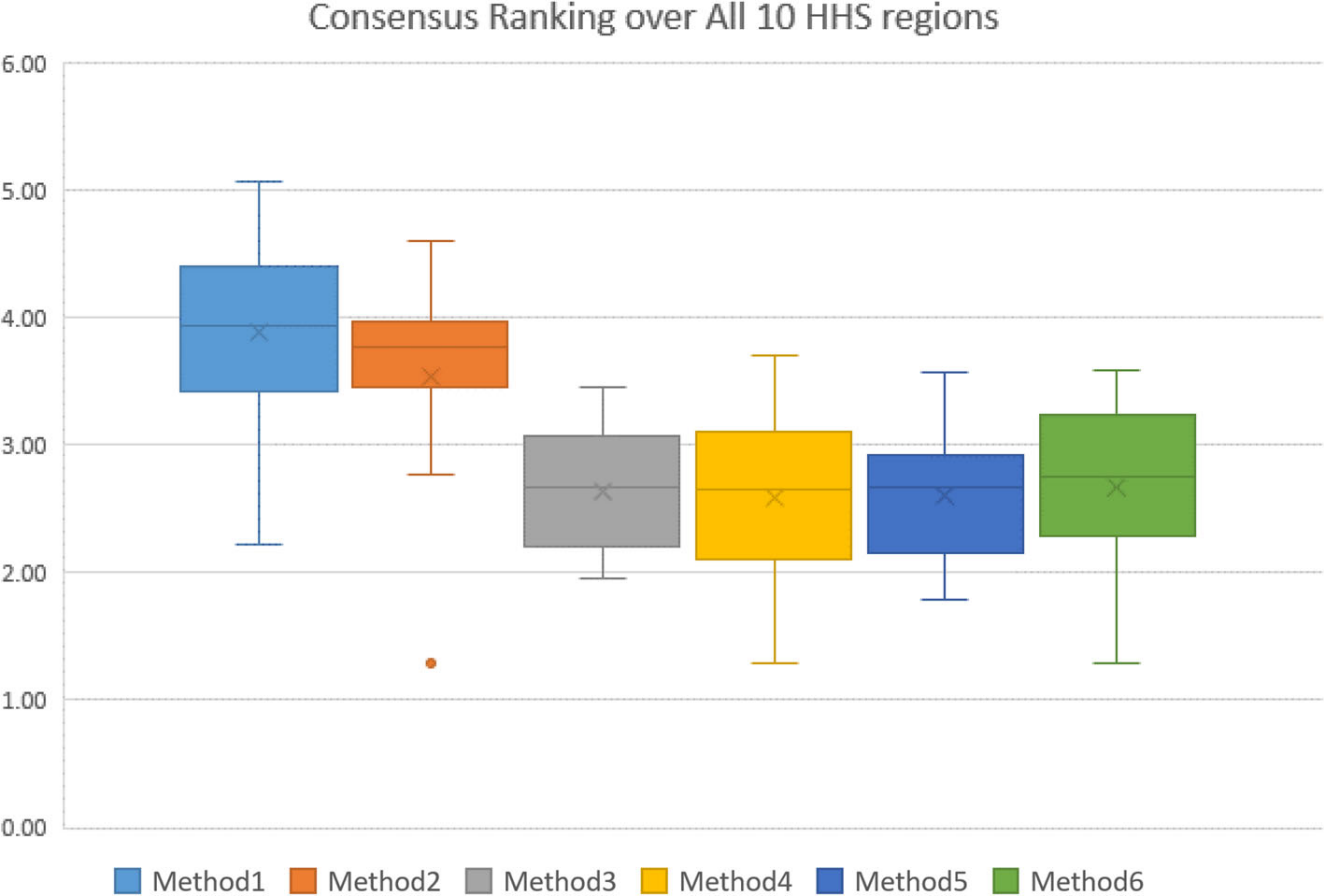
Consensus Ranking over all 10 HHS-Regions. The box-whisker diagrams show the median, mean and the variance of Consensus Ranking of methods in predicting the Epi-features for all HHS regions

The first level of Consensus Ranking over error measures for other HHS regions are included in Additional files 4, 5, 6, 7, 8, 9, 10, 11 and 12, which contain supporting figures S2–S10. Figures 9 and 10 represent the second level of Consensus Rankings of the six approaches over all Epi-features for regions 1 to 10. Often, experts need to select one method as the best predictor for all regions, hence we propose the third level of Consensus Ranking to aggregate the results across different regions. Figure 11 represents the Consensus Ranking over all 10 HHS regions, based on the average of Consensus Rankings across all Epi-features for each region listed in Table 7. As can be seen in Fig. 11, the performance of the first and the second methods are behind the other approaches and we can exclude them from the pool of selected algorithms. However, the other four methods show very competitive performance and are considered the same according to the total rankings. The sequential aggregations provide a general conclusion which eliminates the nuances of similar methods.

**Table 7:** Average Consensus Ranking of methods over different Epi-features- Regions 1 - 10

	Region1	Region2	Region3	Region4	Region5	Region6	Region7	Region8	Region9	Region10	Ave
M1	4.69	3.31	4.6	3.94	3.65	2.21	4.3	3.94	3.46	4.29	3.84
M2	3.81	2.77	4.23	4.0	3.71	1.29	3.73	3.69	3.79	3.96	3.50
M3	3.17	3.46	1.96	2.68	2.67	2.21	3.03	2.73	2.17	2.33	2.64
M4	2.23	3.19	2.04	2.7	3.08	1.29	2.93	2.60	2.44	3.71	2.62
M5	3.56	1.79	1.79	2.41	2.77	2.21	2.67	3.06	2.88	2.67	2.58
M6	2.67	3.23	2.13	2.48	2.83	1.29	2.60	3.27	3.13	3.58	2.72

### Horizon Rankings for each Epi-feature

**Fig. 12:**
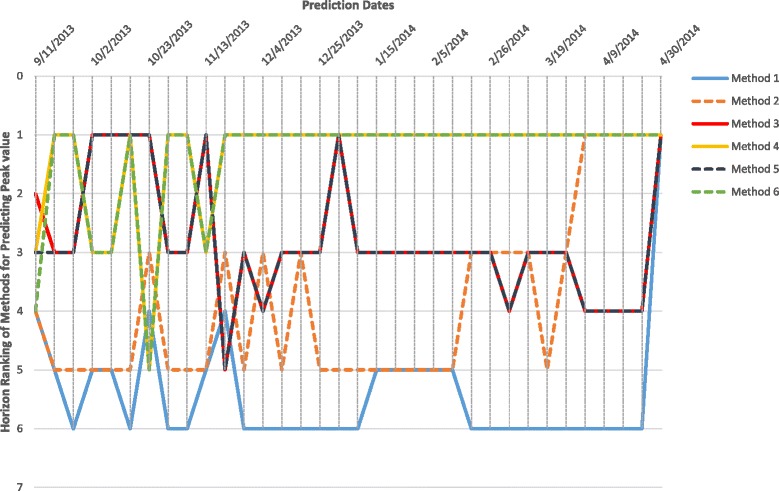
Horizon Ranking of six methods for predicting the peak value calculated based on APE, and sAPE, on Region 1

**Fig. 13:**
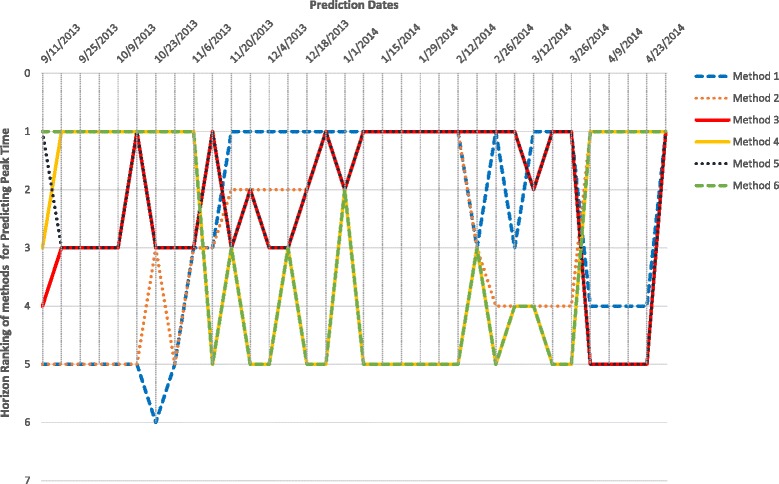
Horizon Ranking of six methods for predicting the peak time calculated based on APE, and sAPE, on Region 1. Methods 4 and 6 are the dominant for the first eight weeks of prediction, and then method 1 wins the first place for seven weeks. In the next eight weeks, methods 1, 3, and 5 are superiors simultaneously

Horizon Ranking helps track the change in accuracy and ranking of the methods over prediction time. Higher fluctuations in the Horizon Ranking across the time steps, hints at the unsuitability of Consensus Ranking as selection criteria for the best method. It is possible that the method that performs best during early stages of prediction may not perform the best at later time-points. Figure 12 shows the evolution of Horizon Ranking of the six methods for predicting the peak value calculated based on APE and sAPE. As shown in Fig. 7, Methods 4 and 6 have the best average Consensus Ranking in predicting peak value and is consistent with observations on Horizon Ranking. In Fig. 12 the ranking of Methods 4 and 6 demonstrates a little fluctuation at the first time-steps. However, as prediction time moves forward these methods provide more accurate forecasts causing them to rank higher. The most interesting case for Horizon Rankings concerns the prediction of peak time. The Consensus Ranking in Fig. 7 selects Method 5 as superior in predicting peak time and Methods 6 and 4 as the second and third best approaches. However, by observing the trends of ranks over prediction times (Fig. 13), Methods 4 and 6 are dominant for the first eight weeks of prediction, then Method 1 wins the first place for seven weeks. In the next eight weeks, Methods 1, 3, and 5 are superiors simultaneously.

**Fig. 14:**
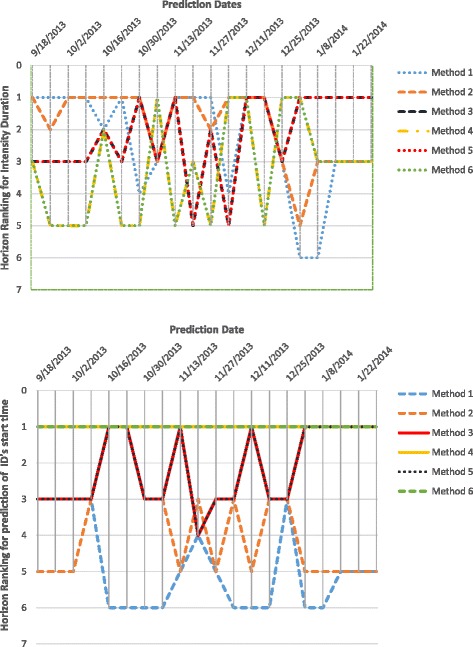
Horizon Ranking of six methods for predicting the Intensity Duration length and start time calculated based on APE, and sAPE, on Region 1

**Fig. 15:**
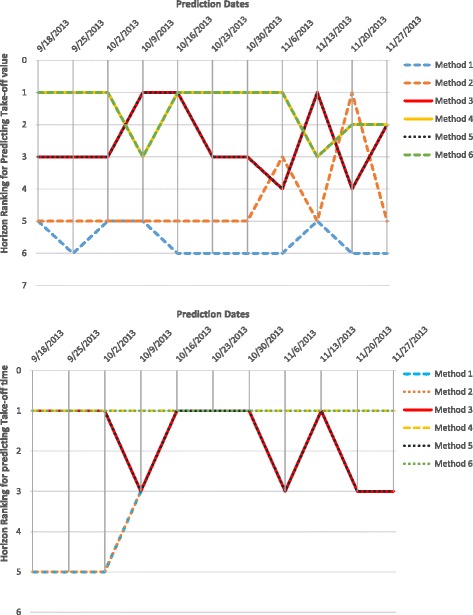
Horizon Ranking of six methods for predicting the Take-off value and time calculated based on APE, and sAPE, on Region 1

**Fig. 16:**
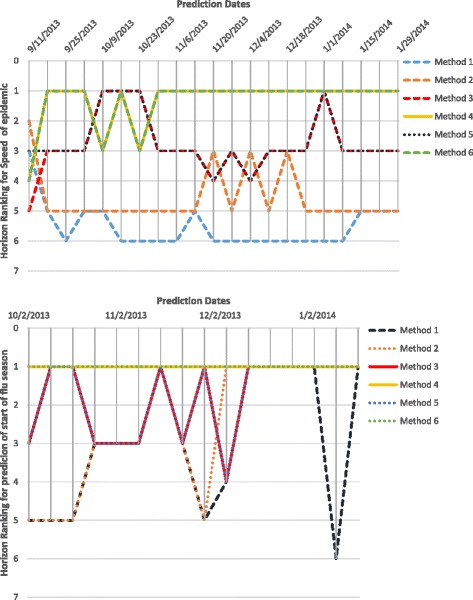
Horizon Ranking graphs for leveraging forecasting methods in predicting Speed of Epidemic and Start of flu season, on Region 1

Figures 14, 15 and 16 show Horizon Ranking graphs for leveraging forecasting methods in predicting other Epi-features. These Horizon Rankings are almost consistent with their corresponding Consensus Rankings which confirms the best methods from the Consensus Ranking perspective could be used for any prediction time.

### Visual comparison of forecasting methods

**Fig. 17:**
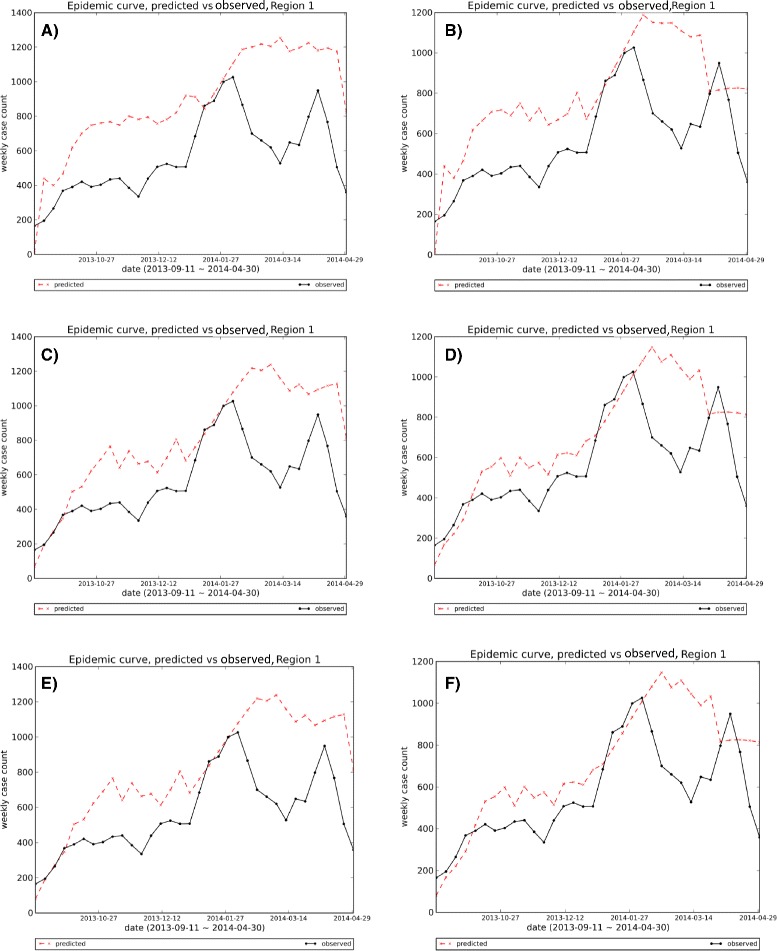
Visual comparison of 1-step-ahead predicted curves generated by six methods vs. the observed curve, Region 1: The first and second methods show bigger deviations from observed curve, especially in the first half of the season. As the six methods are different configurations of one algorithm, their outputs are so competitive and sometimes similar to each other; methods 3 and 5, and methods 4 and 6 show some similarity in their one-step-ahead epidemic curve that is consistent with Horizon Ranking charts for various Epi-features

In order to visualize the output of forecasting methods, we generate the one-step-ahead epidemic curve. Given the early time-series up to time *k* (*y*(1),…,*y*(*k*)) as observed data, the forecasting algorithm predicts the next data point of time-series *x*(*k*+1) and this process is repeated for all values of prediction time *k* where *t*_*b*_≤*k*≤*t*_*e*_. By putting together the short-term predictions, we construct a time-series from *t*_*b*_ to *t*_*e*_ as a one-step-ahead predicted epidemic curve. Figure 17 depicts the one-step-ahead predicted epidemic-curves for HHS region 1 that are generated by the six forecasting methods (refer to Additional files 13, 14, 15, 16, 17, 18, 19, 20, and 21 for other Regions). We used *t*_*b*_=2 and *t*_*e*_=*T*−1 as the beginning and end for the prediction time. As can be seen in Fig. 17, the first and second methods show bigger deviations from the observed curve, especially in the first half of the season. As these six methods are different configurations of one algorithm, their outputs are competitive and sometimes similar to each other. Methods 3 and 5, and Methods 4 and 6 show some similarity in their one-step-ahead epidemic curve that is consistent with Horizon Ranking charts for various Epi-features. However, Horizon Ranking graphs contain more information regarding long-term predictions; therefore, the ranking methods, especially Horizon Ranking, could help experts to distinguish better methods when the outputs of forecasting methods are competitive and judgment based on the visual graph is not straightforward.

## Epidemic forecast evaluation framework

We have proposed a set of Epi-features and error measures and have shown how to evaluate different forecasting methods. These are incorporated into the Software Framework as described (Fig. 1). The software framework, named Epi-Evaluator, receives the observed and predicted epidemic curves as inputs and can generate various rankings based on the choice of Epi-features and error measures. The system is designed as a collection of scripts that are loosely coupled through the data they exchange. This is motivated by two possible scenarios: (a) individuals must be able to use each module in isolation and (b) users must not be restricted to the possibilities described in this paper, and be able to contribute features and measures of their interest.

We also include a standardized visualization module capable of producing a variety of plots and charts summarizing the intermediate outputs of each module. This provides a *plug-and-play* advantage for end users. We envision the end-users ranging from (a) epidemiologists who wish to quickly extract/plot key Epi-features from a given surveillance curve, (b) computational modelers who wish to quantify their predictions and possibly choose between different modeling approaches, (c) forecasting challenge organizers who wish to compare and rank the competing models, and (d) policymakers who wish to decide on models based on their Epi-feature of interest.

## Evaluating stochastic forecasts

The aforementioned measures deal primarily with deterministic forecasts. A number of stochastic forecasting algorithms with some levels of uncertainty have been studied in the literature. Moreover, the observed data may be stochastic because of possible errors in measurements and sources of information. We extend our measures and provide new methods to handle stochastic forecasts and observations. Stochastic forecasts could be in one of the following formats:

- Multiple replicates of the time-series
- A time-series of mean and variance of the predicted values

### Stochastic forecasts as multiple replicates

Most of the stochastic algorithms generate multiple replicates of series and/or state vectors to generate the posterior density function by aggregating discrete values together. A state vector contains the parameters that are used by the epidemic model to generate the epidemic curve (time-series of new infected cases). Therefore, the best state vectors (models) are those that generate an epidemic-curve closer to the observed one (i.e., models with higher likelihood). When the forecasting method’s output is a collection of replicates of state vectors and time-series, we have the option to calculate Epi-features on each series, for each prediction time, and assess the error measures on each series. The error measures can be accumulated across the series through getting Arithmetic Mean, Median, Geometric Mean, etc. to provide a unique comparable value per each method. Table 8 provides advanced error measures to aggregating the error values over the series.

**Table 8:** List of advanced error measures to aggregating the error values across multiple series

Measure name	Formula	Description
Absolute Percentage Error (*A* *P* *E*_*t*,*s*_)	$ APE_{t,s}=|\frac {y_{t} - x_{t,s}}{y_{t}}| $	where *t* is time horizon and *s* is the series index.
Mean Absolute Percentage Error (*M* *A* *P* *E*_*t*_)	$ MAPE=\frac {1}{S} \sum _{s=1}^{S} APE_{t,s} $	where *t* is time horizon, *s* is the series index *S* is the number of series for the method.
Median Absolute Percentage Error (*M* *d* *A* *P* *E*_*t*_)	Median Observation of *A* *P* *E*_*s*_	Obtaining median of APE errors over series.
Relative Absolute Error (*R* *A* *E*_*t*,*s*_)	$ RAE_{t,s}=\frac {|y_{t} - x_{t,s}|}{|y_{t} - x_{RW_{t,s}}|} $	Measures the ratio of absolute error to Random walk error in time horizon t.
Geometric Mean Relative Absolute Error (*G* *M* *R* *A* *E*_*t*_)	$ GMRAE_{t}= [\prod _{s=1}^{S} |RAE_{t,s}| ]^{1/S} $	Measures the Geometric average ratio of absolute error to Random walk error
Median Relative Absolute Error (*M* *d* *R* *A* *E*_*t*_)	Median Observation of *R* *A* *E*_*s*_	Measures the median observation of *R* *A* *E*_*s*_ for time horizon t
Cumulative Relative Error (*C* *u* *m* *R* *A* *E*_*s*_)	$ CumRAE_{s} =\frac {\sum _{t=1}^{T} |y_{t,s} - x_{t,s}|}{\sum _{t=1}^{T}|y_{t,s} - x_{RW_{t,s}}|} $	Ratio of accumulation of errors to cumulative error of Random walk Method
Geometric Mean Cumulative Relative Error (*GMCumRAE*)	$ GMCumRAE =[\prod _{s=1}^{S} |CumRAE_{s}| ]^{1/S} $	Geometric Mean of Cumulative Relative Error across all series.
Median Cumulative Relative Error (*MdCumRAE*)	*M* *d* *C* *u* *m* *R* *A* *E*=*M* *e* *d* *i* *a* *n*(|*C* *u* *m* *R* *A* *E*_*s*_|)	Median of Cumulative Relative Error across all series.
Root Mean Squared Error (*R* *M* *S* *E*_*t*_)	$ RMSE_{t}= \sqrt {\frac {\sum _{s=1}^{S} (y_{t} - x_{t,s})^{2}}{S}} $	Square root of average squared error across series in time horizon t
Percent Better (*P* *B*_*t*_)	$ PB_{t}=\frac {1}{S} \sum _{s=1}^{S} [I\{e_{s,t},e_{WRt}\}] $	Demonstrates average number of times that method overcomes the Random Walk method in time horizon t.
	|*e*_*s*,*t*_|≤|*e*_*WRt*_|⇔*I*{*e*_*s*,*t*_,*e*_*WRt*_}=1	

Armstrong [38] performed an evaluation over some of these measures and suggested the best ones in different conditions. In calibration problems, a sensitive error measure is needed to demonstrate the change in parameters in the error measure values. The EMs with good sensitivity are RMSE, MAPE, and GMRAE. He suggested GMRAE because of poor reliability of RMSE and claimed that MAPE is biased towards the low forecasts [38]. As we mention in the “Discussion” section, we believe that MAPE is not biased in favor of the low forecasts and could also be a good metric for calibration (refer to“Discussion” section). Also, GMRAE could drop to zero when the error contains at least one zero, thus lowering its sensitivity to zero too.

For selecting among forecasting methods, Armstrong suggested MdRAE when the output has a small set of series and MdAPE for a moderate number of series. He believes that reliability, protection against outliers, construct validity, and the relationship to decision-making are more important criteria than sensitivity. MdRAE is reliable and has better protection against outliers. MdAPE has a closer relationship to decision making and is protected against outliers [38].

For the stochastic algorithms that generate multiple time-series with uneven weights, it is important to consider the weight of the series in calculating the arithmetic means. As an illustration, instead of calculating MAPE, sMAPE, RMSE, and MdAPE across the time-series, we suggest measuring weighted-MAPE, weighted-sMAPE, weighted-RMSE, and weighted-MdAPE respectively.

### Stochastic forecasts with uncertainty estimates

Sometimes the output of a stochastic forecasting method is in the form of mean value and variance/uncertainty interval for the predicted value.

In statistics theory, the summation of Euclidean distance between the data points and a fixed unknown point in n-dimensional space is minimized in the mean point. Therefore, the mean value is a good representative of other data points. As a result, we can simply calculate the epi-measure on the predicted mean value of an epidemic curve and compare them through error metrics. However, this comparison is not comprehensive enough because the deviation from the average value is not included in the discussion. To handle this kind of evaluation, we divide the problem into two sub-problems:

- A) Deterministic observation and stochastic forecasts with uncertainty estimates
- B) Stochastic observation and stochastic forecasts with uncertainty estimates

### A) Deterministic observation and stochastic forecasts with uncertainty estimates

BlackIn this case, we assume that each forecasting method’s output is a time-series of uncertain estimates of predicted case counts and is reported by the mean value $\overline {x_{t}}$, variance $\sigma _{t}^{2}$ for data point at *t* ^*th*^ week, and the number of samples *N*_*x*_. For simplicity, we eliminate the subscript *t*. Table 9 lists the required notations used in the following sections. Sample size refers to the number of predicted samples from which the mean and variance are obtained. In the best situation, the forecast algorithm could provide with the probability density function (pdf) of each predicted data point denoted by *f*(*x*), unless we assume the pdf is Normal distribution *f*_*x*_∼*N*(*μ*_*x*_,*σ*_*x*_) for the large enough sample size, or t-distribution *f*_*x*_∼*t*(*μ*_*x*_,*v*) if the sample size is low. T-distribution has heavier tails, which means it is more subject to producing values far from the mean. *N*_*x*_≥30 is assumed as a large sample size. *N*_*x*_ is used to calculate the standard deviation of the random variable X, from the standard deviation of its samples: $\sigma _{x}={\sigma }/\sqrt {N_{x}}$. When the sample size is low, the degree of freedom of t-distribution is calculated by *N*_*x*_: *v*=*N*_*x*_−1.

**Table 9:** Notation Table II

Symbol	Definition
*X*	Random variable *X* (or *X*_*t*_) that is the predicted estimate of a data point at one week(*t* ^*th*^ week)
*f*(*x*)|*f*_*x*_	Probability density function (pdf) of random variable *X*
*μ*_*x*_	Mean value for the random variable *X*
$\sigma _{x} = \sigma /{\sqrt {N_{x}}}$	Standard deviation for the random variable *X*
$\overline {x}$	Mean value of the samples belonging to random variable *X*
*σ*	Standard deviation of the samples belonging to random variable *X*
*v*	*v*=*N*_*x*_−1 Degree of freedom of t-distribution
$ \bar {y} $	$\bar {y}=\frac {1}{n} \sum _{t=1}^{n} (y_{t}) $ : the mean for *y* values over n weeks
*S*_*x*_={*s*_*i*_}	where *s*_*i*_ is the sample from distribution *f*_*x*_
$N_{s_{x}} = |S_{x}| $	Number of sample set *S*_*x*_
*Y*	Random variable *Y* (or *Y*_*t*_) that is the estimate of observed value at one week(*t* ^*th*^ week)
*g*(*y*)|*g*_*y*_	Probability density function (pdf) of random variable *Y*
*S*_*y*_={*s*_*j*_}	where *s*_*j*_ is the sample from distribution *g*_*x*_

In order to evaluate the performance of stochastic methods, we suggest performing the Bootstrap sampling from the distribution *f*(*x*) and generate the sample set *S*_*x*_={*s*_*i*_} for each data point of time-series where |*S*_*x*_|>>*N*_*x*_. Note that we do not have access to the instances of the first sample size, so we generate a large enough sample set from its pdf function *f*(*x*). Then, the six selected error measures, MAE, RMSE, MAPE, sMAPE, MdAPE, and MdsAPE, are calculated across the sample set *S*_*x*_ *for each week*. Additional file 2: Table S8 contains the extended formulation of the error measures used for stochastic forecasts. Using the equations in Additional file 2: Table S8 we can estimate different expected/median errors for each week for a stochastic forecasting method. The weekly errors could be aggregated by deriving Mean or Median across the time to calculate the total error measures for each method. The aggregated error measures can be used to calculate the Consensus Ranking for the existing forecasting approaches. Moreover, having the errors for each week, we can depict the Horizon Ranking and evaluate the trend of rankings across the time similar to the graphs for deterministic approaches.

### B) Stochastic observation and stochastic forecasts with uncertainty estimates

There are many sources of errors in measurements and data collections which result in uncertainty for the observation data. This makes evaluation more challenging. We suggest two categories of solutions to deal with this problem:

- a) Calculating the distance between probability density functions
- b) Calculating the proposed error measures between two probability density functions

### B-a) Calculating the distance between probability density functions

BlackAssuming that both predicted and observed data are stochastic, they are represented as the time-series of probability density functions (pdfs). There are many distance functions that can calculate the distance between two pdfs [21]. Three most common distance functions for this application are listed in Table 10.

**Table 10:** Distance functions to measure dissimilarity between probability density functions of stochastic observation and stochastic predicted outputs

Distance function	Formula (continuous form)	Formula (discrete form)
Bhattacharyya	*D*_*B*_(*P*,*Q*)=−*L* *n*(*B* *C*(*P*,*Q*))	*D*_*B*_(*P*,*Q*)=−*L* *n*(*B* *C*(*P*,*Q*))
	, $BC(P,Q)=\int \sqrt {P(x)Q(x)}dx $	$,BC(P,Q)=\sum \sqrt {P(x)Q(x)} $
Hellinger	$ D_{H}=\sqrt { 2\int {(P(x)-Q(x))^{2}}dx} $	$ D_{H}(P,Q)=\sqrt { 2\sum _{k=1}^{d} {(P(x_{k})-Q(x_{k}))^{2}}} $
	$= 2\sqrt {1-\int \sqrt {P(x)Q(x)}dx} $	$ = 2\sqrt {1-\sum _{k=1}^{d} \sqrt {P(x_{k})Q(x_{k})}} $
Jaccard	-	*D*_*Jac*_=1−*S*_*Jac*_
		$ S_{Jac} = \frac {\sum _{k=1}^{d}{P(x_{k})\times Q(x_{k})}}{ \sum _{k=1}^{d}{P(x_{k})^{2}} + \sum _{k=1}^{d}{Q(x_{k})^{2}}- \sum _{k=1}^{d}{P(x_{k}).Q(x_{k})}} $

Bhattacharyya distance function [21] and Hellinger [39] both belong to the squared-chord family, and their continuous forms are available for comparing continuous probability density functions. In special cases, e.g. when the two pdfs follow the Gaussian distribution, these two distance functions can be calculated by the mean and variances of pdfs as follows [40, 41]:

9 $$\begin{array}{@{}rcl@{}}  D_{B}(P,Q) = \frac{1}{4}ln\! \left(\!\frac{1}{4}\! \left(\!\frac{\sigma_{p}^{2}}{\sigma_{q}^{2}} +\frac{\sigma_{q}^{2}}{\sigma_{p}^{2}}+2\! \right) \!\right) \!+ \frac{1}{4}\!\left(\!\frac{(\mu_{p}-\mu_{q})^{2}}{\sigma_{p}^{2} + \sigma_{q}^{2}}\! \right) \end{array} $$

10 $$\begin{array}{@{}rcl@{}}  D_{H}^{2}(P,Q) = 2\! \left(\!1- \sqrt{\frac{2\sigma_{1}.\sigma_{2} }{\sigma^{2}_{1}+\sigma_{2}^{2}}}.exp\!\left(\frac{-(\mu_{1}-\mu_{2})^{2}}{4(\sigma^{2}_{1}+\sigma_{2}^{2})}\right)\! \right) \end{array} $$

However, calculating the Integral may not be straightforward for an arbitrary pdf. Also, Jaccard distance function is in the discrete form. To solve this problem, we suggest Bootstrap sampling from both predicted and observed pdfs and generating the sample set *S*=*S*_*x*_∪*S*_*y*_ where $S_{x}=\left \{s^{x}_{i}|s^{x}_{i}\sim f(x)\right \}$, $S_{y}=\left \{s_{j}^{y}|s_{j}^{y}\sim g(y)\right \}$, and |*S*_*x*_|=|*S*_*y*_|>>*N*_*x*_. Then we calculate the summation for the distance function over all the items that belong to the sample set *S*. As an example for Jaccard distance function:

11 $$\begin{array}{@{}rcl@{}}{}  \! D_{Jac} \,=\, 1-\!\frac{\sum_{k=1}^{|S|}{f(s_{k})\times g(s_{k})}}{ \sum_{k=1}^{|S|}{f(s_{k})^{2}} + \sum_{k=1}^{|S|}{g(s_{k})^{2}}- \sum_{k=1}^{|S|}{f(s_{k})\times g(s_{k})}} \end{array} $$

Jaccard distance function belongs to the inner product class and incorporates both similarity and dissimilarity of two pdfs. Using one of the aforementioned distance functions between the stochastic forecasts and stochastic observation, we can demonstrate Horizon Ranking across time and also aggregate the distance values by getting the mean value over the weeks, and then, calculate the Consensus Ranking. Although these distance functions between the two pdfs seem to be a reasonable metric for comparing the forecast outputs, it ignores some information about the magnitude of error and its ratio to the real value. In other words, any pair of distributions like (P1,Q1) and (P2,Q2) could have the same distance value if : $|\mu _{P_{1}}-\mu _{Q_{1}}| = |\mu _{P_{2}}-\mu _{Q_{2}}|$ and $\sigma _{P_{1}}=\sigma _{P_{2}}$ and $\sigma _{Q_{1}}=\sigma _{Q_{2}}$. Therefore, the distance functions lose the information about the relative magnitude of error to the observed value.

In the ranking process of different forecasting approaches, as the observed data is assumed to be fixed, this issue will not be a concern. The other problem of using distance functions between pdfs arises when some forecasting methods are stochastic and others are deterministic. As the proposed error measures are not compatible with distance functions, we cannot compare them together.

### B-b) Calculating the error measures between two probability density functions

BlackIn order to compare stochastic and deterministic forecasting approaches together, we suggest estimating the same error measures used for deterministic methods. We perform Bootstrap sampling from both predicted and observed pdfs for each data point of time-series and generate two separate sample sets *S*_*x*_ and *S*_*y*_ where $S_{x}=\left \{s^{x}_{i}|s^{x}_{i}\sim f(x)\right \}$, $S_{y}=\left \{s_{j}^{y}|s_{j}^{y}\sim g(y)\right \}$ and |*S*_*x*_|=|*S*_*y*_|>>*N*_*x*_. The six selected error measures, MAE, RMSE, MAPE, sMAPE, MdAPE, and MdsAPE, could be estimated through the equations listed in Additional file 2: Table S9. These measures incorporate the variance of pdfs through the sampling and represent the difference between the predicted and observed densities by weighted expected value of the error across the samples.

## Discussion

As shown in previous sections, none of the forecasting algorithms may outperform the others in predicting all Epi-features. For a given Epi-feature, we recommend using the Consensus Ranking across different error measures. Further, even for a single Epi-feature, the rankings of methods seem to vary as the prediction time varies.

### Horizon Ranking vs Consensus Ranking

How do we decide on the best method when Horizon Ranking and Consensus Ranking lead to different conclusions? The significant difference between Horizon and Consensus Rankings comes from the fact that Consensus Ranking calculates the average (or median) of the errors for a given time step and then sorts them to determine the ranking. This aggregation of errors is not always a disadvantage, because sometimes a slight difference in errors could change the Horizon Ranking level while the Consensus Ranking accumulates the errors for a whole time-series which gives an overall perspective of each method’s performance. If the purpose of evaluation is to select a method as the best predictor for all weeks, Consensus Rankings can be used to guide the method selection. However, if there is a possibility for using different prediction methods at different periods, we suggest identifying a few time intervals in which the Horizon Rankings of the best methods are consistent. Then, in each time interval, the best method based on Horizon Ranking could be selected, or the Consensus Ranking could be calculated for each period by calculating the average errors (error measures) over time steps. The superior method for each time interval is the one with first Consensus Ranking in that period. One of the advantages of Horizon Ranking is to detect and reduce the effect of outliers across time horizons, whereas Consensus Ranking aggregates the errors across time steps that results in a noticeable change in total value of error measures by outliers.

### MAPE vs sMAPE

MAPE and sMAPE have been the two important error measures in assessing forecast errors since 1993. MAPE was used as the primary measure in M2-Competition, and it was replaced by sMAPE in M3-Competition to overcome the disadvantages of MAPE. One of the drawbacks is that MAPE could get a large or undefined value when the observed data point gets close to zero. This is alleviated to some extent by using the average of observed and predicted value in the denominator for sMAPE. The other issue that has been claimed for MAPE in some literature is biasing in favor of small forecasts. Therefore, the critics believe that MAPE leads to a higher penalty for large overestimation rather than any underestimation. sMAPE, as the symmetric version of MAPE, normalized the error value with the mean of predicted and observed data which limits the range of sMAPE error between 0 and 2 for both overestimating and underestimating of the prediction. However, we believe that although the range of sMAPE function is symmetric, it does not provide a uniform scoring of the errors. We believe sMAPE is significantly biased toward large forecasts.

**Fig. 18:**
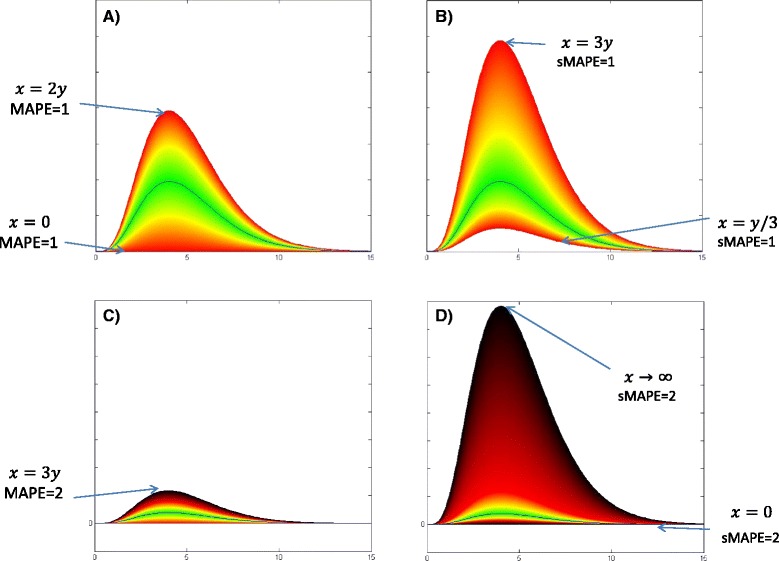
Comparison of MAPE and sMAPE domains and ranges spectrum: Red borders in the left graph (**a**) belong to predicted curves *x*(*t*)=2×*y*(*t*) and *x*(*t*)=0×*y*(*t*) with MAPE = 1 and the red borders in the right chart (**b**) corresponds to *x*(*t*)=3×*y*(*t*) and *x*(*t*)=(1/3)×*y*(*t*) which generate sMAPE = 1. The black borders in graphs **c** & **d** are corresponding to predicted epidemic curves which generates MAPE=2 and sMAPE =2 in the left and right charts sequentially

Figure 18 and Additional file 2: Table S8 demonstrate the corresponding domains that generate equal MAPE or sMAPE errors in term of magnitude. The figures in the left column belong to MAPE and the right ones are sMAPE’s. In Fig. 18, the black line represents the observed epidemic curve (y), and the horizontal axis is the weekly time steps (t). The yellow borders show the predicted curves as overestimated or underestimated predictions which both result in MAPE= 0.5 or sMAPE = 0.5. The green spectrum shows the predicted curves with low values of MAPE or sMAPE. Equal colors in these figures correspond to equal values for the discussed error measure. The red borders in the left graph belong to predicted curves *x*(*t*)=2×*y*(*t*) and *x*(*t*)=0×*y*(*t*) with MAPE = 1 and the red borders in the right chart correspond to *x*(*t*)=3×*y*(*t*) and *x*(*t*)=(1/3)×*y*(*t*) which generate sMAPE = 1. As can be seen, MAPE grows faster than sMAPE which means MAPE reaches 1 with smaller values in the domain. Moreover, MAPE demonstrates symmetrical growth around the observed curve that results in fair scoring toward over and underestimation.

**Fig. 19:**
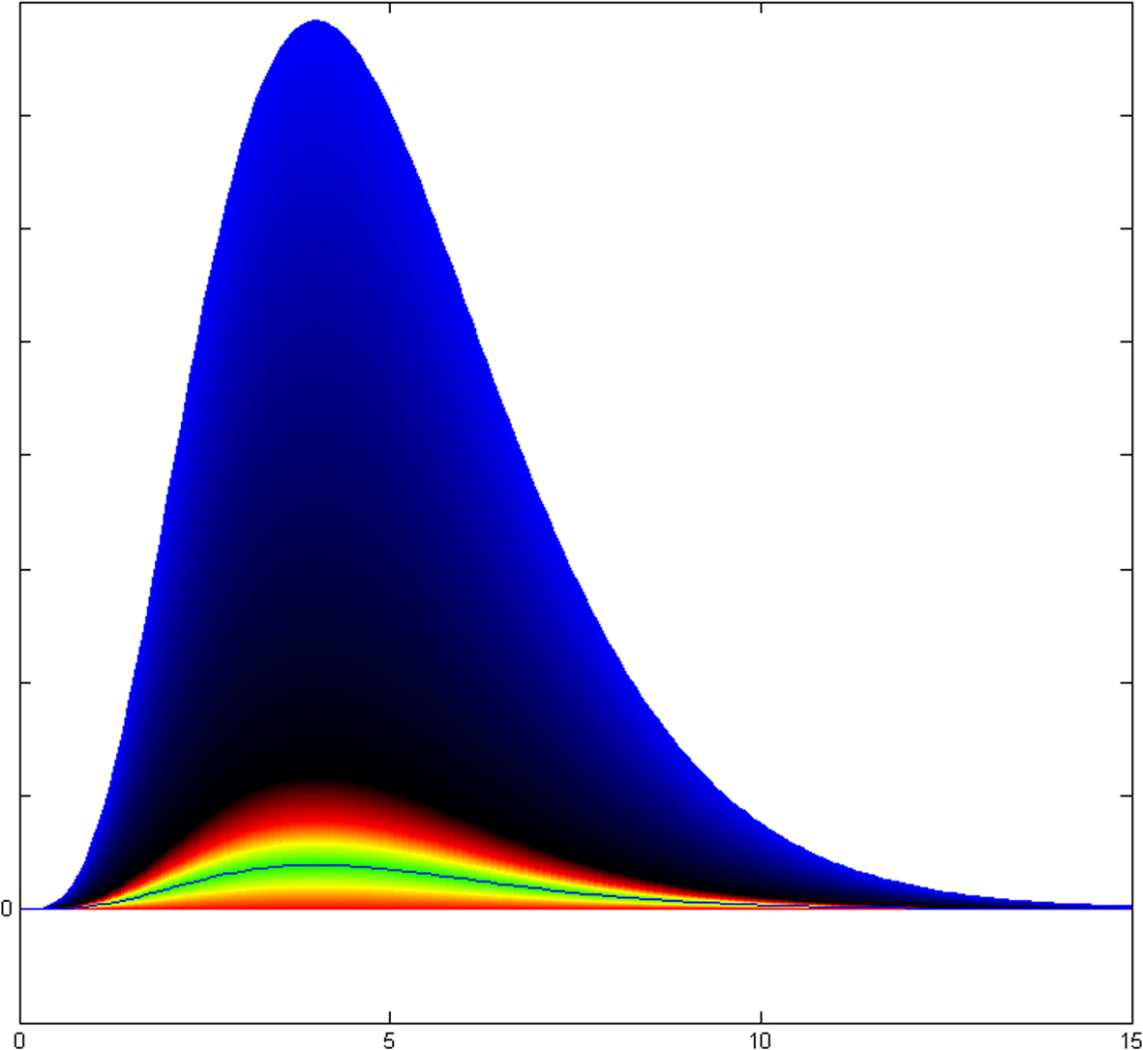
Colored Spectrum of MAPE range: MAPE does not have any limitation from the upper side that results in eliminating the large overestimated forecasting

The black borders in the lower charts of Fig. 18 correspond to the predicted epidemic curve which generates MAPE=2 and sMAPE =2 in the left and right charts sequentially. The color spectrum of sMAPE in the right chart represents the non-symmetric feature of this error measure which is in favor of large predictions. As we couldn’t show the infinity domain for sMAPE, we limited it to the predicted curve *x*(*t*)=20×*y*(*t*). Figure 19 shows the blue spectrum of MAPE that corresponds to large predictions where *x*(*t*)>>3*y*(*t*) and MAPE approaches infinity. This error measure provides more sensible scoring for both calibration and selection problems.

### Relative evaluation vs absolute one

In this paper, we covered how to evaluate the performance of forecasting algorithms relative to each other and rank them based on various error measures. The ranking methods, like the Horizon Ranking, can represent the difference in performances even when the algorithms are so competitive. However, the ranking values conceal the information about error gaps and are senseless when the absolute evaluation of a single method is needed.

The absolute measurement is a challenge because most of the available error measures are not scaled or normalized and do not provide a meaningful range. If one needs to evaluate a single forecasting method, we suggest utilizing of MAPE measure as it is scaled based on the observed value and its magnitude defines how large on average the error is, compared with the observed value.

**Fig. 20:**
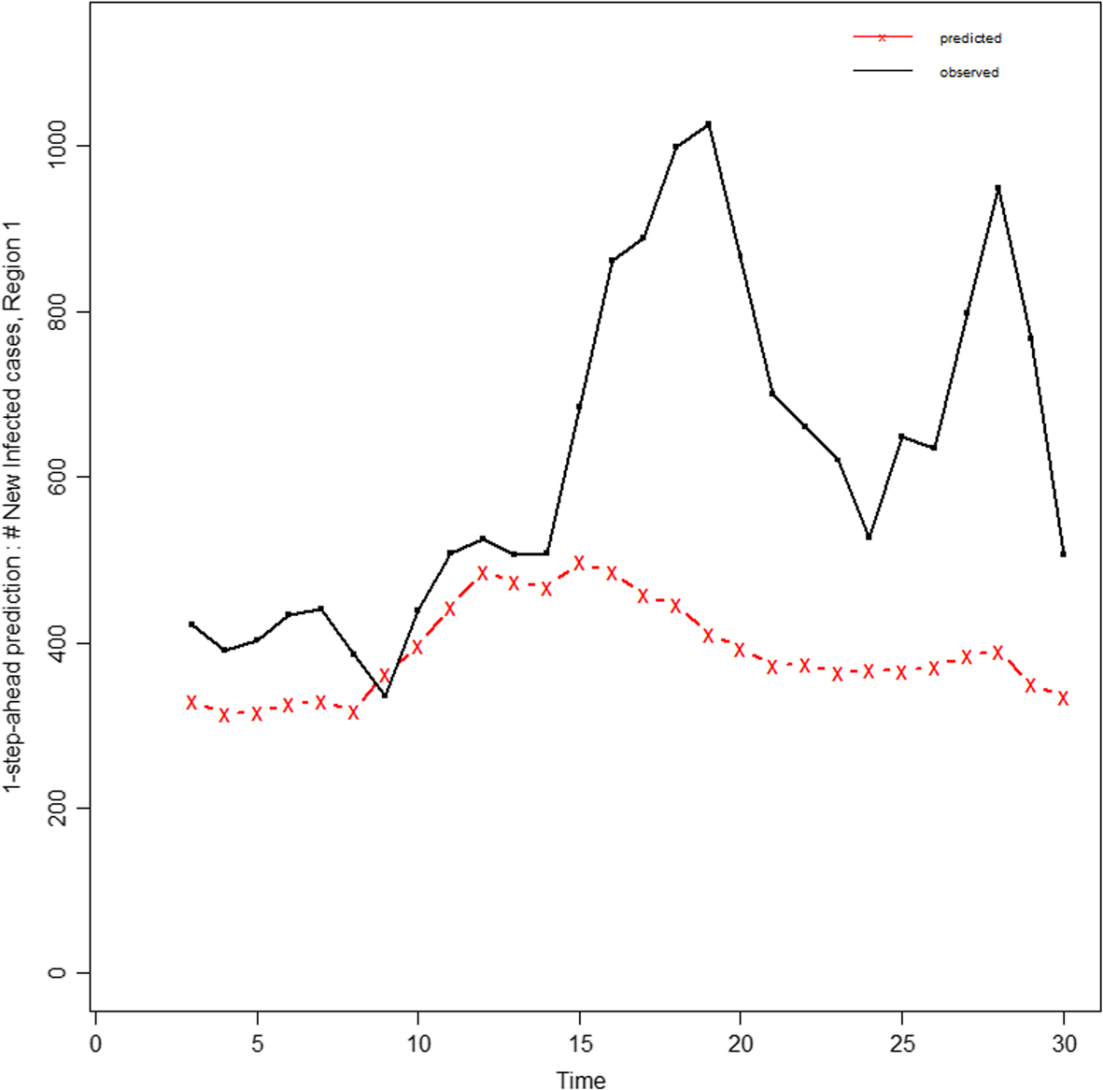
1-step-ahead predicted curve generated by ARIMA vs the observed curve: The large gap between predicted and observed curves shows that ARIMA performance is behind the other six approaches and confirms that clustering approach based on MAPE value could be a good criteria for discriminating methods with totally different performances

For multiple algorithms, we suggest calculating MAPE measure on the one-step-ahead epidemic curve of each algorithm and clustering them based on its MAPE value. As discussed in the previous section and Additional file 2: Table S10, four meaningful intervals for MAPE value could be defined as the criteria to cluster the forecasting approaches into the four corresponding groups: Methods with 0≤*M* *A* *P* *E*≤1/2, Methods with 1/2≤*M* *A* *P* *E*≤1, Methods with 1≤*M* *A* *P* *E*≤2, and Methods with 2≤*M* *A* *P* *E*. This kind of clustering can provide borders between the methods which are completely different in performance. Then, the algorithms of each group can be passed through the three steps of evaluation framework, and be ranked based on various Epi-features and error measures. As an illustration, Table 11 provides the average value of different error measures over all 10 HHS regions for the six aforementioned methods and an autoregressive forecasting method named ARIMA [42]. As can be seen, the MAPE value of the six methods are under 0.5, which clusters all of them in the same category, while the MAPE for the ARIMA method is 0.77 which assigns it to the second group. It means the performance of ARIMA is completely behind all other methods. Figure 20 depicts the one-step-ahead predicted curve of the ARIMA method compared to the observed data that shows the ARIMA output has large deviations from the real observed curve and confirms the correctness of the clustering approach.

**Table 11:** Different error measures calculated for one-step-ahead epidemic curve over whole season (2013-2014), averaged across all HHS regions: Comparing Methods M1 to M6 and ARIMA approach

	MAE	RMSE	MAPE	sMAPE	MdAPE	MdsAPE
Method 1	316.18	378.63	**0.39**	0.33	0.34	0.29
Method 2	293.76	357.34	**0.35**	0.31	0.30	0.26
Method 3	224.53	293.52	**0.25**	0.22	0.22	0.20
Method 4	204.5	274.41	**0.21**	0.21	0.18	0.18
Method 5	224.57	293.90	**0.25**	0.22	0.22	0.20
Method 6	204.25	274.97	**0.21**	0.20	0.18	0.18
ARIMA	1015.60	1187.62	**0.77**	0.74	0.78	0.75

### Prediction error vs calibration error

In this paper, prediction error is considered to calculate the *predicted* error measures, i.e. only the errors after prediction time is taken into account and the deviation between the model curve and data before prediction time is ignored. However, we suggest the evaluator framework in two different modes: forecasting mode vs calibration mode. As mentioned in the forecasting mode, only prediction error is measured. Moreover, if the observed Epi-feature has already occurred in the *i* ^*th*^ week, the forecasts corresponding to the prediction times after the *i* ^*th*^ week are not considered in accumulation of the errors, because they are not interested anymore. However, in calibration mode, the aim is to find the error between model curves and observed data, regardless of the time of observed Epi-feature. Therefore the error measures on one epi-feature are accumulated for all weeks. Also, in calculating error measures on the epidemic curve, the fitting errors before the prediction time are cumulated with prediction errors, to measure the calibration error.

## Conclusion

Evaluating epidemic forecasts arising from varied models is inherently challenging due to the wide variety of epidemic features and error measures to choose from. We proposed different Epi-features for quantifying the prediction accuracy of forecasting methods and demonstrated how suitable error measures could be applied to those Epi-features to evaluate the accuracy and error of prediction. We have applied the proposed Epi-features and error measures on the output of six forecasting methods to assess their performance. As the experimental results showed, different error measures provide various measurements of the error for a single Epi-feature. Therefore, we provided the Consensus Ranking method to aggregate the rankings across error measures and summarize the performance of forecasting algorithms in predicting a single Epi-feature. Based on the first round of rankings, none of the forecasting algorithms could outperform the others in predicting all Epi-features. Therefore, we recommended the second set of rankings to accumulate the analysis for various Epi-features and provide a total summary of the forecasting method capabilities. We also proposed Horizon Ranking to trace the performance of algorithms across the time steps to provide better perspective over time. We finally hint at how these methods can be adapted for the stochastic setting. Choosing the best forecasting method enables policy planners to make more reliable recommendations. Understanding the practical relevance of various Epi-features of interest, and the properties offered by different error measures, will help guide the method selection. We hope that our work allows for a more informed conversation and decision process while using and evaluating epidemic forecasts.

## Additional files

Additional file 1 This is a pdf file in which our forecasting algorithm and the six used configurations are described. (PDF 222 kb)

Additional file 2 This is a pdf file which contains 8 tables in support of the Figs. 7, 18 and 19. (PDF 129 kb)

Additional file 3 Summary of Methodology: This figure is referred in Additional file 1, describing the forecasting pipeline. (PDF 170 kb)

Additional file 4 Consensus Ranking of forecasting methods over all error measures for predicting different Epi-features for Region 2. (PDF 289 kb)

Additional file 5 Consensus Ranking of forecasting methods over all error measures for predicting different Epi-features for Region 3. (PDF 286 kb)

Additional file 6 Consensus Ranking of forecasting methods over all error measures for predicting different Epi-features for Region 4. (PDF 281 kb)

Additional file 7 Consensus Ranking of forecasting methods over all error measures for predicting different Epi-features for Region 5. (PDF 288 kb)

Additional file 8 Consensus Ranking of forecasting methods over all error measures for predicting different Epi-features for Region 6. (PDF 256 kb)

Additional file 9 Consensus Ranking of forecasting methods over all error measures for predicting different Epi-features for Region 7. (PDF 245 kb)

Additional file 10 Consensus Ranking of forecasting methods over all error measures for predicting different Epi-features for Region 8. (PDF 283 kb)

Additional file 11 Consensus Ranking of forecasting methods over all error measures for predicting different Epi-features for Region 9. (PDF 281 kb)

Additional file 12 Consensus Ranking of forecasting methods over all error measures for predicting different Epi-features for Region 10. (PDF 215 kb)

Additional file 13 Visual comparison of 1-step-ahead predicted curves generated by six methods vs. the observed curve, Region 2. (PDF 540 kb)

Additional file 14 Visual comparison of 1-step-ahead predicted curves generated by six methods vs. the observed curve, Region 3. (PDF 307 kb)

Additional file 15 Visual comparison of 1-step-ahead predicted curves generated by six methods vs. the observed curve, Region 4. (PDF 305 kb)

Additional file 16 Visual comparison of 1-step-ahead predicted curves generated by six methods vs. the observed curve, Region 5. (PDF 410 kb)

Additional file 17 Visual comparison of 1-step-ahead predicted curves generated by six methods vs. the observed curve, Region 6. (PDF 418 kb)

Additional file 18 Visual comparison of 1-step-ahead predicted curves generated by six methods vs. the observed curve, Region 7. (PDF 425 kb)

Additional file 19 Visual comparison of 1-step-ahead predicted curves generated by six methods vs. the observed curve, Region 8. (PDF 413 kb)

Additional file 20 Visual comparison of 1-step-ahead predicted curves generated by six methods vs. the observed curve, Region 9. (PDF 389 kb)

Additional file 21 Visual comparison of 1-step-ahead predicted curves generated by six methods vs. the observed curve, Region 10. (PDF 514 kb)

## Abbreviations

Age-AR: Age-specific attack rate
APE: Absolute percentage error
ARI: acute-respiratory-illness
CDC: Centers for disease control and prevention
cMAPE: corrected MAPE
CR: Consensus Ranking
CumRAE: Cumulative relative error
DARPA: Defense advanced research projects agency
Epi-features: Epidemic features
GM: Geometric mean
GMRAE: Geometric mean relative absolute error
HHS: Department of Health and Human Services
ID: Intensity duration
ILINet: Influenza-like illness surveillance network
MAAPE: Mean Arctangent absolute percentage error
MAE: Mean absolute error
MAPE: Mean absolute percentage error
MARE: Mean absolute relative error
MASE: Mean absolute scaled error
M-Competitions: Makridakis competitions
Md: Median
MdRAE: Median relative absolute error
NIH: National Institutes of Health
NMSE: Mean normalized mean squared error
NOAA: National oceanic and atmospheric administration
PB: Percent better
pdf: Probability density function
RMAE: Relative measures
RMSE: Root-mean-square-error
sAPE: Symmetric absolute percentage error
SAR: Secondary attack rate
sMAPE: Symmetric mean absolute percentage error
SpE: Speed of epidemic
TAR: Total attack rate
