# Supplementary material for: A framework for evaluating epidemic forecasts
Source: BMC Infect Dis. 2017 May 15;17:345. doi: 10.1186/s12879-017-2365-1 (PMC5433189; doi:10.1186/s12879-017-2365-1)
Supplement: Supplementary file 1 — This is a pdf file in which our forecasting algorithm and the six used configurations are described. (PDF 222 kb) [file 12879_2017_2365_MOESM1_ESM.pdf]

Supporting Information

A) Forecasting Algorithm

In our model, a digital library is initially built from epidemic curves which are simulated or obtained from past epidemics or data-assimilation-based methods. Simulated epidemic curves could be generated by different kinds of epidemiology models like simple ODE models (SEIR) [1, 2], or individual-based models (EpiFast) [3, 4]. In this paper, the digital library contains simulated epidemics curves generated by running the stochastic version of SEIR model[2] with different parameters to keep the variability in the system. The Susceptible-Exposed-Infectious-Removed (SEIR) model is usually represented by a system of differential equations. To take into account the stochastic nature of infectious disease transmission and to facilitate a computer simulation to produce daily (or weekly) case counts, the SEIR model has been extended to a stochastic discrete time SEIR model. In the stochastic SEIR, daily changes of compartment sizes are sampled from binomial distributions with corresponding probability parameters determined by transmission rate, the mean incubation duration, and the mean infectious duration. Each individual is placed in one, and only one, of the following four states at one time step: Susceptible, Exposed, Infectious, and Removed. The details can be found in[2].

We have implemented the stochastic SEIR model in a C++ simulation code which takes initial sizes of the compartments and the aforementioned disease-related parameters and generates the daily number of infections which can be aggregated to weekly case counts. We would like to emphasize that our Epi-Evaluator works for similar time-series prediction data that may be generated by any other method, either a statistical method or an individual based SEIR model like[4, 5].

To generate our predicted epidemic curve, we pre-select 2205 different models, each being a combination of transmission rate, mean incubation duration, and mean infectious duration. The transmission rate ranges from 0.1E-5 to 0.5E-5; the mean incubation duration ranges from 0.9 to 2.9 (days); the mean infectious duration ranges from 3.1 to 5.1 (days). The initial size of the infectious compartment is set depending on the surveillance data from CDC for the specific HHS region. All 2205 models and corresponding epidemic curves are stored in a digital library. In the forecasting process, given a part of the epidemic curve as a time-series from the surveillance data, the algorithm tries to compare it with all the curves stored in the digital library and find the best match. The tail of the best-matched curve could be taken as the predicted epidemic curve, and its corresponding parameters are used to model the outbreak and predict probable future events about the epidemic (S1 Fig).

To compare a surveillance epidemic curve with the library's curves and find the best-matched parameters, we should calculate the distance between the curves. We use the following measure:

$$dist\left(y, \bar{Z}_P\right) = \sqrt{\sum_{j=1}^{t} d^2\left(y\left(j\right), \bar{Z}_{P^*}\left(j\right)\right)} \quad (1)$$

where j is the time step and t indicates the prediction time on which the tail of epidemic curve is predicted; $\bar{Z}_{P^*}$ is the simulation curve in the digital library

Table 1. Definition of different Distance Functions

Jaccard	Euclidean
$d_{Jac} = \frac{\sum_{t=1}^{n}(x_i - y_i)^2}{\sum_{t=1}^{n} x_i^2 + \sum_{t=1}^{n} y_i^2 - \sum_{t=1}^{n} x_i \times y_i}$	$d_{Euc} = \sqrt{\sum_{t=1}^{n} \lvert x_i - y_i \rvert^2}$

generated by running SEIR model with parameters P^*, $y(j)$ is the observed value from surveillance data and d is a distance function which calculates the distance between the data points (See[6] for more details).

The goal of this paper is applying the Epi-features and Error Metrics to the output of the forecasting methods to evaluate their performance and compare them together. To achieve this goal, we consider six different configurations for our algorithm to generate different results and assess them by Epi-features and Error Metrics to find the best configuration.

The applied configurations are as follows:

- Configuration 1: Jaccard distance function & No trimming.
- Configuration 2: Euclidean distance function & No-trimming.
- Configuration 3: Jaccard distance function & Automatic Trimming
- Configuration 4: Euclidean distance function & Automatic Trimming
- Configuration 5: Jaccard distance function & Ad-hoc Trimming
- Configuration 6: Euclidean distance function & Ad-hoc Trimming

To compare a surveillance epidemic curve with the library's curves, the configurations 1, 3, and 5 applies Jaccard distance function as the distance function used in equation 1 while other configurations used Euclidean distance function. Table 1 demonstrates the definition of Jaccard and Euclidean distance functions. The term "Trimming" which is used in some configurations, refers to the fixing library curves to follow the initially observed data. As noted, the comparator module matches each epidemic curve in the digital library with a given observed curve and determines the best match. Ideally, all the epidemic curves in the digital library should have the same base count as that of a given observed epidemic curve. The base count refers to the number of infected case counts in the first week of epidemic (I_0). However, generating a library for every new initial seed would be time-consuming. Therefore, as an alternative, the Comparator module trims the left portion of digital library curves so that initial count of the input curve (observed data) is less than or equal to a section of digital library's epidemic curves against which comparison distances are computed. We call this process automatic trimming which searches for the trimming point which is the closest value to the base count and trims the head of the epidemic curve. However, we figured out that usually, the best trimming point is the 5th week or near that. Therefore, we suggested the ad-hoc trimming strategy in which only five data points are trimmed to skip the search time. The trimming value 5 is achieved experimentally and heuristically and has shown acceptable results.

The combination of different trimming strategies with various distance functions provide a variety of configurations which results in different outputs for forecasting algorithm.

B) Observations/Proofs on the eliminated error measures

As mentioned before, we have selected MAE, RMSE, MAPE, sMAPE, MdAPE and MdsAPE as the error measures for evaluating the Epi-features and ignored others

based on different reasons. Some observations on the eliminated error measures and their relationships with the considered ones are as follow:

- **Observation 1:** The magnitude of Normalized Mean Squared Error (NMSE) between an observed time-series and a predicted one is monotonic of the magnitude of Root Mean Squared Error (RMSE).
  Proof: Normalized Mean Squared Error scales the average squared error by the variance of observed data which is always a fixed number and doesn't change by forecasting errors. As the square root function is monotonically increasing, the NMSE is a monotonic function of RMSE.
- **Observation 2**: The magnitude of Mean Absolute Scaled Error (MASE) between an observed time-series and a predicted one is proportional to the magnitude of Mean Absolute Error (MAE).
  Proof: MASE scales absolute error by the average error of one-step Random walk method that is the average differences of sequential data points of the observed time-series ($\frac{1}{n-1} \times \sum_{i=2}^{n} |y_i - y_{i-1}|$). As the denominator is always fixed for each observed time-series, the MASE is always proportional and monotonic to MAE.

$$MASE = \frac{1}{n} \sum_{t=1}^{n} \left| \frac{e_t}{\frac{1}{n-1} \times \sum_{i=2}^{n} |y_i - y_{i-1}|} \right| \propto \frac{1}{n} \sum_{t=1}^{n} |e_t| = MAE \tag{2}$$

- **Observation 3**: The magnitude of Mean Absolute Relative Error(MARE)and Relative Measures (RelMAE) between Epi-features obtained from the observed and predicted time-series is proportional to the magnitude of Mean Absolute Error (MAE).
  Proof: Mean Absolute Relative Error(MARE) scales the error in each horizon (e_i) with the corresponding error achieved by Random walk method (e_{RWi}). In order to achieve the Epi-features from the Random walk results, Seasonal-Adjusted Random Walk method is a better option which generates the entire seasonal epidemic curve. However, the prediction of strongly Seasonal Random Walk (seasonal random walk without noise) is independent of the prediction time and generates one unique curve for the remainder of the time-series. Therefore, the Epi-features calculated for Strong Seasonal Random Walk is a constant value and independent of the prediction time which means $e_{RWi} = e_{RW} = \alpha$. Even Seasonal Random Walk prediction with noise is also independent of the prediction time and the obtained Epi-features has a fixed mean value with random noise. Consequently, the ranking achieved by Mean Absolute Relative Error(MARE) with relation to strongly seasonal adjusted random walk method is monotonic with the ranking obtained by simple Mean Absolute Error (MAE). The same reasoning could be used to prove the lemma for Relative Measures (RMAE).

$$MARE = \frac{1}{n} \sum_{t=1}^{n} \left| \frac{e_t}{e_{RWt}} \right| = \frac{1}{n} \sum_{t=1}^{n} \left| \frac{e_t}{\alpha} \right| \propto MAE \tag{3}$$

$$RMAE = \frac{MAE}{MAE_{RW}} = \frac{\sum_{t=1}^{n} |e_t|}{\sum_{t=1}^{n} |e_{RWt|}}| = \frac{1}{n \times \alpha} \sum_{t=1}^{n} |e_t| \propto MAE \quad (4)$$

- **Observation 4**: Geometric Mean of the Relative Absolute Error (GMRAE) is proportional to the magnitude of Geometric Mean of Absolute Error (GMAE) Proof: Using the same reasoning discussed in Observation 3 we have:

$$GMRAE = \left(\prod_{i=1}^{n} |\frac{e_t}{e_{RWt}}| \right)^{(1/n)} = \left(\prod_{i=1}^{n} |\frac{e_t}{\alpha}| \right)^{(1/n)} \propto GMAE \quad (5)$$

We have eliminated the Percent Better (PB) from the pool of measures because it has low sensitivity to reveal the effect of change in methods and parameters[7]. MAAPE is the arctangent of Absolute Percentage ratio to solve the problem of division by zero by mapping the undefined infinity values of percentage error to $\Pi/2$ which is not informative and doesn't discriminate the small and large prediction errors from each other.

Author details

References

1. Kuznetsov, Y.A., Piccardi, C.: Bifurcation analysis of periodic SEIR and SIR epidemic models. Journal of Mathematical Biology **32**(2), 109–121 (1994). doi:10.1007/BF00163027
2. Lekone, P.E., Finkenstädt, B.F.: Statistical inference in a stochastic epidemic SEIR model with control intervention: Ebola as a case study. Biometrics **62**(December), 1170–1177 (2006). doi:10.1111/j.1541-0420.2006.00609.x
3. Roche, B., Drake, J.M., Rohani, P.: An agent-based model to study the epidemiological and evolutionary dynamics of Influenza viruses. BMC bioinformatics **12**(1), 87 (2011). doi:10.1186/1471-2105-12-87
4. Bisset, K., Chen, J., Feng, X.: EpiFast: a fast algorithm for large scale realistic epidemic simulations on distributed memory systems. 23rd international conference on Supercomputing, 430–439 (2009). doi:10.1145/1542275.1542336
5. Barrett C, Bisset, K.R., Eubank Stephen G, Feng X, Marathe, M.: EpiSimdemics: An efficient and scalable framework for simulating the spread of infectious disease on large social networks. International Conference for High Performance Computing, Networking, Storage and Analysis (SC08) (November) (2008). doi:10.1145/1413370.1413408
6. Nsoesie, E.O., Beckman, R.J., Shashaani, S., Nagaraj, K.S., Marathe, M.V.: A Simulation Optimization Approach to Epidemic Forecasting. PloS one **8**(6), 67164 (2013). doi:10.1371/journal.pone.0067164
7. Armstrong, B.J.S., Collopy, F.: Error Measures For Generalizing About Forecasting Methods: Empirical Comparisons By J. Scott Armstrong and Fred Collopy Reprinted with permission form. International Journal of Forecasting **8**(1), 69–80 (1992). doi:10.1016/0169-2070(92)90008-W

Figures

Figure 1. Figure S1: Summary of Methodology 1) Modeling infectious disease dynamics and simulating the spread of it. 2) Storing the simulated epidemic curves (time-series) generated by models with different parameters in digital Library 3) Categorizing epidemic curve (time-series). 4) Gathering different surveillance data from various resources. 5) Integrate different data as a surveillance time-series. 6) Compare surveillance time-series with simulated time-series stored in digital library. 7) Forecasting the future trend of epidemic and special events based on the best matched epidemic curve(s) stored in digital library if any found. And the final stage: Change the forecast results based on additional information.

Figure 2. Consensus Ranking over all Epi-features for Region 2.

Figure 3. Consensus Ranking over all Epi-features for Region 3. Methods 1 and 2 could not predict any take-off in this season, while it was occurred in the observed curve. Therefore, we assigned m+1 as their ranking number for predicting take-off where m is the number of methods that are compared together.

Figure 4. Consensus Ranking over all Epi-features for Region 4.

Figure 5. Consensus Ranking over all Epi-features for Region 5.

Figure 6. Consensus Ranking over all Epi-features for Region 6. Region 6 didn't show start-of-flu season which means the ratio of flu-case counts to other diseases were less than the desired threshold.

Figure 7. Consensus Ranking over all Epi-features for Region 7. Region 7 didn't have Intensity-Duration which means the amount of the new case counts never exceeds the desired threshold = 1000.

Figure 8. Consensus Ranking over all Epi-features for Region 8.

Figure 9. Consensus Ranking over all Epi-features for Region 9.

Figure 10. Consensus Ranking over all Epi-features for Region 10. Region 10 didn't have Intensity-Duration and Take-off either.
