# Supplementary material for: A framework for evaluating epidemic forecasts
Source: BMC Infect Dis. 2017 May 15;17:345. doi: 10.1186/s12879-017-2365-1 (PMC5433189; doi:10.1186/s12879-017-2365-1)
Supplement: Supplementary file 2 — This is a pdf file which contains 8 tables in support of the Figs. 7, 18 and 19. (PDF 129 kb) [file 12879_2017_2365_MOESM2_ESM.pdf]

Table 1. Ranking of methods for predicting peak time based on different error measures for Region 1 over whole season (2013-2014).

	MAPE	sMAPE	RMSE	MdAPE	MdsAPE	MAE	Consensus Ranking
Method 1	5	5	5	2	1	5	3.83
Method 2	6	6	6	2	1	6	4.5
Method 3	3	4	4	2	1	3	2.83
Method 4	3	3	3	2	6	3	3.333
Method 5	1	2	1	1	1	1	1.167
Method 6	2	1	2	2	5	2	2.33

Table 2. Ranking of methods for predicting take-off value based on different error measures for Region 1 over whole season (2013-2014).

	MAPE	sMAPE	RMSE	MdAPE	MdsAPE	MAE	Consensus Ranking
Method 1	6	6	6	6	6	6	6
Method 5	5	5	5	5	5	5	5
Method 3	4	4	4	4	3	4	3.83
Method 4	1	1	2	1	1	1	1.167
Method 5	3	3	3	3	3	3	3
Method 6	1	1	1	2	2	2	1.5

Table 3. Ranking of methods for predicting take-off time based on different error measures for Region 1 over whole season (2013-2014).

	MAPE	sMAPE	RMSE	MdAPE	MdsAPE	MAE	Consensus Ranking
Method 1	1	1	1	1	1	1	1
Method 2	2	2	2	2	2	2	2
Method 3	3	3	3	3	3	3	3
Method 4	5	5	5	5	5	5	5
Method 5	4	4	4	4	4	4	4
Method 6	6	6	6	6	6	6	6

Table 4. Ranking of methods for predicting ID's length based on different error measures for Region 1 over whole season (2013-2014).

	MAPE	sMAPE	RMSE	MdAPE	MdsAPE	MAE	Consensus Ranking
Method 1	2	2	2	6	6	2	3.33
Method 2	1	1	1	1	1	1	1
Method 3	3	3	3	4	4	3	3.33
Method 4	5	5	5	2	2	5	4
Method 5	4	4	4	5	5	4	4.33
Method 6	6	6	6	2	2	6	4.67

Table 5. Ranking of methods for predicting ID's start time based on different error measures for Region 1 over whole season (2013-2014).

	MAPE	sMAPE	RMSE	MdAPE	MdsAPE	MAE	Consensus Ranking
Method 1	6	6	6	5	5	6	5.67
Method 2	5	5	5	3	3	5	4.33
Method 3	3	3	3	3	4	3	3.167
Method 4	1	1	1	1	1	1	1
Method 5	4	4	4	6	6	4	4.67
Method 6	2	2	2	2	2	2	2

Table 6. Ranking of methods for predicting Speed of Epidemic based on different Error Measures for Region 1 over whole season (2013-2014).

	MAPE	sMAPE	RMSE	MdAPE	MdsAPE	MAE	Consensus Ranking
Method 1	6	6	6	5	6	6	5.83
Method 2	5	5	5	4	3	5	4.5
Method 3	3	4	3	2	4	3	3.167
Method 4	1	2	1	1	1	1	1.167
Method 5	4	3	4	6	4	4	4.167
Method 6	2	1	2	2	1	2	1.67

Table 7. Ranking of methods for predicting Start of Flu Season based on different Error Measures for Region 1 over whole season (2013-2014).

	MAPE	sMAPE	RMSE	MdAPE	MdsAPE	MAE	Consensus Ranking
Method 1	6	6	6	6	6	6	6
Method 2	5	5	5	5	5	5	5
Method 3	3	3	3	3	3	3	3
Method 4	1	1	1	1	1	1	1
Method 5	3	3	3	3	3	3	3
Method 6	1	1	1	1	1	1	1

Table 8. Error measures to measure error between the deterministic observation and stochastic predicted outputs.

Measure name	Formula	Weighted-Expectation/Median of Measure
Absolute Error (AE)	$AE = \|s_i - y\|$	$E(AE) = \sum_{i=1}^{N_{s_x}} f(s_i) \times \|s_i - y\|$
Absolute Percentage Error (APE)	$APE = \frac{\|s_i - y\|}{\|y\|}$	$E(APE) = \sum_{i=1}^{N_{s_x}} f(s_i) \times \frac{\|s_i - y\|}{y}$
symmetric Absolute Percentage Error (sAPE)	$sAPE = \frac{2\times\|s_i - y\|}{\|s_i + y\|}$	$E(sAPE) = 2 \times \sum_{i=1}^{N_{s_x}} f(s_i) \times \frac{\|s_i - y\|}{s_i + y}$
Squared Error (SE)	$SE = (s_i - y)^2$	$RE(SE) = \sqrt{\sum_{i=1}^{N_{s_x}} f(s_i) \times (s_i - y)^2}$
Absolute Percentage Error (APE)	$APE = \frac{\|s_i - y\|}{\|y\|}$	$Md(APE) = weightedMedian_{i=1}^{N_{s_x}} \left\{ \frac{\|s_i - y\|}{y} \right\}$ where $w_i = f(s_i)$
symmetric Absolute Percentage Error (sAPE)	$sAPE = \frac{2\times\|s_i - y\|}{\|s_i + y\|}$	$Md(sAPE) = weightedMedian_{i=1}^{N_{s_x}} \left\{ 2. \frac{\|s_i - y\|}{s_i + y} \right\}$ where $w_i = f(s_i)$

Table 9. Error measures to measure error between the stochastic observation and stochastic predicted outputs.

Measure name	Formula	Weighted-Expectation/Median of Measure
Absolute Error (AE)	$AE_{i,j} = \lvert s_i^x - s_j^y \rvert$	$E(AE) = \sum_{i=1}^{N_{s_x}} \sum_{j=1}^{N_{s_y}} f(s_i^x).g(s_j^y) \times \lvert s_i^x - s_j^y \rvert$
Absolute Percentage Error (APE)	$APE_{i,j} = \frac{\lvert s_i^x - s_j^y \rvert}{\lvert s_j^y \rvert}$	$E(APE) = \sum_{i=1}^{N_{s_x}} \sum_{j=1}^{N_{s_y}} f(s_i^x).g(s_j^y) \times \frac{\lvert s_i^x - s_j^y \rvert}{\lvert s_j^y \rvert}$
symmetric Absolute Percentage Error (sAPE)	$sAPE_{i,j} = \frac{2 \times \lvert s_i^x - s_j^y \rvert}{\lvert s_i^x + s_j^y \rvert}$	$E(sAPE) = 2 \times \sum_{i=1}^{N_{s_x}} \sum_{j=1}^{N_{s_y}} f(s_i^x).g(s_j^y) \times \frac{\lvert s_i^x - s_j^y \rvert}{s_i^x + s_j^y}$
Squared Error (SE)	$SE_{i,j} = (s_i^x - s_j^y)^2$	$RE(SE) = \sqrt{\sum_{i=1}^{N_{s_x}} \sum_{j=1}^{N_{s_y}} f(s_i^x).g(s_j^y) \times (s_i^x - s_j^y)^2}$
Absolute Percentage Error (APE)	$APE_{i,j} = \frac{\lvert s_i^x - s_j^y \rvert}{\lvert s_j^y \rvert}$	$Md(APE) = weightedMedian_{(i=1,j=1)}^{(N_{s_x},N_{s_x})} \left\{ \frac{\lvert s_i^x - s_j^y \rvert}{\lvert s_j^y \rvert} \right\}$<br>where $w_{i,j} = f(s_i^x).g(s_j^y)$
symmetric Absolute Percentage Error (sAPE)	$sAPE_{i,j} = \frac{2 \times \lvert s_i^x - s_j^y \rvert}{\lvert s_i^x + s_j^y \rvert}$	$Md(sAPE) = weightedMedian_{(i=1,j=1)}^{(N_{s_x},N_{s_x})} \left\{ 2.\frac{\lvert s_i^x - s_j^y \rvert}{\lvert s_i^x + s_j^y \rvert} \right\}$<br>where $w_{i,j} = f(s_i^x).g(s_j^y)$

Table 10. Corresponding domains that generate equal MAPE or sMAPE errors in term of magnitude.

Error Metric	MAPE	MAPE	sMAPE	sMAPE
Curves' Color	Range	Domain	Range	Domain
	e=2	–	e=2	x=0
	e=1	x=0	e=1	$x = \frac{y}{3}$
	e=1/2	$x = \frac{y}{2}$	e=1/2	$x = \frac{3y}{5}$
	e=0	$x = y$	e=0	x=y
	e=1/2	$x = \frac{3y}{2}$	e=2	$x = \frac{5y}{3}$
	e=1	$x = 2y$	e=1/2	$x = 3y$
	e=2	$x = 3y$	e=2	$x \to \infty$
	$e \succ 2$<br>$e \to \infty$	$x \succ 3y$<br>$x \to \infty$	$e \succ 2$<br>$e \succ 2$	–<br>–

Table 11. Other forms of measure error measures.

Error Measure name	Formula	Description
Mean Percentage Error (MPE_t)	$MPE = \frac{1}{n}\sum_{t=1}^{n}[\frac{e_t}{y_t}]$	Similar to MAPE but sign of error demonstrates the direction of error
Signed Mean Squared Error (SMSE)	$SMSE = \frac{1}{n}\sum_{t=1}^{n}[\frac{e_t}{\lvert e_t\rvert}] \times e_t^2$	Similar to MSE but provides the direction of the error
Median Absolute Percentage Error ($MdAPE$)	Median Observation of APE	where Observations are sorted APE_t , t is time horizon.
Root Mean Square Percentage Error ($RMSPE$)	$RMSPE = \sqrt{[\frac{1}{n}\sum_{t=1}^{n}[\frac{e_t}{y_t}]^2]}$	Penalizes large errors.
symmetric Median Absolute Percentage Error ($sMdAPE$)	Median Observation of $sAPE$	where Observations are sorted $sAPE_t$, t is time horizon.
Relative Mean Absolute Error ($RMAE$)	$RMAE = \frac{MAE}{MAE_{RW}}$	Measures the ratio of mean absolute error to Random walk error across time horizons.
Geometric Mean Relative Absolute Error ($GMRAE$)	$GMRAE = [\prod_{t=1}^{N} \lvert RAE_t\rvert]^{1/N}$	Measures the average ratio of relative absolute error to Random walk error
Relative Root Mean Square Error ($RelRMSE$)	$RelRMSE = \frac{RMSE}{RMSE_{RW}}$	Measures the ratio of RMSE to Random walk RMSE across time horizons.
Log Mean Squared Error Ratio, (LMR)	$LMR = log\left(\frac{RMSE}{RMSE_{RW}}\right)$	Measures log form of the ratio of RMSE to Random walk RMSE across time horizons.
Relative Geometric Root Mean Square Error ($RGRMSE$)	$RGRMSE = \frac{GRMSE}{GRMSE_{RW}}$	complex assessment of the relative geometric standard deviation.
