# Supplementary figures and images for: A framework for evaluating epidemic forecasts
Source: BMC Infect Dis. 2017 May 15;17:345. doi: 10.1186/s12879-017-2365-1 (PMC5433189; doi:10.1186/s12879-017-2365-1)

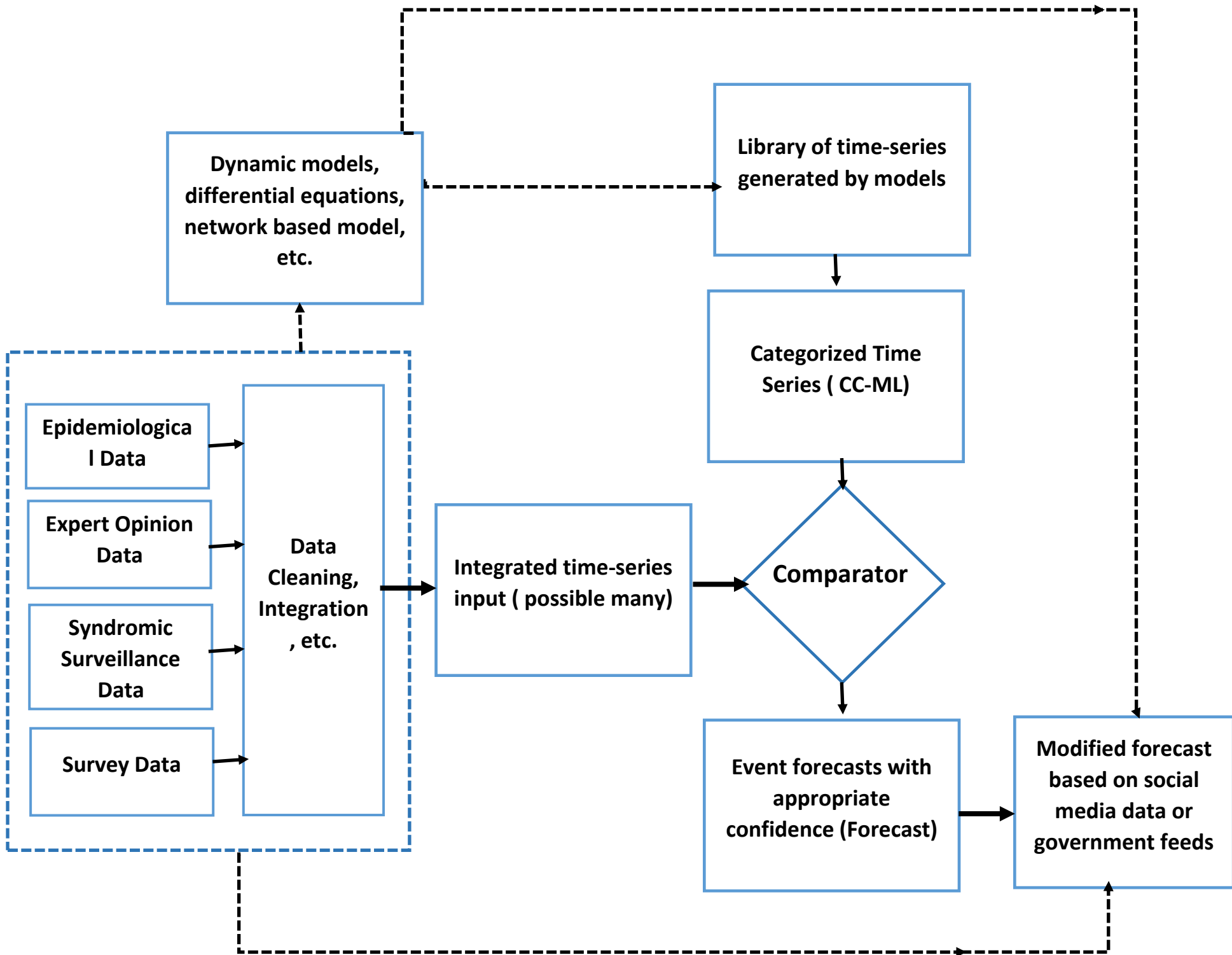

Supplement: Supplementary file 3 — Summary of Methodology: This figure is referred in Additional file 1, describing the forecasting pipeline. (PDF 170 kb) [file 12879_2017_2365_MOESM3_ESM.pdf]

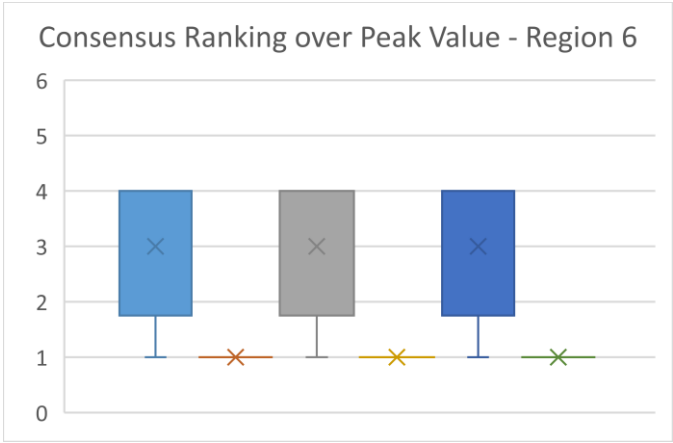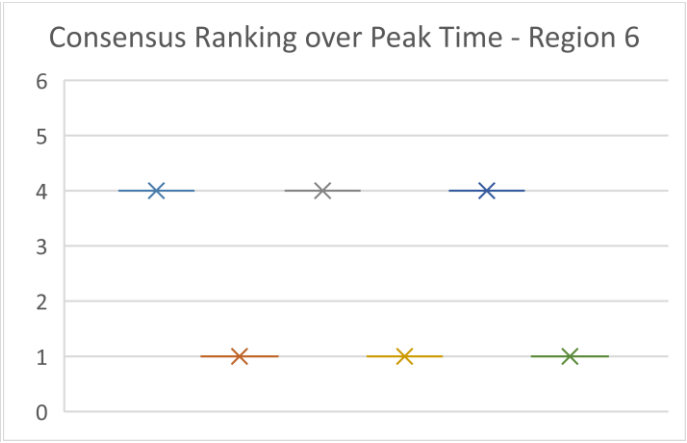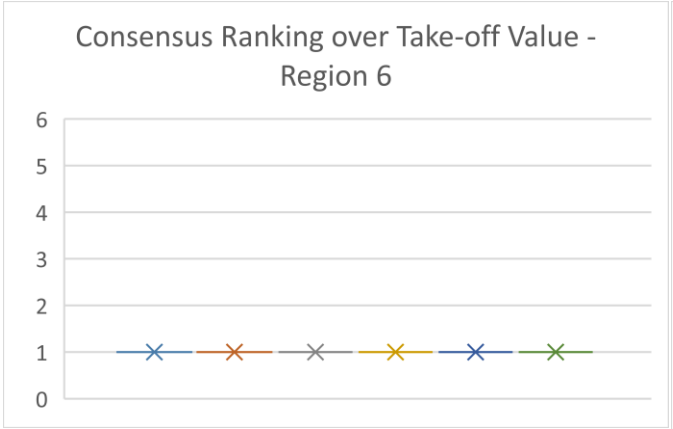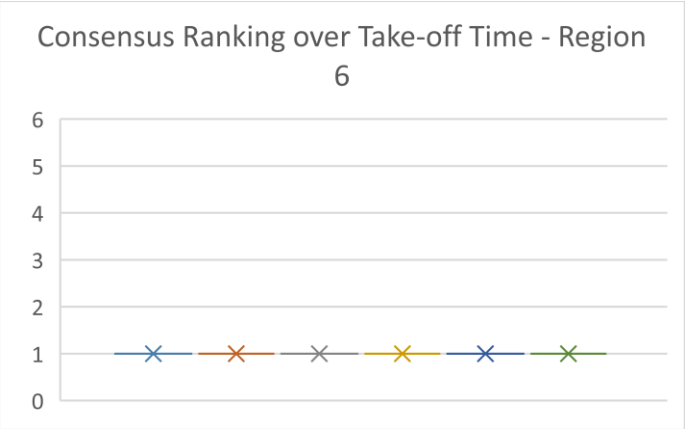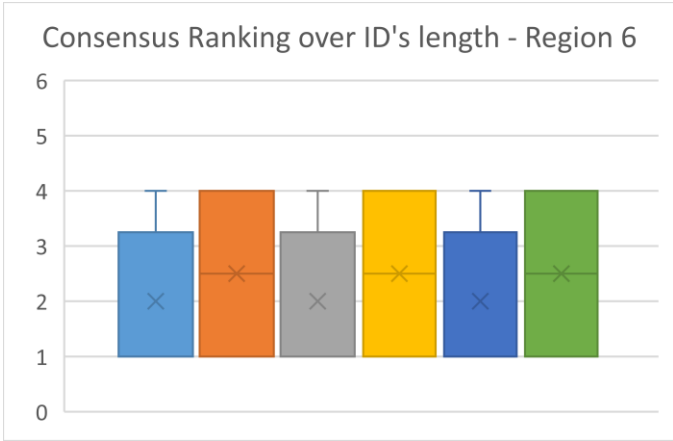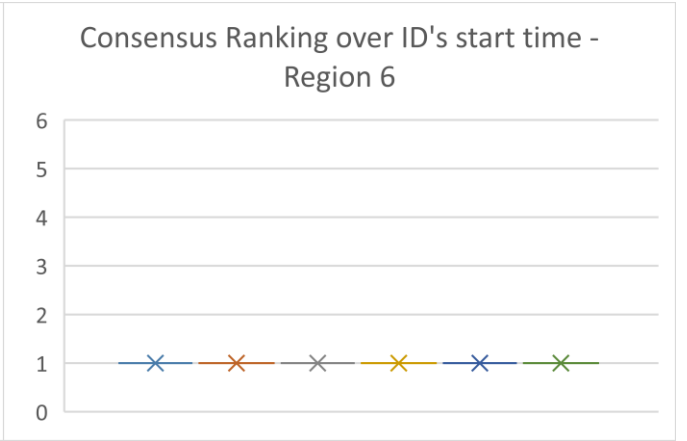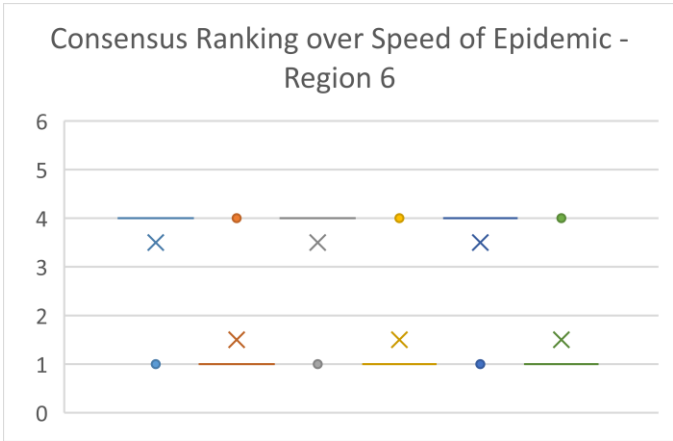

Supplement: Supplementary file 8 — Consensus Ranking of forecasting methods over all error measures for predicting different Epi-features for Region 6. (PDF 256 kb) [file 12879_2017_2365_MOESM8_ESM.pdf]

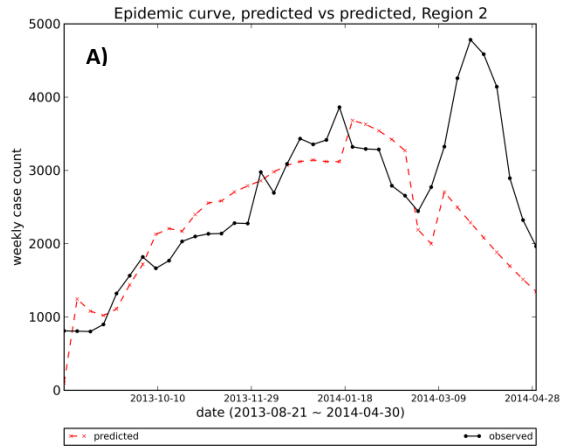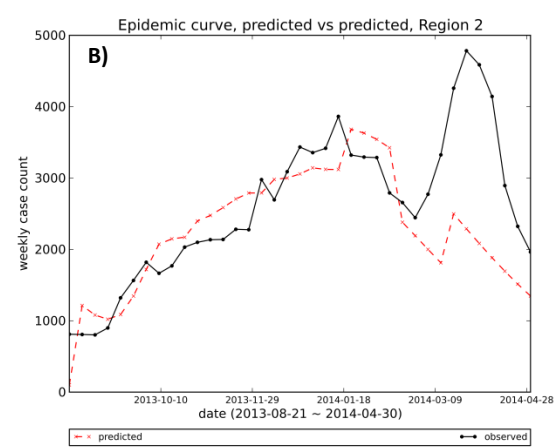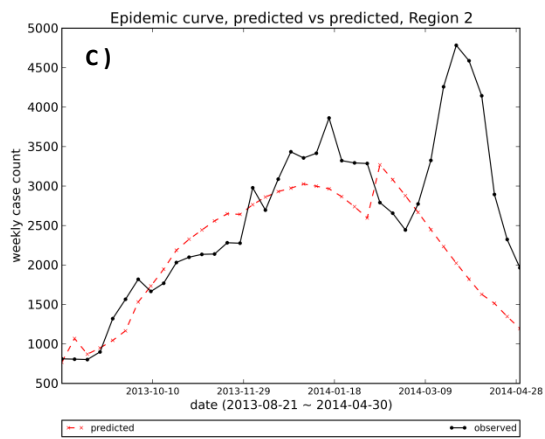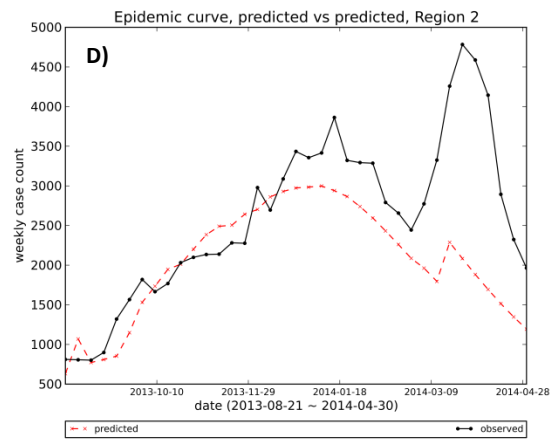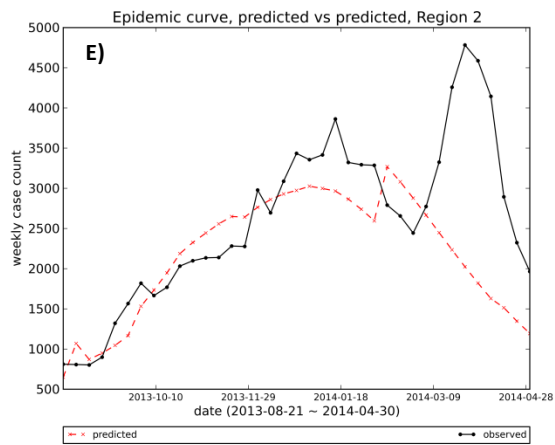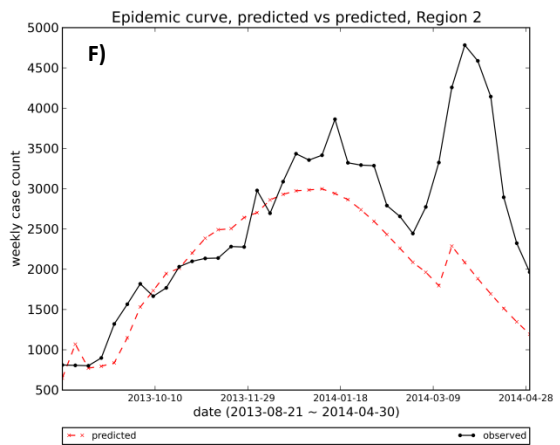

Supplement: Supplementary file 13 — Visual comparison of 1-step-ahead predicted curves generated by six methods vs. the observed curve, Region 2. (PDF 540 kb) [file 12879_2017_2365_MOESM13_ESM.pdf]

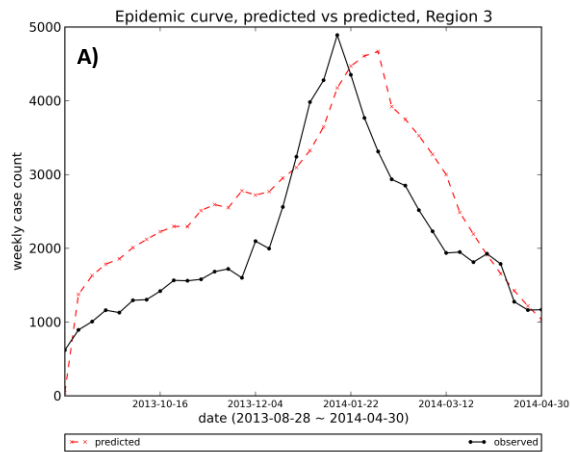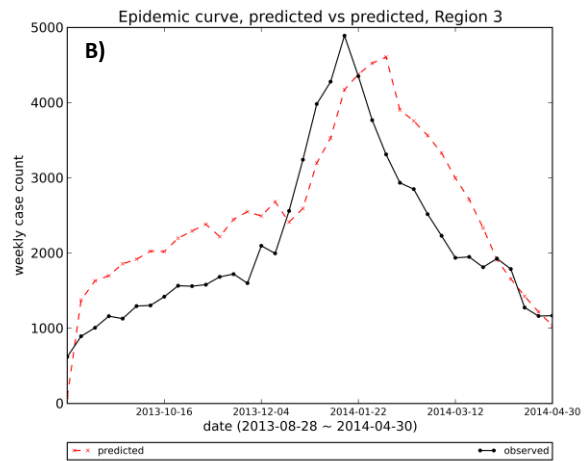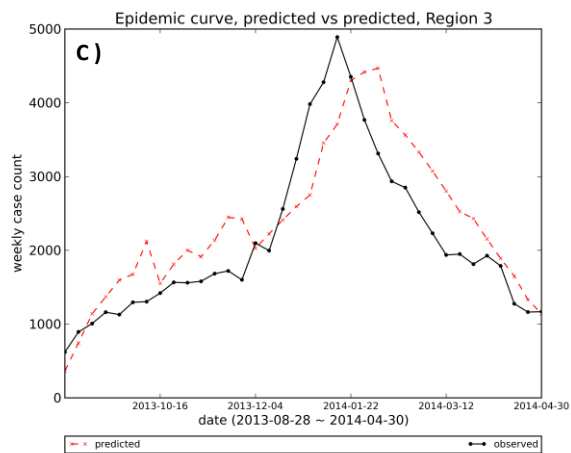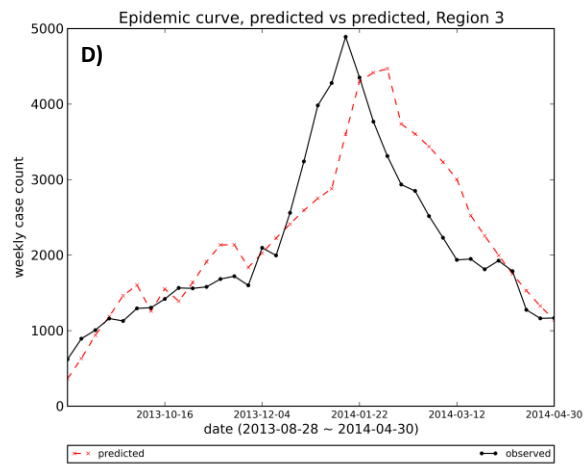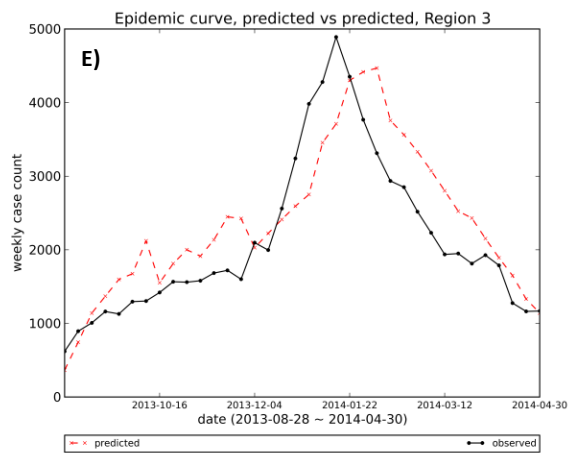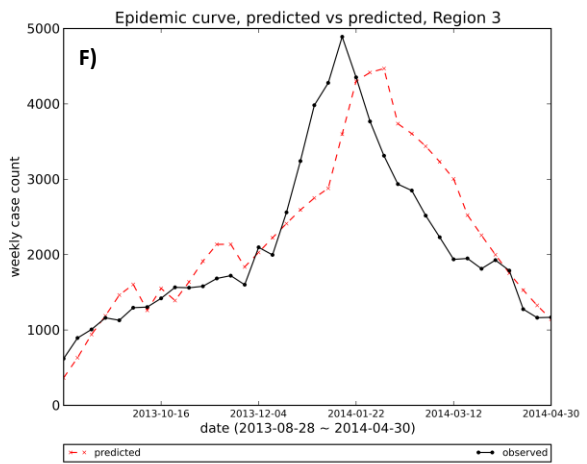

Supplement: Supplementary file 14 — Visual comparison of 1-step-ahead predicted curves generated by six methods vs. the observed curve, Region 3. (PDF 307 kb) [file 12879_2017_2365_MOESM14_ESM.pdf]

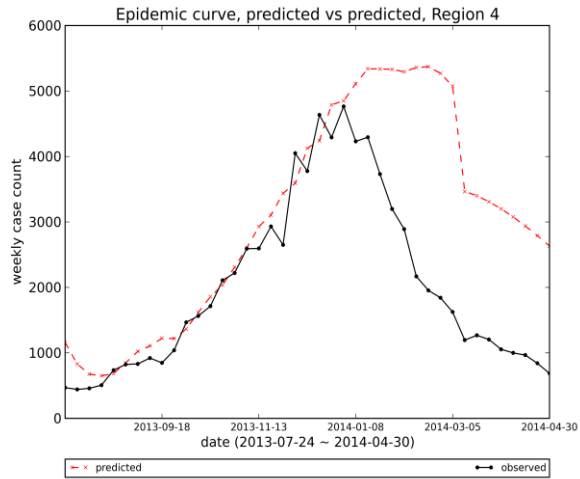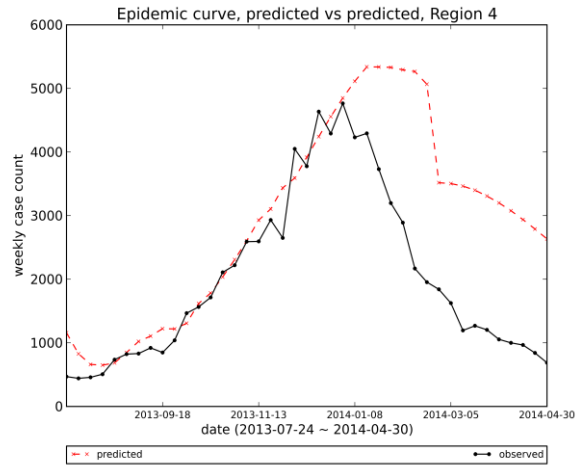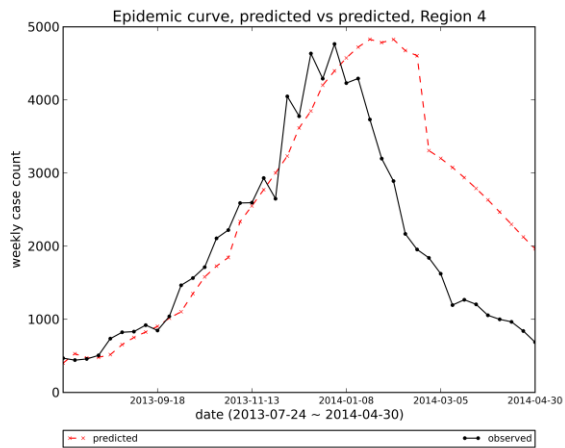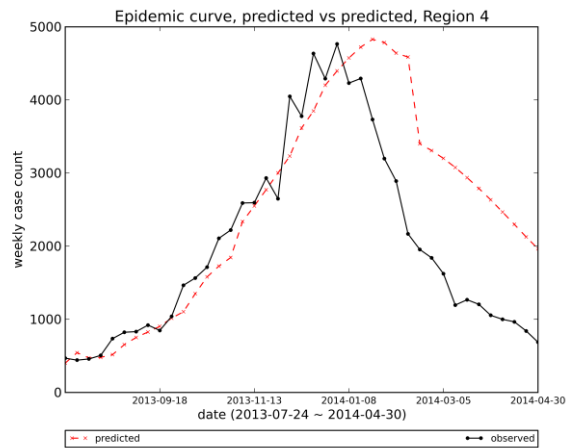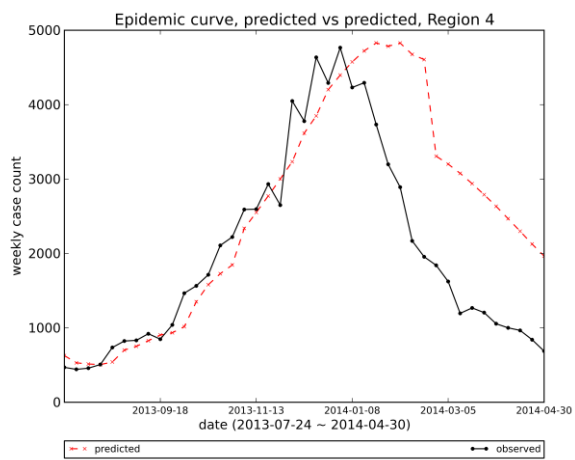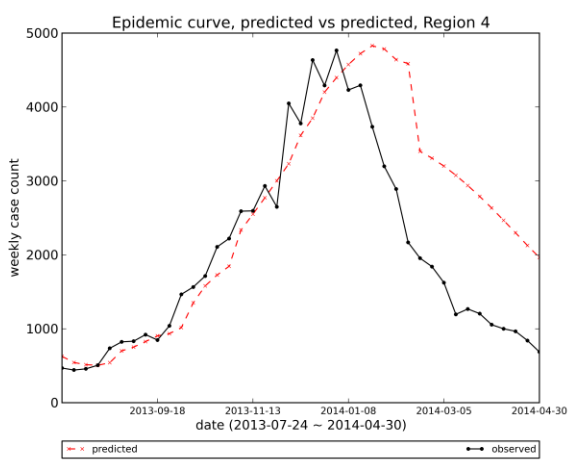

Supplement: Supplementary file 15 — Visual comparison of 1-step-ahead predicted curves generated by six methods vs. the observed curve, Region 4. (PDF 305 kb) [file 12879_2017_2365_MOESM15_ESM.pdf]

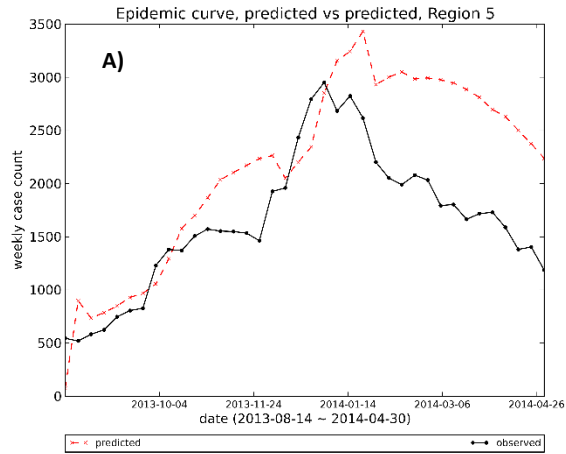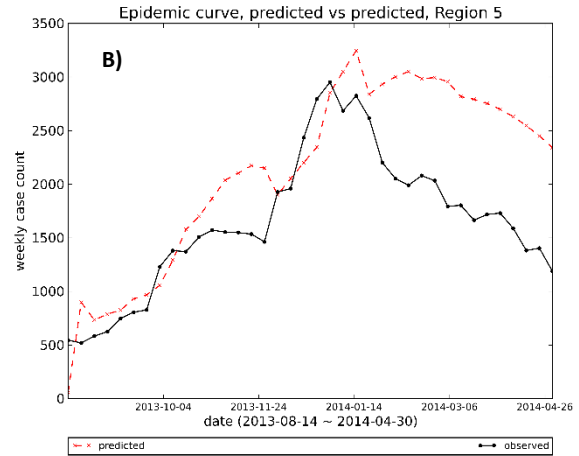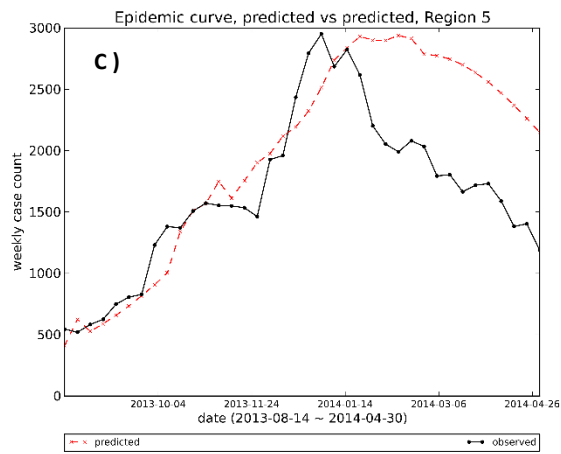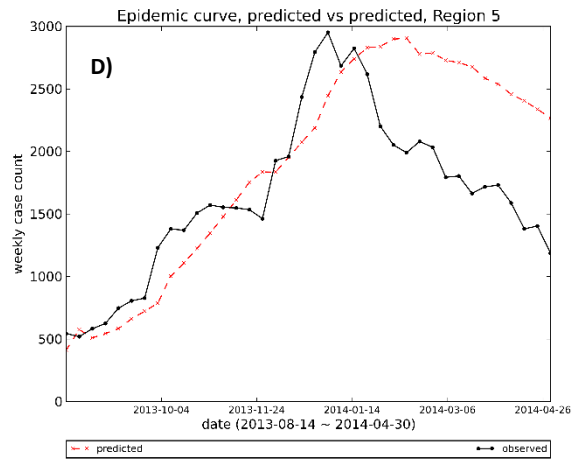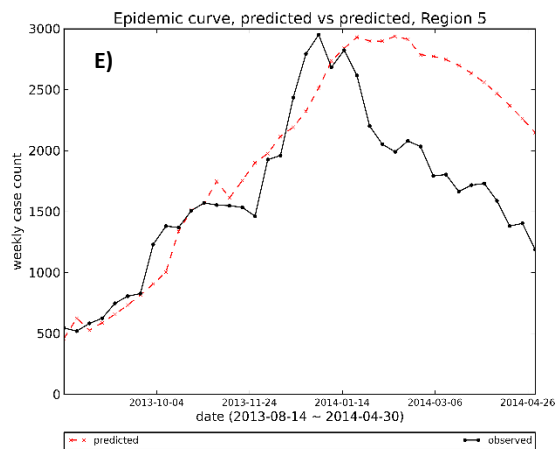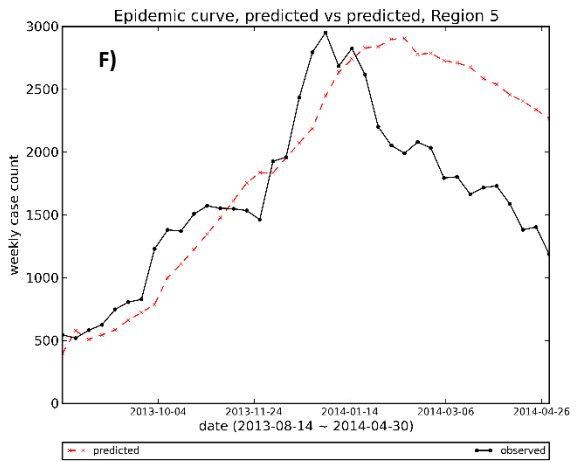

Supplement: Supplementary file 16 — Visual comparison of 1-step-ahead predicted curves generated by six methods vs. the observed curve, Region 5. (PDF 410 kb) [file 12879_2017_2365_MOESM16_ESM.pdf]

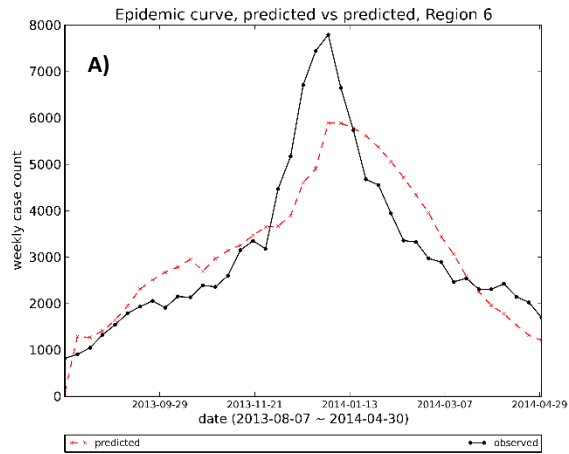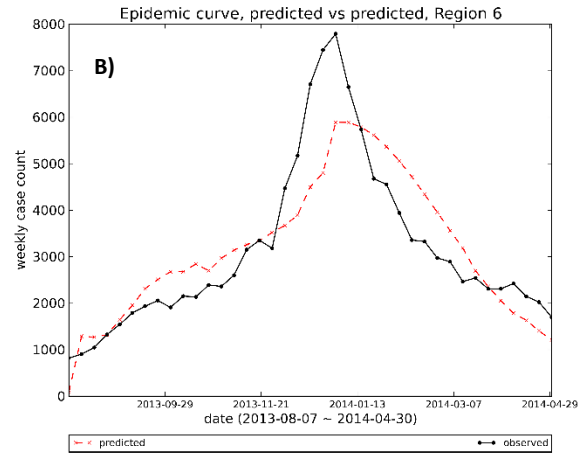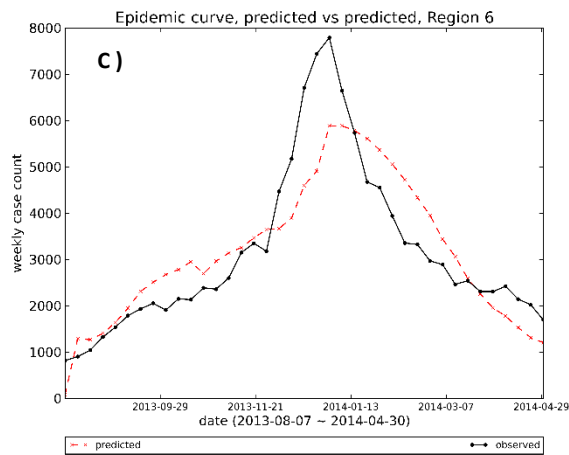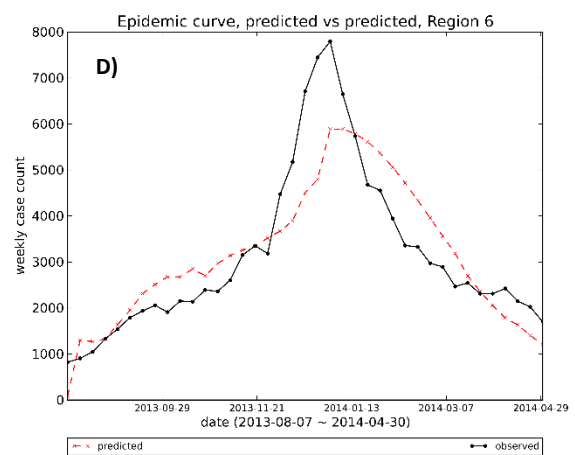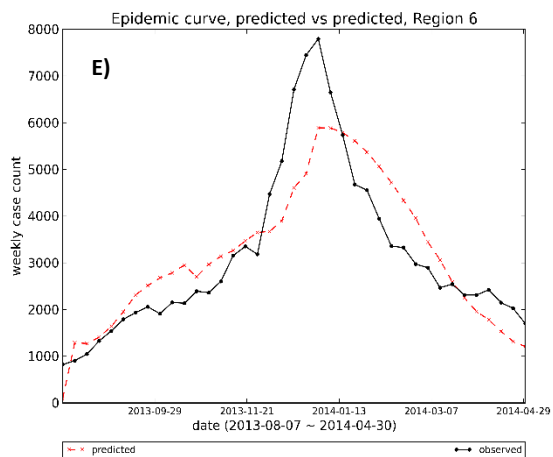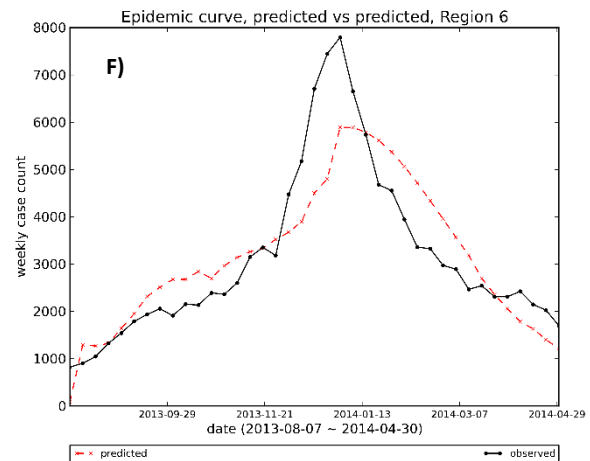

Supplement: Supplementary file 17 — Visual comparison of 1-step-ahead predicted curves generated by six methods vs. the observed curve, Region 6. (PDF 418 kb) [file 12879_2017_2365_MOESM17_ESM.pdf]

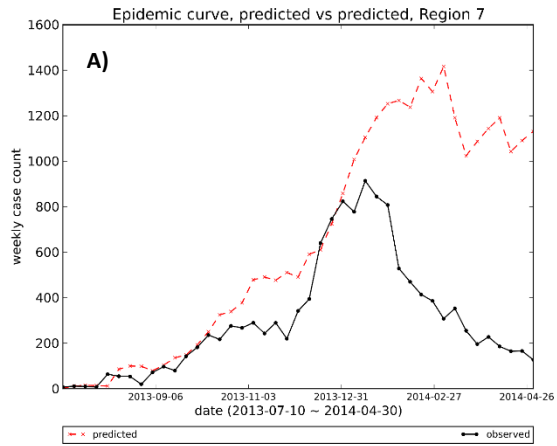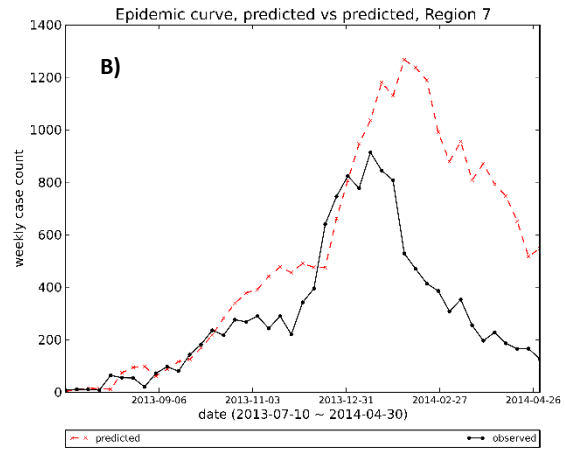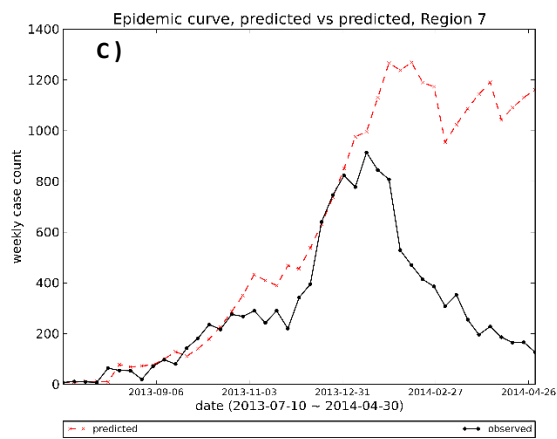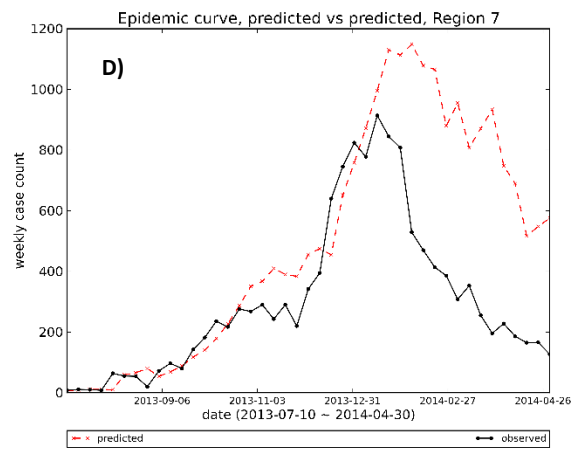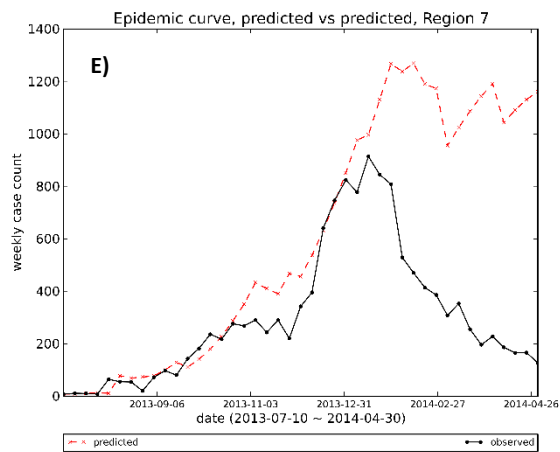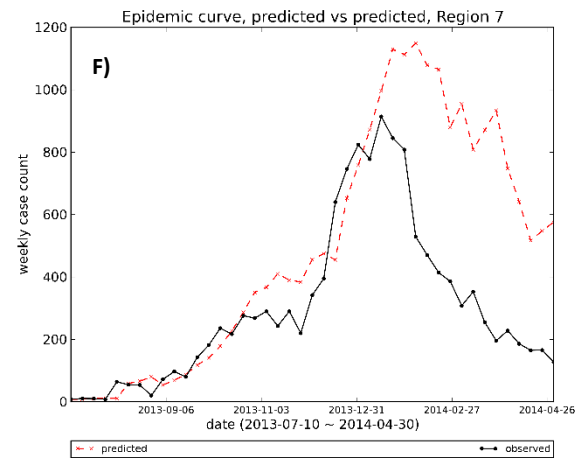

Supplement: Supplementary file 18 — Visual comparison of 1-step-ahead predicted curves generated by six methods vs. the observed curve, Region 7. (PDF 425 kb) [file 12879_2017_2365_MOESM18_ESM.pdf]

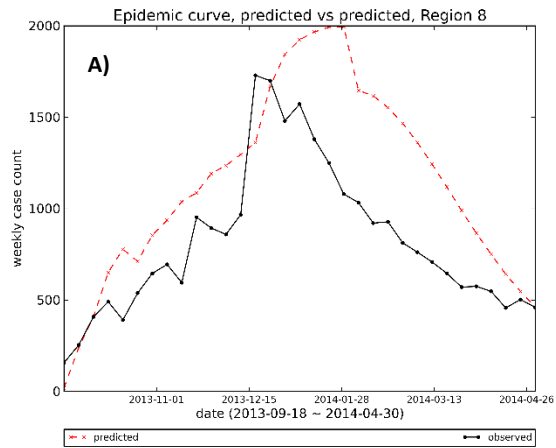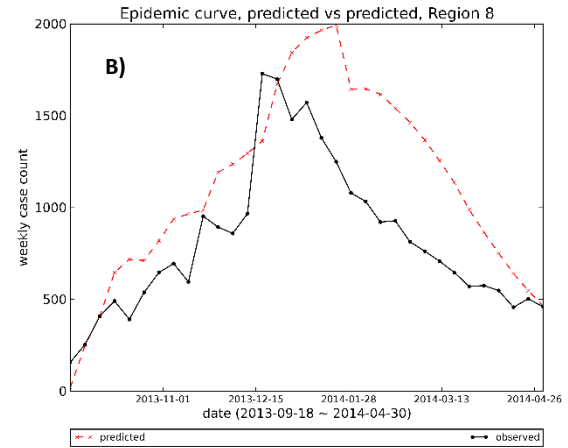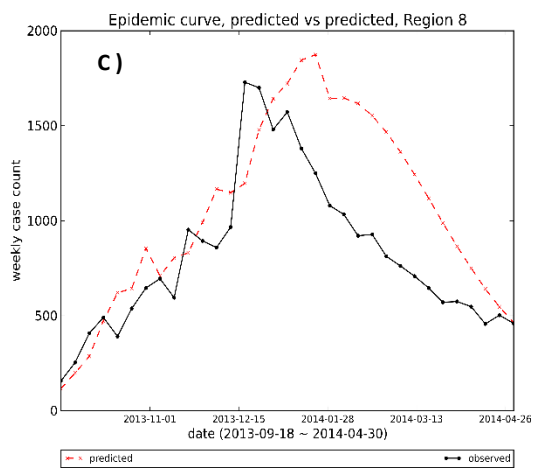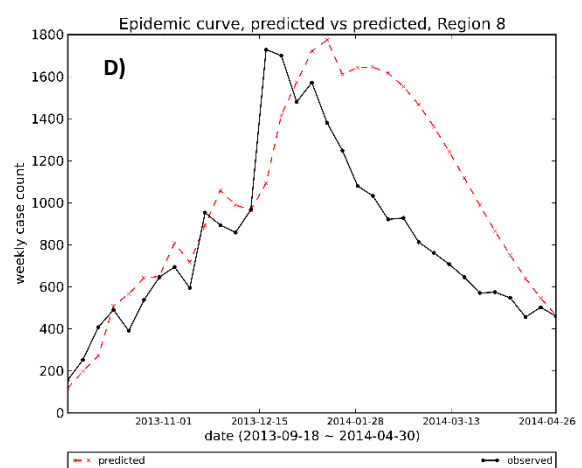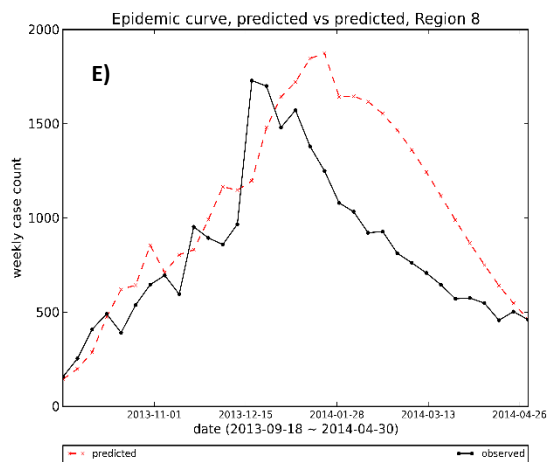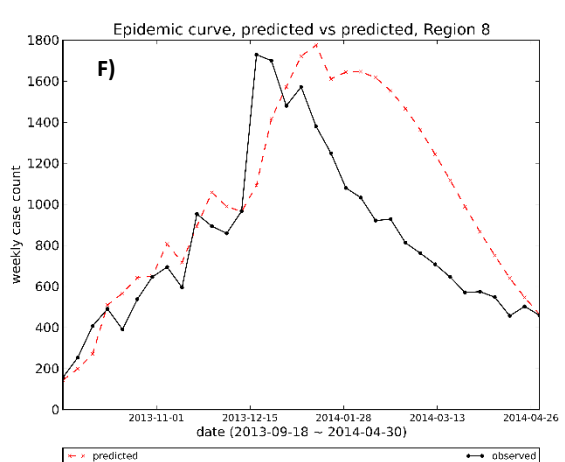

Supplement: Supplementary file 19 — Visual comparison of 1-step-ahead predicted curves generated by six methods vs. the observed curve, Region 8. (PDF 413 kb) [file 12879_2017_2365_MOESM19_ESM.pdf]

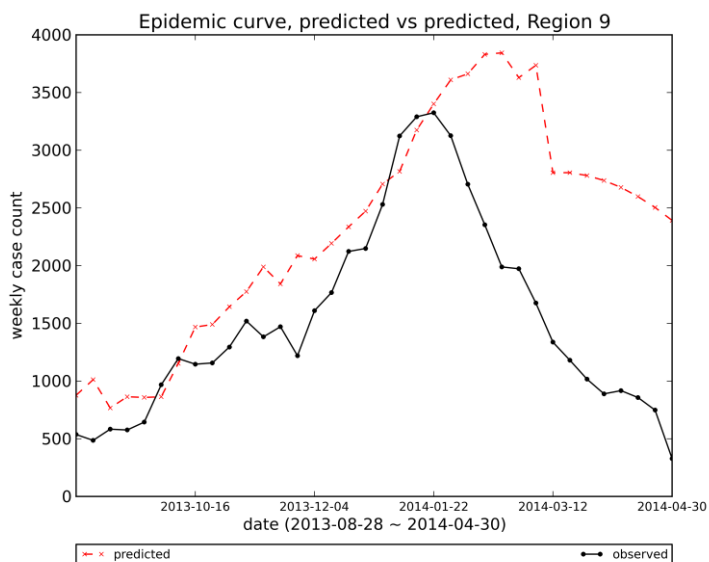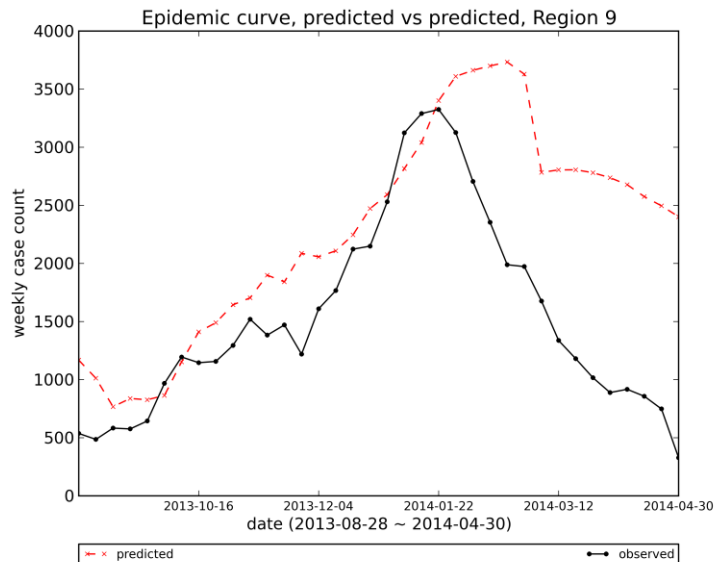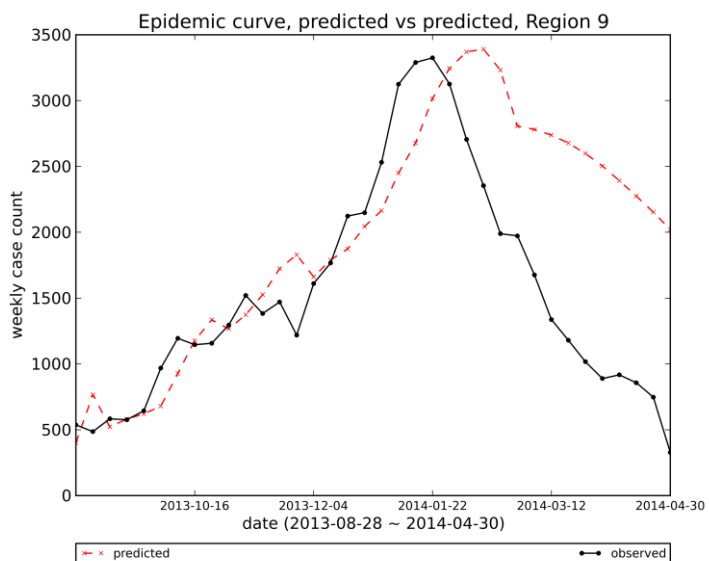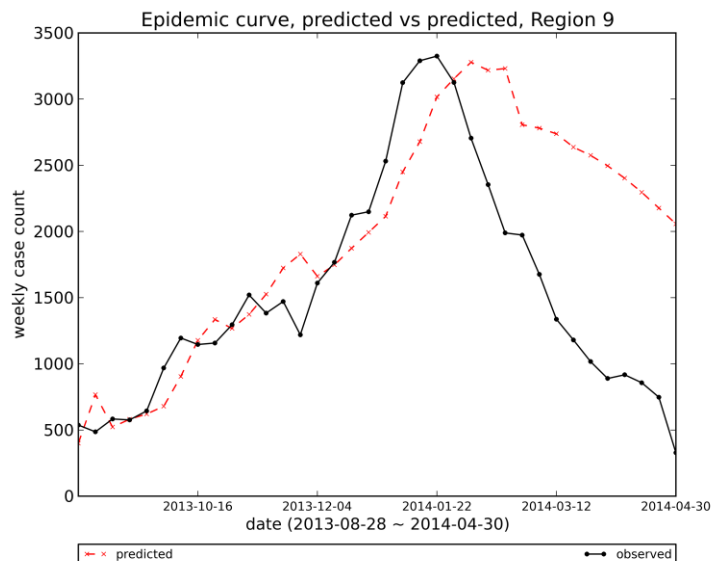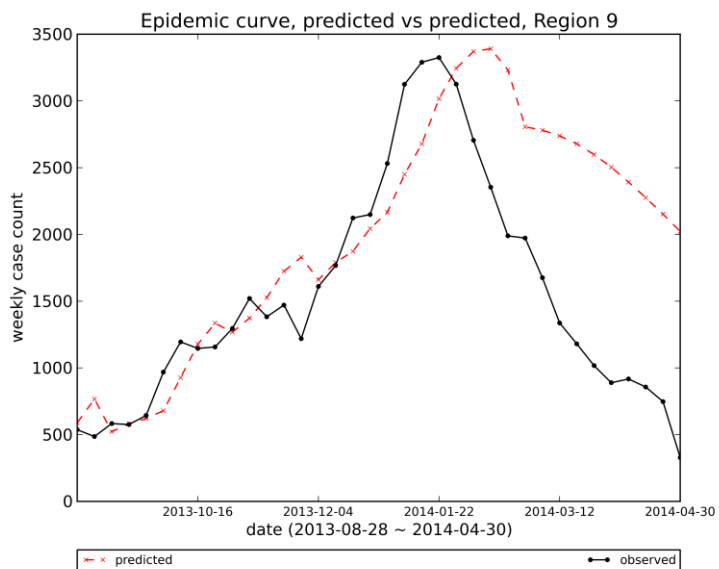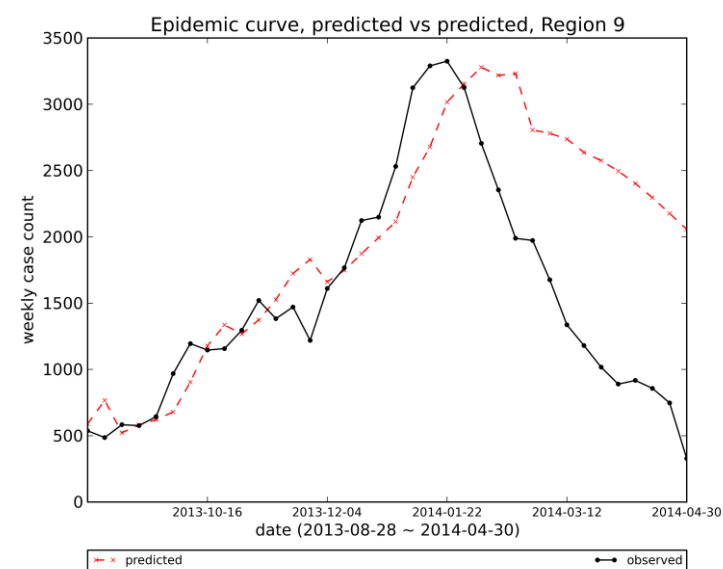

Supplement: Supplementary file 20 — Visual comparison of 1-step-ahead predicted curves generated by six methods vs. the observed curve, Region 9. (PDF 389 kb) [file 12879_2017_2365_MOESM20_ESM.pdf]

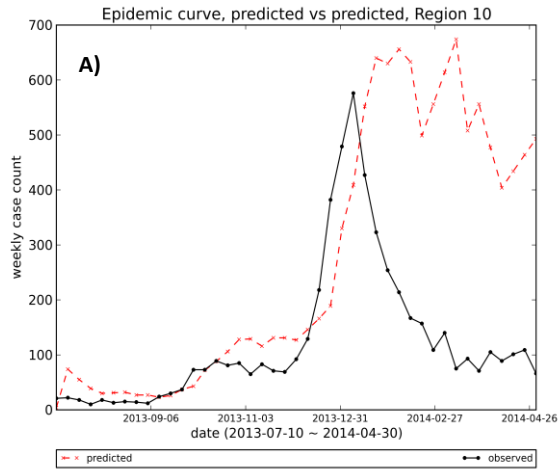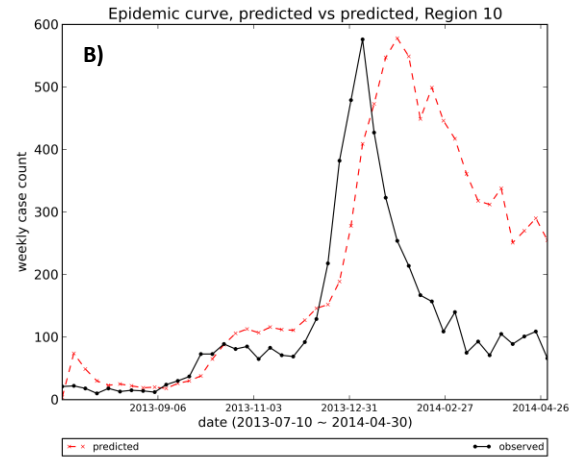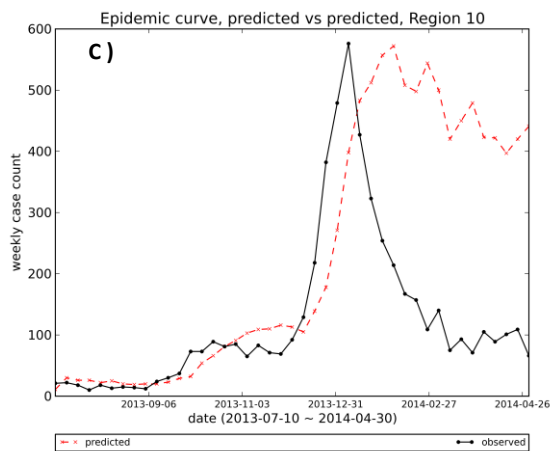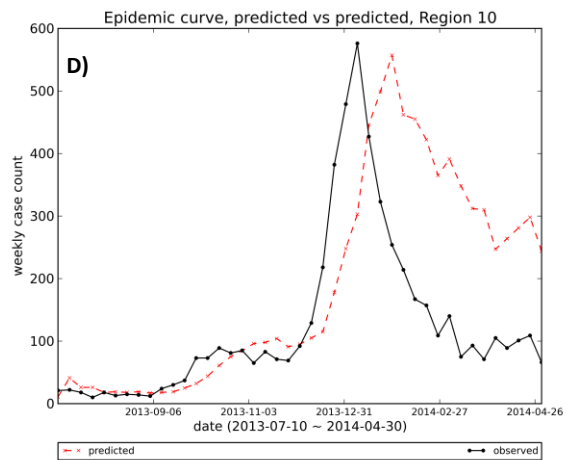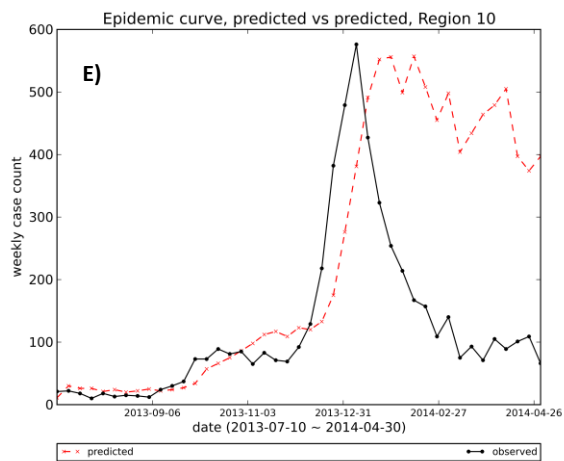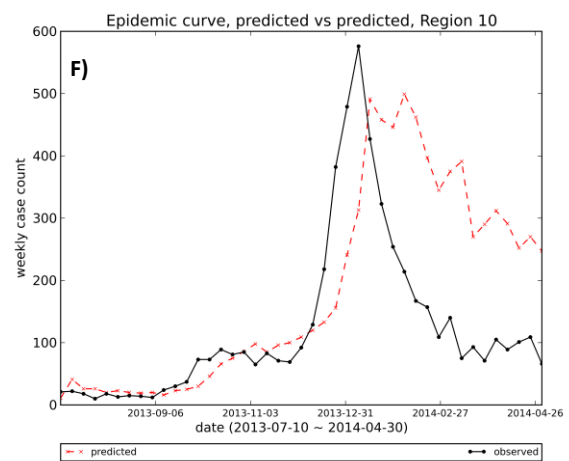

Supplement: Supplementary file 21 — Visual comparison of 1-step-ahead predicted curves generated by six methods vs. the observed curve, Region 10. (PDF 514 kb) [file 12879_2017_2365_MOESM21_ESM.pdf]
