# Supplementary material for: A framework for evaluating epidemic forecasts
Source: BMC Infect Dis. 2017 May 15;17:345. doi: 10.1186/s12879-017-2365-1 (PMC5433189; doi:10.1186/s12879-017-2365-1)
Supplement: Supplementary file 4 — Consensus Ranking of forecasting methods over all error measures for predicting different Epi-features for Region 2. (PDF 289 kb) [file 12879_2017_2365_MOESM4_ESM.pdf]

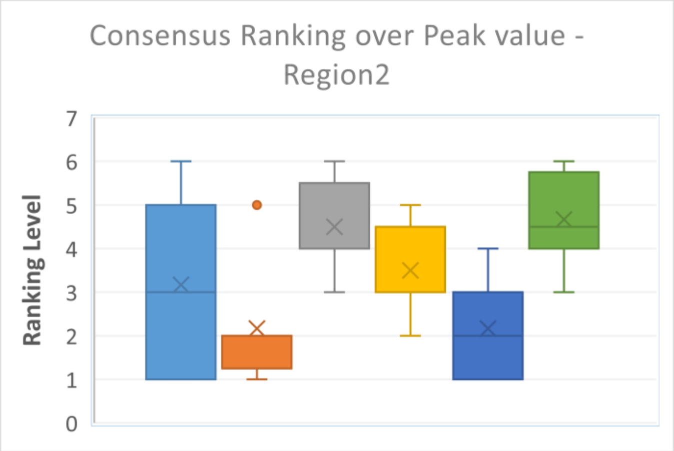
Consensus Ranking over Peak value - Region2
Ranking Level
0 1 2 3 4 5 6 7

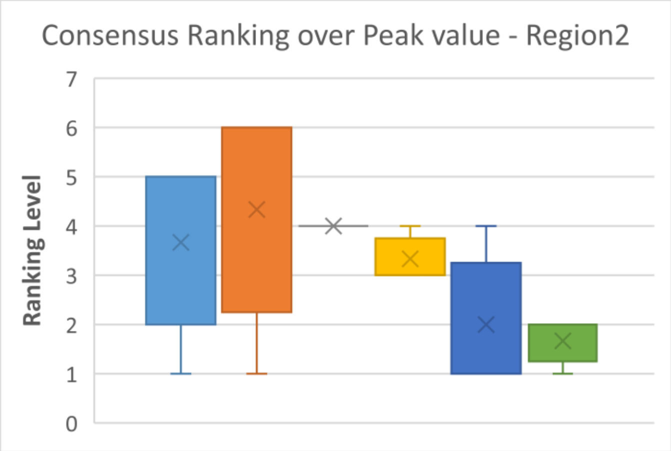
Consensus Ranking over Peak value - Region2
Ranking Level
0 1 2 3 4 5 6 7

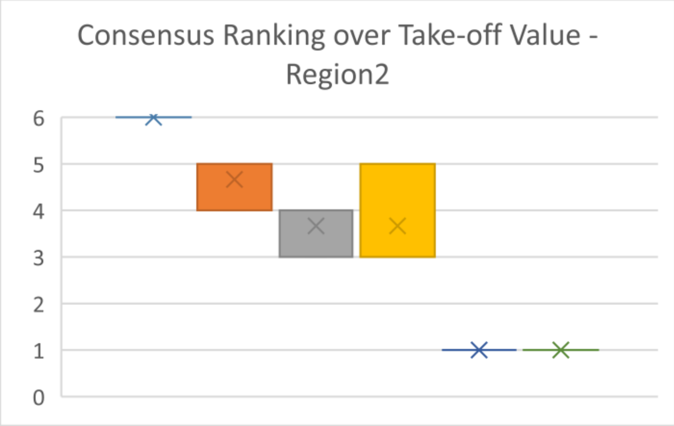
Consensus Ranking over Take-off Value - Region2
0 1 2 3 4 5 6

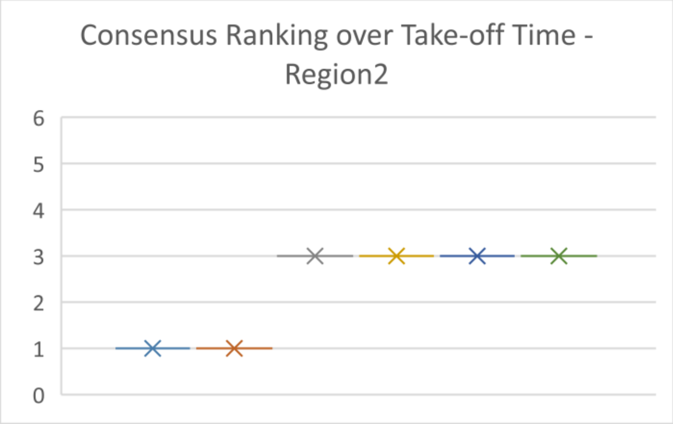
Consensus Ranking over Take-off Time - Region2
0 1 2 3 4 5 6

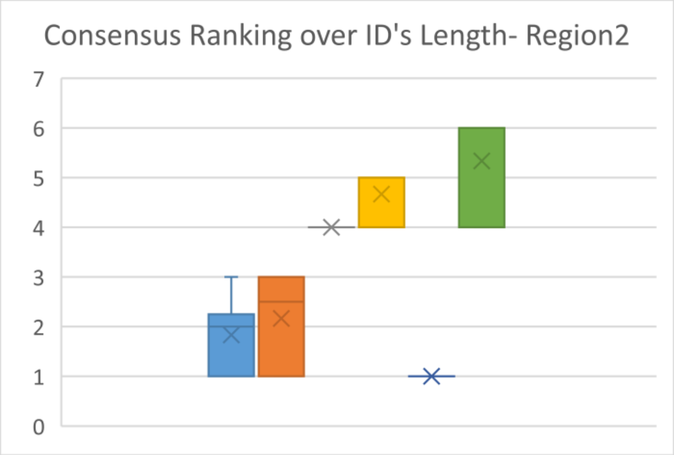
Consensus Ranking over ID's Length- Region2
0 1 2 3 4 5 6 7

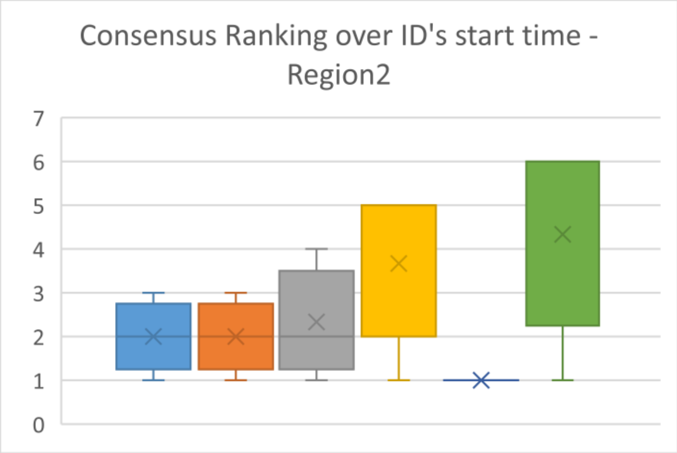
Consensus Ranking over ID's start time - Region2
0 1 2 3 4 5 6 7

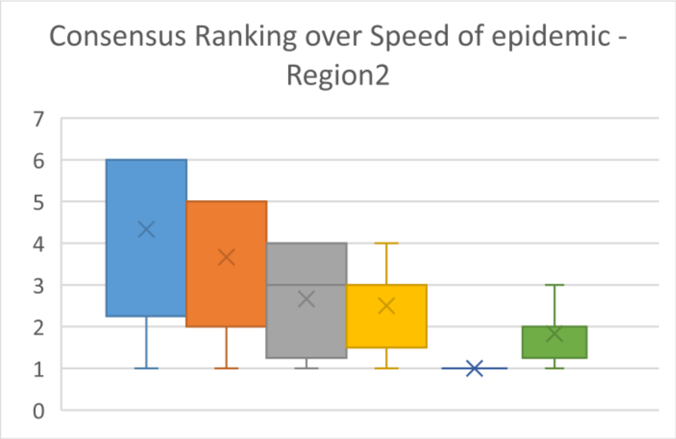
Consensus Ranking over Speed of epidemic - Region2
0 1 2 3 4 5 6 7

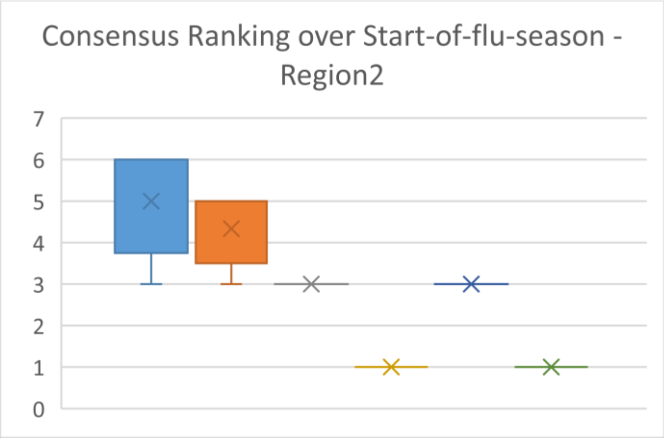
Consensus Ranking over Start-of-flu-season - Region2
0 1 2 3 4 5 6 7
