# Supplementary material for: A framework for evaluating epidemic forecasts
Source: BMC Infect Dis. 2017 May 15;17:345. doi: 10.1186/s12879-017-2365-1 (PMC5433189; doi:10.1186/s12879-017-2365-1)
Supplement: Supplementary file 5 — Consensus Ranking of forecasting methods over all error measures for predicting different Epi-features for Region 3. (PDF 286 kb) [file 12879_2017_2365_MOESM5_ESM.pdf]

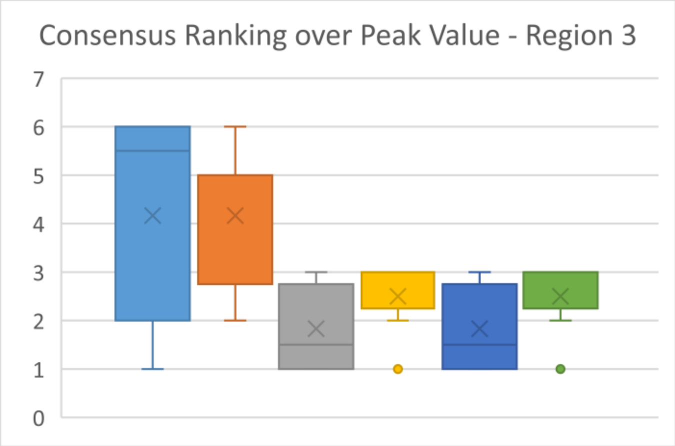
Consensus Ranking over Peak Value - Region 3
7
6
5
4
3
2
1
0

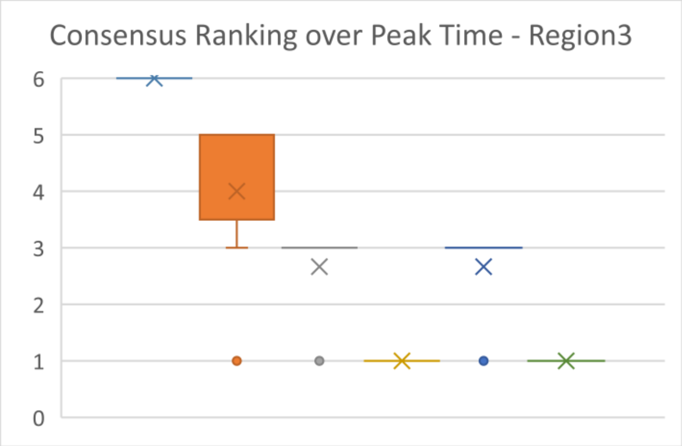
Consensus Ranking over Peak Time - Region3
6
5
4
3
2
1
0

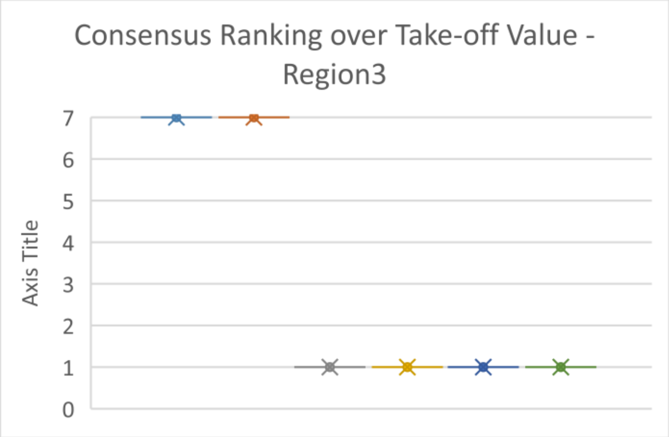
Consensus Ranking over Take-off Value - Region3
Axis Title
7
6
5
4
3
2
1
0

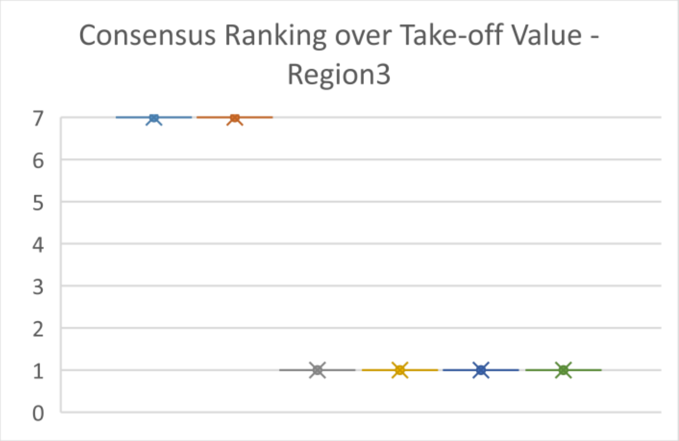
Consensus Ranking over Take-off Value - Region3
7
6
5
4
3
2
1
0

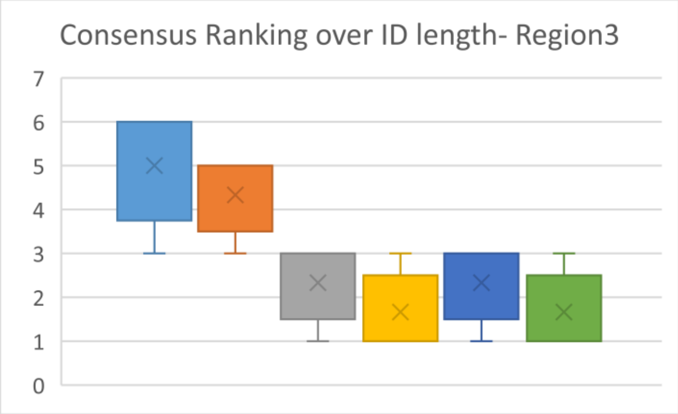
Consensus Ranking over ID length- Region3
7
6
5
4
3
2
1
0

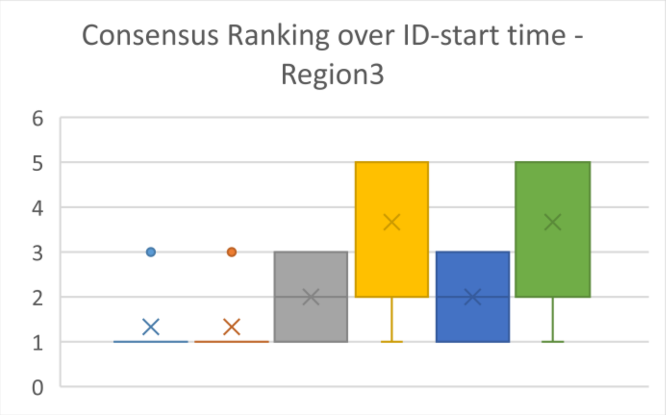
Consensus Ranking over ID-start time - Region3
6
5
4
3
2
1
0

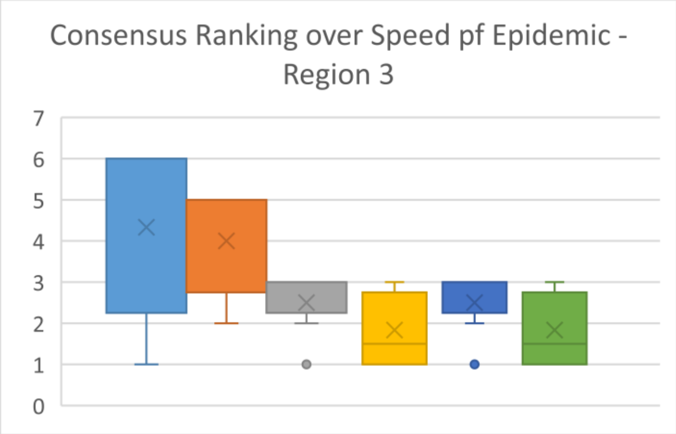
Consensus Ranking over Speed pf Epidemic - Region 3
7
6
5
4
3
2
1
0

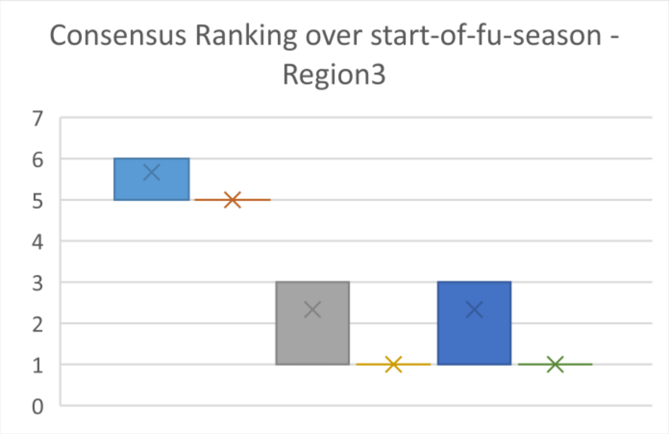
Consensus Ranking over start-of-fu-season - Region3
7
6
5
4
3
2
1
0
