# Supplementary material for: A framework for evaluating epidemic forecasts
Source: BMC Infect Dis. 2017 May 15;17:345. doi: 10.1186/s12879-017-2365-1 (PMC5433189; doi:10.1186/s12879-017-2365-1)
Supplement: Supplementary file 6 — Consensus Ranking of forecasting methods over all error measures for predicting different Epi-features for Region 4. (PDF 281 kb) [file 12879_2017_2365_MOESM6_ESM.pdf]

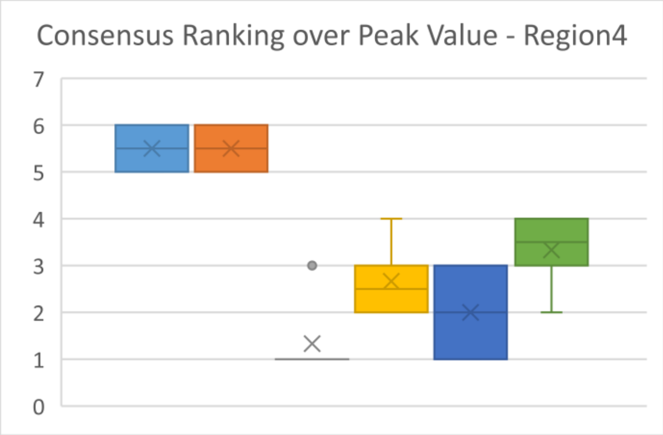
Consensus Ranking over Peak Value - Region4
7
6
5
4
3
2
1
0

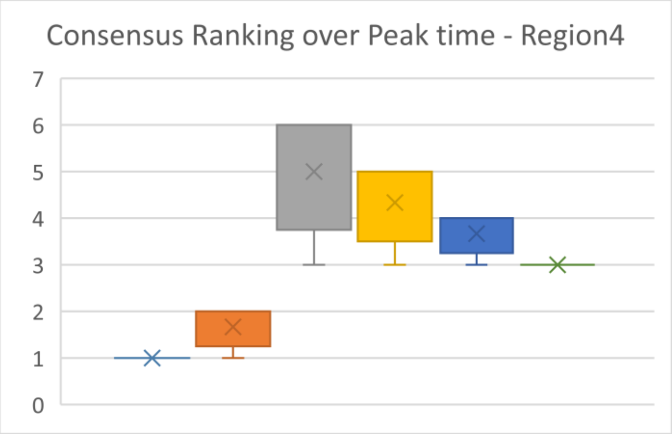
Consensus Ranking over Peak time - Region4
7
6
5
4
3
2
1
0

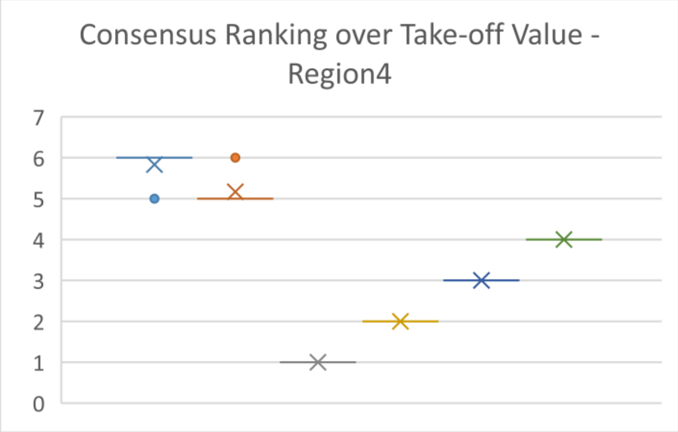
Consensus Ranking over Take-off Value - Region4
7
6
5
4
3
2
1
0

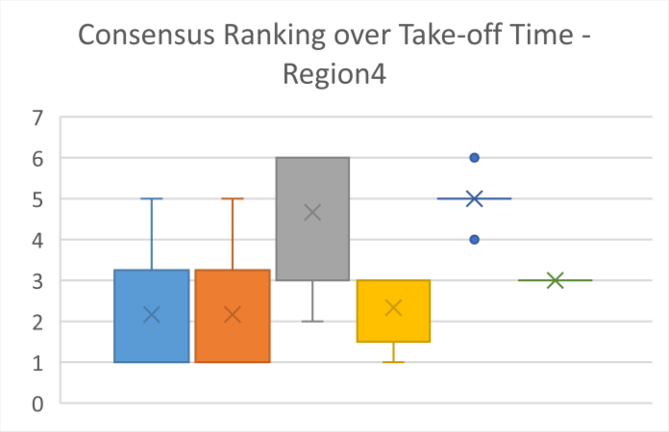
Consensus Ranking over Take-off Time - Region4
7
6
5
4
3
2
1
0

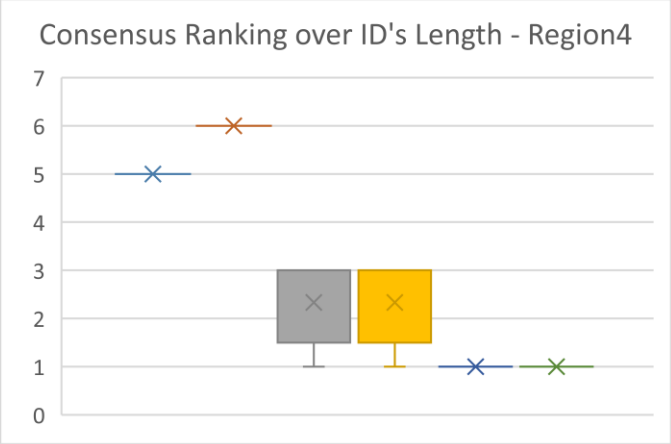
Consensus Ranking over ID's Length - Region4
7
6
5
4
3
2
1
0

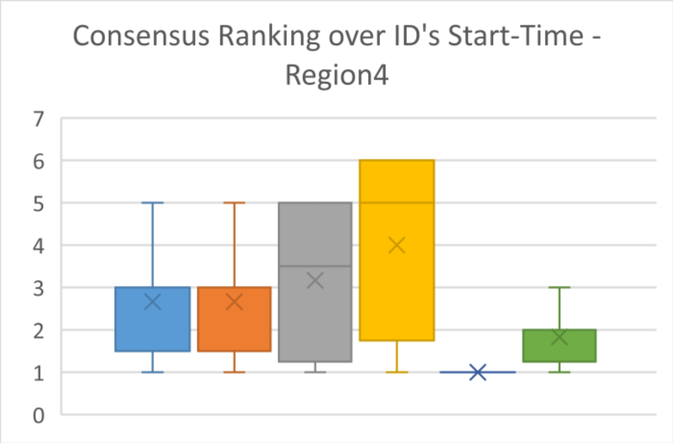
Consensus Ranking over ID's Start-Time - Region4
7
6
5
4
3
2
1
0

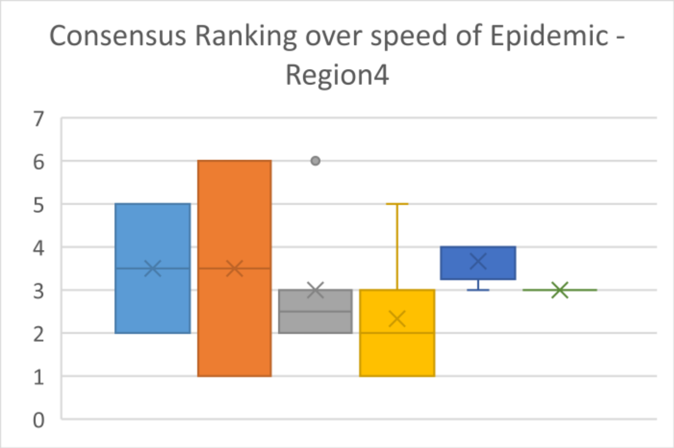
Consensus Ranking over speed of Epidemic - Region4
7
6
5
4
3
2
1
0

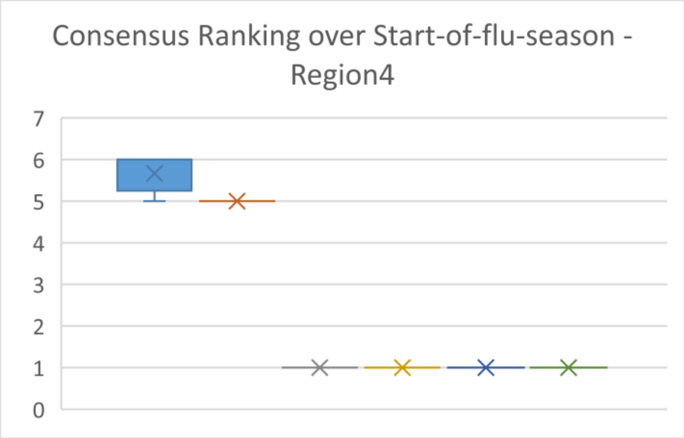
Consensus Ranking over Start-of-flu-season - Region4
7
6
5
4
3
2
1
0
