# Supplementary material for: A framework for evaluating epidemic forecasts
Source: BMC Infect Dis. 2017 May 15;17:345. doi: 10.1186/s12879-017-2365-1 (PMC5433189; doi:10.1186/s12879-017-2365-1)
Supplement: Supplementary file 10 — Consensus Ranking of forecasting methods over all error measures for predicting different Epi-features for Region 8. (PDF 283 kb) [file 12879_2017_2365_MOESM10_ESM.pdf]

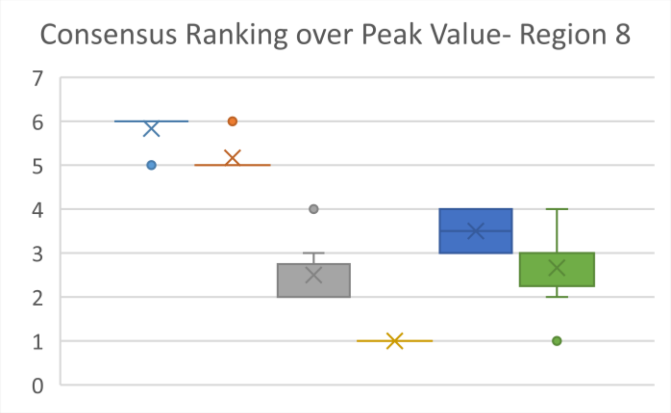
Consensus Ranking over Peak Value- Region 8
7
6
5
4
3
2
1
0

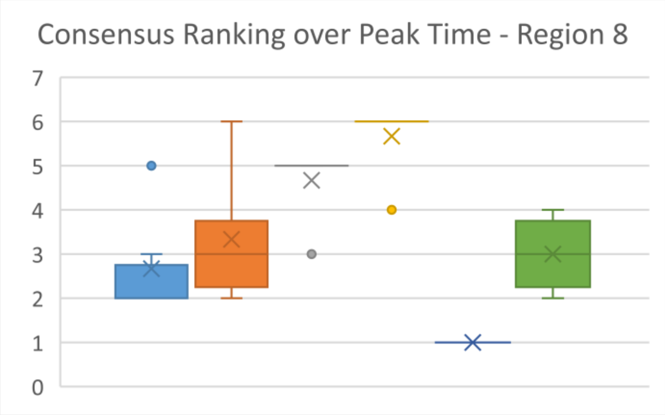
Consensus Ranking over Peak Time - Region 8
7
6
5
4
3
2
1
0

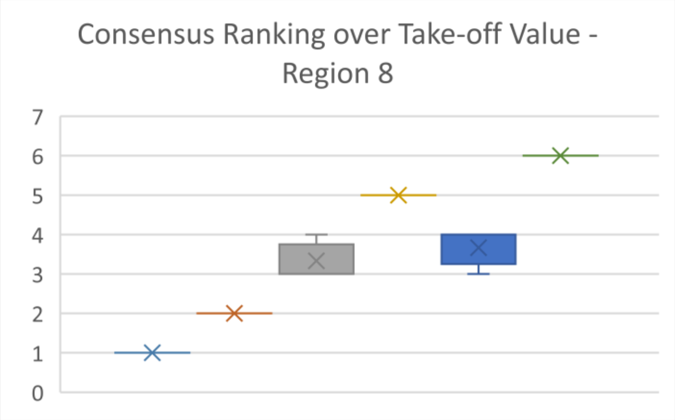
Consensus Ranking over Take-off Value - Region 8
7
6
5
4
3
2
1
0

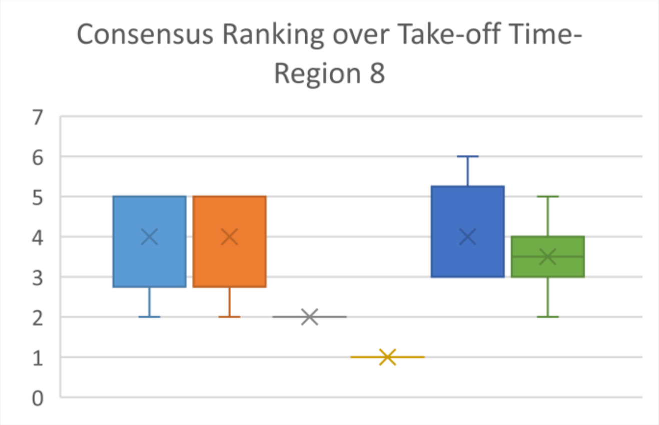
Consensus Ranking over Take-off Time- Region 8
7
6
5
4
3
2
1
0

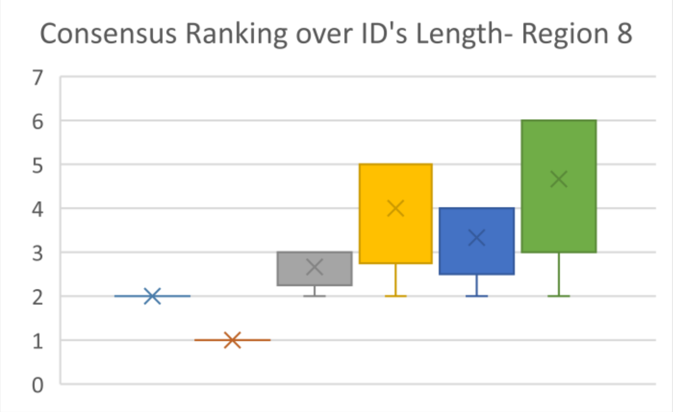
Consensus Ranking over ID's Length- Region 8
7
6
5
4
3
2
1
0

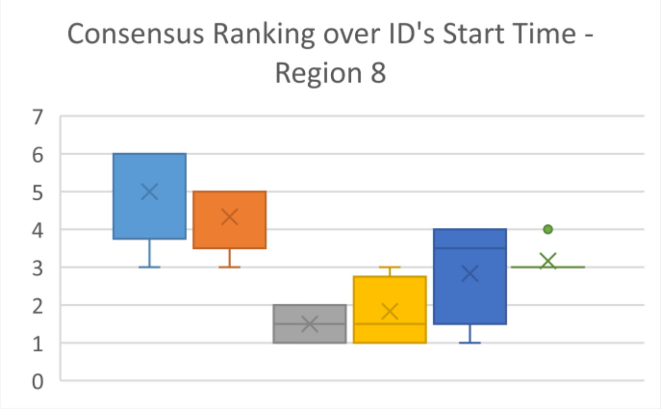
Consensus Ranking over ID's Start Time - Region 8
7
6
5
4
3
2
1
0

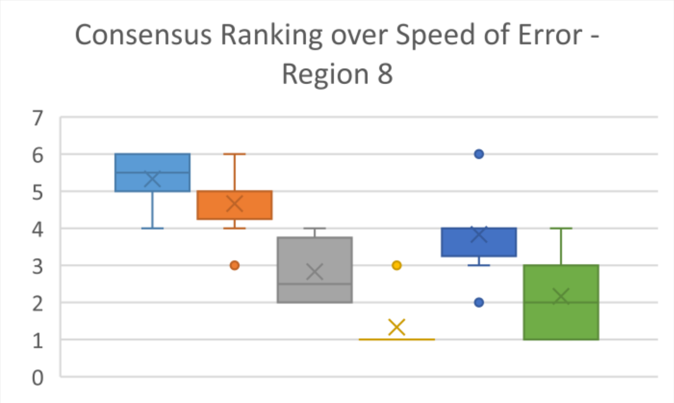
Consensus Ranking over Speed of Error - Region 8
7
6
5
4
3
2
1
0

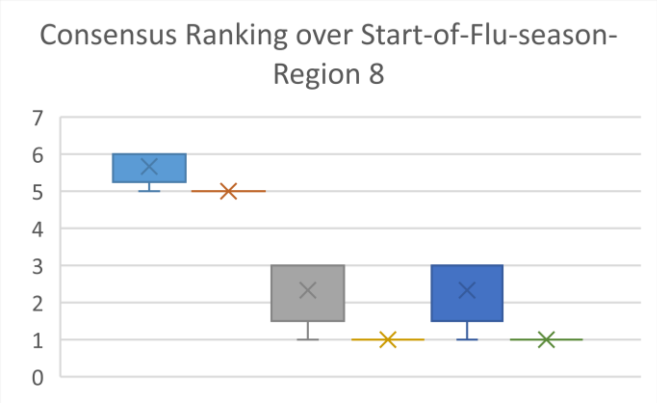
Consensus Ranking over Start-of-Flu-season- Region 8
7
6
5
4
3
2
1
0
